# The molecular framework balancing growth and defense in response to plant elicitor peptide-induced signals in Arabidopsis

**DOI:** 10.1093/plcell/koae327

**Published:** 2024-12-19

**Authors:** Souvik Dhar, Soo Youn Kim, Hee-Ji Shin, Jongsung Park, Ji-Young Lee

**Affiliations:** School of Biological Sciences, College of Natural Science, Seoul National University, 1 Gwanak-ro, Gwanak-gu, Seoul 08826, South Korea; School of Biological Sciences, College of Natural Science, Seoul National University, 1 Gwanak-ro, Gwanak-gu, Seoul 08826, South Korea; School of Biological Sciences, College of Natural Science, Seoul National University, 1 Gwanak-ro, Gwanak-gu, Seoul 08826, South Korea; School of Biological Sciences, College of Natural Science, Seoul National University, 1 Gwanak-ro, Gwanak-gu, Seoul 08826, South Korea; School of Biological Sciences, College of Natural Science, Seoul National University, 1 Gwanak-ro, Gwanak-gu, Seoul 08826, South Korea; Plant Genomics and Breeding Institute, Seoul National University, 1 Gwanak-ro, Gwanak-gu, Seoul 08826, South Korea; Plant Immunity Research Center, Seoul National University, 1 Gwanak-ro, Gwanak-gu, Seoul 08826, South Korea

## Abstract

Elevated stress signaling compromises plant growth by suppressing proliferative and formative division in the meristem. Plant elicitor peptide, an endogenous danger signal triggered by biotic and abiotic stresses in Arabidopsis (*Arabidopsis thaliana*), suppresses proliferative division, alters xylem vessel organization, and disrupts cell-to-cell symplastic connections in roots. To gain insight into the dynamic molecular framework that modulates root development under elevated danger signals, we performed a time-course RNA-sequencing analysis of the root meristem after synthetic PEP1 treatment. Our analyses revealed that SALT TOLERANCE ZINC FINGER (STZ) and its homologs are a potential nexus between the stress response and proliferative cell cycle regulation. Through functional, phenotypic, and transcriptomic analyses, we observed that STZ differentially controls the cell cycle, cell differentiation, and stress response genes in various tissue layers of the root meristem. Moreover, we determined the *STZ* expression level critical for enabling the growth–defense tradeoff. These findings provide valuable information about the dynamic gene expression changes that occur upon perceiving danger signals in the root meristem and potential engineering strategies to generate stress-resilient plants.

## Teaser

The C2H2 zinc finger transcription factor SALT TOLERANCE ZINC FINGER and its homologs balance root growth and stress tolerance in response to endogenous danger signals.

## Introduction

As sessile organisms, plants use strategies that are distinct from those of animals when they encounter biotic and/or abiotic stressors. Plants quickly invest in defense and stress tolerance programs to survive at the expense of growth (Huot et al. 2014; He et al. 2022). For this transition, stress tolerance, starting with a complex exchange of plant–environmental signals at the cellular level, should propagate to the entire organ and body and suppress the growth program in meristems (Figueroa-Macias et al. 2021). Understanding the dynamics of the perception and propagation of stress signals and their downstream transcriptional framework would improve our understanding of how plants balance growth and stress responses and help develop sustainable crops under environmental changes.

Biotic and abiotic stress signals from the environment trigger the production of “danger signals” in cells. Danger signals in the form of small metabolites or peptides are secreted outside the cell and sensed by neighbors, thereby activating stress responses. Plant elicitor peptides (PEPs) are endogenous danger signals that are produced in response to biotic and abiotic stressors and enhance stress tolerance (Huffaker et al. 2006, 2013; Ross et al. 2014; Yamada et al. 2016; Nakaminami et al. 2018; Hander et al. 2019). In Arabidopsis (*Arabidopsis thaliana*), synthetic PEP1 treated to seedlings suppresses apical root growth, triggers the formation of excessive root hairs, and alters the vascular tissue arrangement within the stele (Poncini et al. 2017; Dhar et al. 2021; Okada et al. 2021). However, how PEP transcriptionally modulates root development and whether this is a part of the growth–defense tradeoff remain to be elucidated.

Transcriptional gene regulatory networks (GRNs) are essential for stress response at various levels. Many transcription factors (TFs) involved in stress responses have been identified, and GRNs involving these TFs have been actively investigated (Han et al. 2020b). Among the more than 2,000 TFs in Arabidopsis, zinc finger proteins form one of the largest TF families and are divided into various subfamilies based on the position of Cys and His residues in their secondary structures (Miller et al. 1985; Klug 2010; Han et al. 2014). The C2H2-type zinc finger TF family, which includes 176 genes in Arabidopsis (Englbrecht et al. 2004; Xie et al. 2019), has been reported to function in plant growth and development, biotic and abiotic stress resistance (Han et al. 2020a), and programmed cell death (PCD) (Feng et al. 2023).

In this study, we used synthetic PEP1 as a mediator that triggers a broad range of stress responses similar to that induced by biotic and abiotic stressors and then investigated GRNs downstream of PEP signaling to identify key TFs controlling stress response and root growth and development. Our results suggest that SALT TOLERANCE ZINC FINGER (STZ/ZAT10) and the related C2H2 zinc finger TFs, including ZINC FINGER OF ARABIDOPSIS THALIANA 6 (ZAT6) and ZINC FINGER PROTEIN 3 (AZF3), serve as a nexus between the stress response and root apical growth. PEP1 rapidly induces the expression of these TFs in root apical meristems. Transgenic roots overexpressing *STZ* phenocopied roots treated with PEP1, which had reduced meristem activity and expanded xylem differentiation domain (Dhar et al. 2021). This phenotype was consistent with changes in transcriptomes in response to STZ in the root meristem, in which cell cycle genes were suppressed, whereas PCD genes involved in xylem differentiation and stress response TFs were activated. In this regulation, suppressing cell cycle genes required a higher STZ dosage than activating stress response TF genes. These findings emphasize the significance of PEP signaling in controlling TF action to balance growth and defense and provide potential applications in sustainable crop plant growth under stressful conditions.

## Results

### PEP1-mediated transcriptional changes in the root meristem

**Figure 1.**
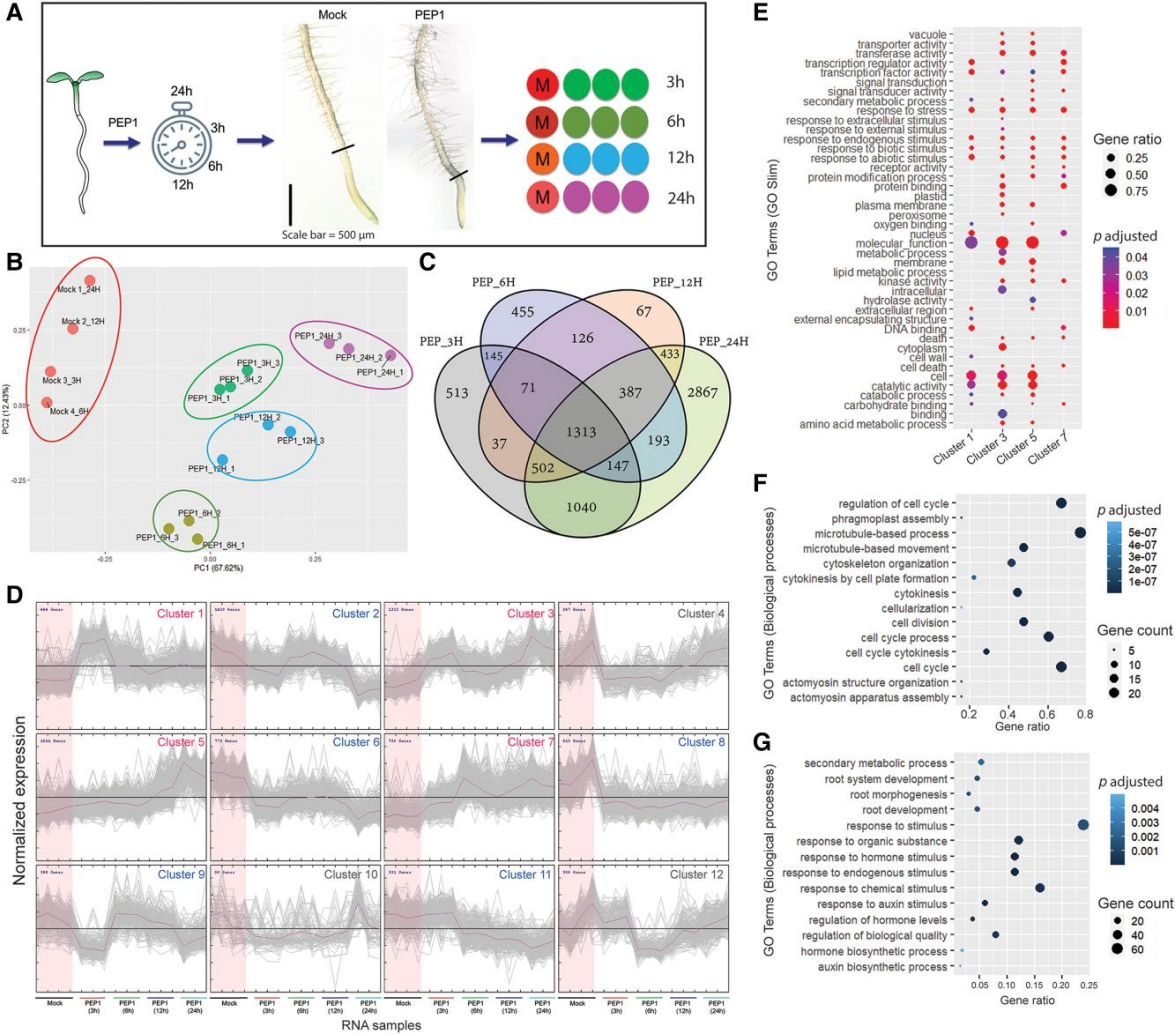
Identification of PEP1-influenced meristem-specific regulatory cascades through a time-course RNA sequencing. **A)** Schematic representation of the RNA-seq experiment. We used 4 different time points (3, 6, 12, and 24 h) to harvest meristem samples for RNA isolation. Representative 5 DAT root tips treated with (PEP1) or without (mock) 1 *µ*m PEP1 for 24 h are shown. Scale bar = 500 *µ*m (applicable to both root images). **B)** PCA of 16 RNA-seq samples based on the top 5,000 most DEGs. **C)** A comparison of DEGs (FDR < 0.01, |fold change| ≥ 1.5) identified in this study. Comparison was made between the 3-, 6-, 12-, and 24-h post-PEP1-treated root meristem samples. A total of 8,296 unique DEGs were identified and are listed in Supplementary Table S3. **D)** K-means clustering of the 8,296 DEGs were grouped into 12 clusters (Supplementary Table S4). The induced clusters, 1, 3, 5, and 7, are marked in red, the suppressed clusters, 2, 6, 8, 9, and 11, are marked in blue, and the clusters, 4, 10, and 12, not used for further analysis in this study are marked in gray. The gray plots in each cluster indicate the normalized expression dynamics of individual genes, and the purple plot represents a mean expression dynamic. **E)** GOSlim enrichment analysis (FDR < 0.05) of PEP1-induced DEGs from Clusters 1, 3, 5, and 7 in **D)**. The total number of DEGs in these clusters and the respective GO (biological processes) enrichment analyses are presented in Supplementary Tables S5 and S6. **F and G)** Dot plots represent GO (biological processes) enrichment analysis (FDR < 0.01) of the PEP1-suppressed DEGs from Clusters 9 and 11 in **D)**. Notably, among the PEP1-suppressed clusters, Clusters 9 and 11 were enriched for cell cycle and root development, respectively. The top 14 GO terms in each cluster are listed. A full list of enriched GO terms for each cluster is presented in Supplementary Table S5. DAT, days after transfer to the growth chamber.

Synthetic PEP1 drastically suppresses apical root growth by inhibiting proliferative cell divisions (Poncini et al. 2017; Jing et al. 2019; Dhar et al. 2021). We observed a clear reduction in meristem size as early as 6 h after PEP1 treatment (Supplementary Fig. S1). We also observed that PEP1 influences the formative division of vascular initials in the meristem (Dhar et al. 2021). To understand the genes and regulatory pathways that PEP1 directs for cellular reprogramming in the root meristem, we performed time-course transcriptome profiling of PEP1-treated root meristems (Fig. 1A). Root meristems were dissected from 5-d-old Arabidopsis Col-0 seedlings that were treated with 1 *μ*m of synthetic PEP1 for 3, 6, 12, and 24 h. The total RNA extracted from these samples was processed for sequencing using the Illumina NovaSeq6000 system.

Over 800 million high-quality clean RNA-seq paired-end reads were generated from 16 samples (Supplementary Table S1). The expression levels (reads per kilobase of transcript per million mapped reads, or RPKM) of all annotated transcripts are presented in Supplementary Table S2. Principal component analysis (PCA) of the top 5,000 most variable genes revealed that the integrity of the mock samples remained sufficiently distinct to be separated from the PEP1-treated samples by PC1, although each mock sample was collected at different time points (Fig. 1B). We identified a total of 8,296 differentially expressed genes (DEGs; fold change [FC] ≥ 1.5 and false discovery rate [FDR] < 0.01) in PEP1-treated samples compared with the mock samples (Fig. 1C; Supplementary Table S3). Unique DEGs were significantly increased 24 h after PEP1 treatment (Supplementary Fig. S2 and Table S3). To detect PEP1-dependent transcriptome reprogramming over time, we clustered the expression patterns of 8,296 DEGs using the K-means clustering algorithm (Lloyd 1982; Fig. 1D; Supplementary Table S4). Of the 12 clusters, Clusters 1, 3, 5, and 7 exhibited PEP1-induced “rapid transient,” “rapid stable,” “late,” and “late stable” expression dynamics, respectively. Among the PEP1-repressed clusters, Cluster 2 showed “late,” Clusters 6 and 8 showed “rapid stable,” Cluster 9 exhibited “rapid transient,” and Cluster 11 displayed “late stable” suppression.

To determine which biological processes are affected by PEP1 in the root meristem, we conducted a gene ontology (GO) enrichment analysis (FDR ≤ 0.01) for the 9 clusters mentioned above, showing explicit expression dynamics in response to PEP1 (Supplementary Table S5). Because of the high volume of overrepresented GO terms, we performed GOSlim categorization for the PEP1-induced clusters to obtain a broader overview of GO content (Fig. 1E; Supplementary Table S6). Expectedly, clusters with genes induced by PEP1 were enriched with those involved in responses to various biotic and abiotic stimuli. Among the 5 clusters with genes downregulated under PEP1 treatment, Clusters 9 and 11 were enriched with genes that regulated the cell cycle and root development (Fig. 1, F and G). Cluster 9, composed of 388 DEGs, displayed significant enrichment of GO terms related to “regulation of cell cycle” (GO:0051726), “cell cycle” (GO:0007049), and “cell division (GO:0051301)” (Fig. 1F; Supplementary Table S5). Moreover, the GO term analysis results from Cluster 11 revealed the presence of significantly enriched DEGs involved in “root development” (GO:0048364) and “morphogenesis” (GO:0010015) (Fig. 1G; Supplementary Table S5). Because root meristem activities for growth are perturbed by exposure to PEP1 (Supplementary Fig. S1; Dhar et al. 2021), we focused on Clusters 9 and 11 for further analysis.

We visualized the root cell-type-specific expression patterns of genes belonging to Clusters 9 and 11 (Brady et al. 2007; Zhang et al. 2019). Most genes rapidly suppressed by PEP1 in Cluster 9 showed enriched expressions deep inside the roots, such as the xylem (S4 and S18) (Supplementary Fig. S3A). Genes in Cluster 11, enriched with genes involved in root development and morphogenesis, were expressed in various cell types (Supplementary Fig. S3B).

To identify the TFs induced by PEP1, we combined all DEGs from either the “transcription regulator activity” (GO:0030528) or “transcription factor activity” (GO:0003700) terms, in Clusters 1, 3, 5, and 7 (Fig. 1E; Supplementary Fig. S4, A and B and Table S7). In total, 344 TFs were identified and classified into 43 distinct classes based on their protein domains using the InterPro database (https://www.ebi.ac.uk/interpro) (Supplementary Fig. S4C and Table S8). We observed that stress-induced ERF, MYB, WRKY, and zinc finger (ZF) domain TFs were enriched in the PEP1-induced clusters. Their root cell-type expression patterns (Brady et al. 2007; Zhang et al. 2019) indicated a significant enrichment of PEP1-induced transcriptional regulation inside the stele (Supplementary Fig. S4D).

### Identification of TFs controlling the PEP signaling-dependent reprogramming of root development

To identify the key TFs that trigger root development reprogramming in response to PEP1, we further investigated 344 TFs induced by PEP1 (Supplementary Fig. S4 and Table S7). Using the STRING database (version 12.0; https://string-db.org/; Szklarczyk et al. 2015), we queried the interaction networks of the 344 TFs (interaction score > 0.7). A total of 225 interactions were identified among 159 TFs (Supplementary Table S9). In the most prominent network composed of 94 TFs with 162 interactions, STZ, ZINC FINGER OF ARABIDOPSIS THALIANA 12 (ZAT12), WRKY DNA-BINDING PROTEIN 33 (WRKY33), WRKY DNA-BINDING PROTEIN 40 (WRKY40), and DEHYDRATION-RESPONSIVE ELEMENT BINDING PROTEIN 2A (DREB2A) were the most highly connected (Supplementary Fig. S5A).

**Figure 2.**
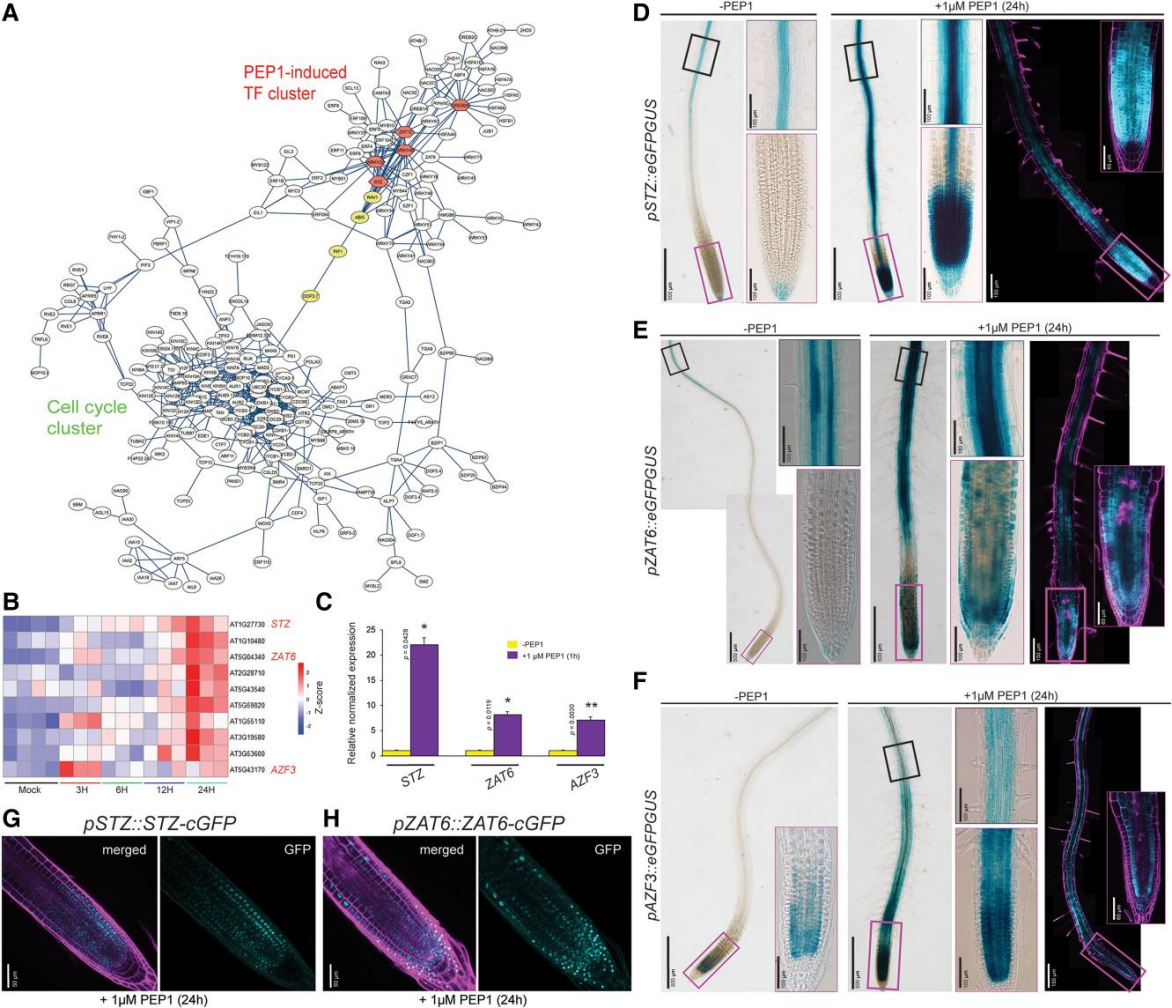
STZ and related C2H2 zinc finger TFs are a potential nexus between PEP1-induced and suppressed genes. **A)** Interaction network of PEP1-induced TFs together with the PEP1-suppressed cell cycle cluster as inferred from the STRING database (version 12.0; interaction score > 0.7) (Szklarczyk et al. 2015). The interaction scores are presented in Supplementary Table S10. Nodes highlighted in “red” represent the top 5 highly connected TFs among the 344 PEP1-induced TFs. The nodes highlighted in “yellow” represent the intermediary TFs from the most highly connected PEP1-induced TFs to the cell cycle cluster, based on the STRING database. The network was visualized using Cytoscape (version 3.10.1; www.cytoscape.org). **B)** A heatmap of the expression patterns of the 10 C2H2 zinc finger (ZF) TFs is presented in Supplementary Table S8. These ZF TFs were induced early in the seedlings treated with PEP1. The color scale bars denote the *Z*-score as obtained through the “scale” function while plotting using the “pheatmap” package in R. **C)** Expression levels of the 3 selected TFs analyzed by RT-qPCR. The 4 DAT seedlings were treated with 1 *µ*m of PEP1 for 1 h, and the bottom half of their roots were dissected to extract total RNA. Three technical replicates were run for each sample, and the fold changes were determined and compared with the mock sample (-PEP1). The expression values are presented as standard errors of the mean (SEM). The statistical significance was determined using a Student's *t*-test (**P < 0.01, *P < 0.05). **D to F)** Expression patterns of *STZ*, *ZAT6*, and *AZF3* in the 5 DAT WT Col-0 plants with or without 1 *µ*m PEP1 treatment for 24 h. The transcriptional expression patterns of these genes were visualized using GUS or GFP (*pSTZ::eGFPGUS*, *pZAT6::eGFPGUS*, *pAZF3::eGFPGUS*). The confocal root images shown here are composite images obtained through tile scanning. **G and H)** The proteins of *STZ* (STZ-cGFP) and *ZAT6* (ZAT6-cGFP) in the seedlings at 5 DAT of translational fusion lines, *pSTZ::STZ-cGFP* and *pZAT6::ZAT6-cGFP*, treated with 1 *µ*m PEP1 for 24 h. No GFP localization signal was observed in the meristems of untreated samples (Supplementary Fig. S6). GFP: cyan; PI: magenta. DAT, days after transfer to the growth chamber.

PEP1 rapidly suppressed apical root growth, likely by repressing cell cycle progression. This was indicated by the overrepresentation of cell cycle-related genes in Cluster 9 (Fig. 1F). Consistently, our assessment of cell proliferation using the plant cell cycle indicator (PlaCCI; Desvoyes et al. 2020) showed a significant reduction in markers related to cell division (CYCB1;1 and CDT1a) in the meristems treated with PEP1 for 24 h (Supplementary Fig. S5B). Since PEP1-mediated suppression of cell proliferation was clearly observed within the time scale of our transcriptome data, we investigated whether PEP1-induced TFs suppressed cell cycle regulators. To address this, we searched for a connection between 344 PEP1-induced TFs and 388 cell cycle-related DEGs from Cluster 9 using the STRING database. We maintained the interaction parameter as “high confidence” (0.700) to avoid the large volume of noise. This in silico analysis revealed a large network made of 330 genes with 680 edges, which includes 2 subnetworks (Fig. 2A; Supplementary Table S10), one with a PEP1-induced TF cluster (Supplementary Fig. S5A) and the other with a distinct cell cycle gene cluster (Fig. 2A). Interestingly, these 2 subnetworks were connected via DOF AFFECTING GERMINATION 1 (DAG1/DOF3.7), PHY-INTERACTING FACTOR 1 (PIF1), and ABA INSENSITIVE 5 (ABI5), which were then connected to the most connected TFs in the PEP1-induced TF cluster.

In Arabidopsis, STZ, a C2H2 zinc finger (ZF) TF, has been reported to be involved in the multiple stress response pathways (Sakamoto et al. 2004; Mittler et al. 2006; Xie et al. 2012; Hoang et al. 2020; Feng et al. 2023). STZ was originally identified as a strong transcriptional repressor that is significantly affected by abiotic stressors, particularly salinity, cold, and drought (Lippuner et al. 1996; Sakamoto et al. 2000, 2004). Among the genes encoding C2H2 ZF TFs, *AZF3* and *ZAT6* were closely related to *STZ* (Supplementary Fig. S5C; Xie et al. 2019). Our transcriptome data showed that the expression levels of *AZF3*, *STZ*, and *ZAT6* rapidly increased under PEP1 exposure and then remained high (Fig. 2B). A quantitative real-time PCR (RT-qPCR) assay using root samples indicated that only 1 h of PEP1 treatment triggered the transcription of *STZ*, *AZF3*, and *ZAT6* (Fig. 2C).

Next, we examined the spatial expression patterns of *STZ*, *AZF3,* and *ZAT6* in 5-d-old Col-0 seedling roots introduced with *pSTZ::eGFPGUS*, *pAZF3::eGFPGUS*, and *pZAT6::eGFPGUS*. Previous cell-type-specific root map data (Brady et al. 2007; Zhang et al. 2019) predicted that these 3 ZF TFs are mainly expressed in cambium and phloem companion cells in the root differentiation zone under unperturbed conditions (Supplementary Fig. S5D). Consistent with this prediction, eGFP expression in *pSTZ::eGFPGUS* and *pZAT6::eGFPGUS* was observed within the stele of the differentiation zone, which was increased by PEP1 (Supplementary Fig. S5, E and F). However, no eGFP expression in *pSTZ::eGFPGUS* or *pZAT6::eGFPGUS* was detected in the root meristems without PEP1 treatment, and GUS staining of *pSTZ::eGFPGUS* lines showed little expression in the columella (Fig. 2, D and E; Supplementary Fig. S6, A and B). In contrast, in seedling roots exposed to PEP1 for 24 h, very high expression of *pSTZ::eGFPGUS* and *pZAT6::eGFPGUS* was observed throughout the meristems and in the stele of the elongation and differentiation zones (Fig. 2, D and E; Supplementary Figs. S5, E and F and S6, A and B). We further tracked their expression by GUS staining in time course after PEP1 treatment (Supplementary Fig. S7). Induction of *STZ* and *ZAT6* in the root stele and meristems started only 1 h post-PEP1 treatment and became stronger (Supplementary Fig. S7A). However, the expression of *ZAT6* was much lower than that of *STZ* (Supplementary Fig. S7B), which was consistent with RT-qPCR results (Fig. 2C). Similar to *STZ* and *ZAT6*, the expression of *eGFPGUS* was increased in the meristems of the transcriptional reporter lines of *AZF3* after PEP1 treatment (Fig. 2F; Supplementary Figs. S5G, S6C, and S7C). *pAZF3::eGFPGUS* was weakly expressed in the cortex of the meristem without PEP1 treatment. PEP1 increased *AZF3* expression in the cortex and inner layers of the meristem, elongation, and differentiation zones. In translational fusion lines (*pSTZ::STZ-cGFP* and *pZAT6::ZAT6-cGFP*), we detected STZ and ZAT6 proteins in root meristems after treatment with PEP1 (Fig. 2, G and H; Supplementary Fig. S6, D and E). However, their signals were weak and undetectable in the stele where PEP1 strongly induced transcriptional fusion GFPGUS. Furthermore, we could not find translational fusion lines for *AZF3* (*pAZF3::AZF3-cGFP*) with GFP signals. Nevertheless, the protein localization domains significantly overlapped between STZ and ZAT6, and ZAT6 appeared to be more enriched in the stem cell niche than STZ (Fig. 2, G and H).

Our in silico network of PEP1-responsive genes indicated that STZ was the 4th most connected TF (Supplementary Table S9). Constitutive expression of *STZ* or *ZAT6* has been shown to confer the suppression of shoot and root growth in Arabidopsis seedlings (Sakamoto et al. 2004; Shi et al. 2014), which is similar to the phenotype of seedlings exposed to PEP1. Furthermore, DAP-seq data predicted that 170 of the 388 genes in Cluster 9 suppressed by PEP1 were direct targets of STZ (O’Malley et al. 2016), which was the highest among the top 5 most connected TFs in the in silico network (Supplementary Table S11). These results imply that *STZ* and its close homologs (hereafter referred to as STZL) may serve as a nexus between PEP1-mediated stress signaling and the reprogramming of root development.

### STZ controls root meristem activities as part of the PEP signaling cascade

PEPs are endogenous elicitors secreted in response to attacks by bacteria, fungi, and herbivores (Yamaguchi and Huffaker 2011; Bartels and Boller 2015). PEP1 to 6 are recognized by PEP1 RECEPTOR1 (PEPR1) and PEP1 RECEPTOR1 (PEPR2). To find whether the STZ is downstream of the PEP signaling cascade, we monitored the expression levels of *STZ* and *PEPR*s in the roots 1 h after PEP1 treatment in the *pepr1 pepr2* and *stz* mutant seedlings (Supplementary Fig. S8). Consistent with a previous report (Jing et al. 2019), short-term treatment (1 h) with 1 *µ*m PEP1 increased *PEPR1* and *PEPR2* levels by ∼12- and ∼30-fold, respectively, in the wild-type (WT) seedling root (Supplementary Fig. S8, A and B). In the *stz* mutant, *PEPR1* and *PEPR2* were highly induced by PEP1 (Supplementary Fig. S8, A and B). However, in the *pepr1 pepr2* double mutant, no statistical difference was observed in *STZ* expression levels between the mock and PEP1-treated plants (Supplementary Fig. S8C). These results confirm that PEP-mediated signal perception through PEPRs acts upstream of STZ regulatory networks.

**Figure 3.**
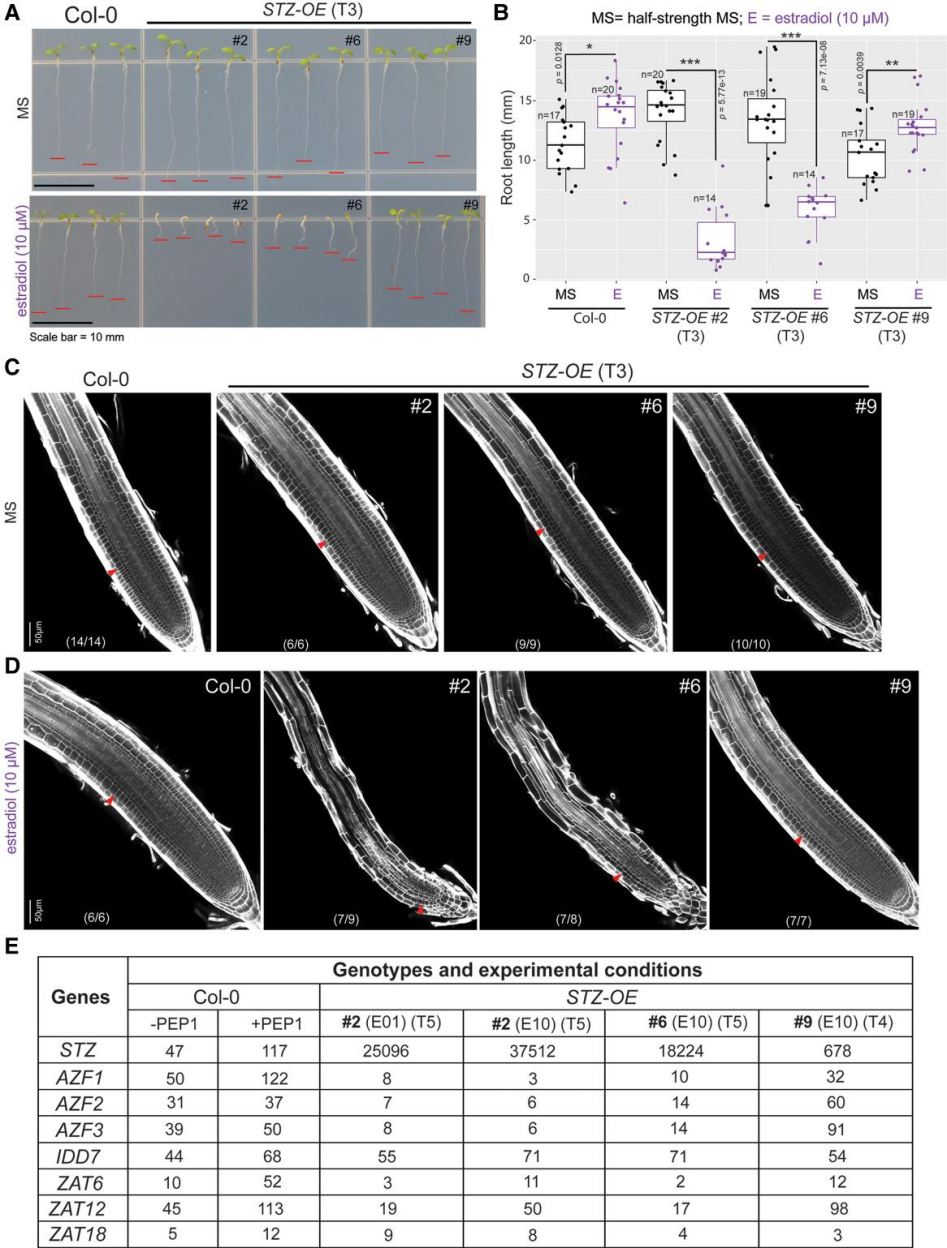
*STZ* suppresses root meristem activities in a dosage-dependent manner. **A)** The image of *STZ-OE* seedlings at 5 DAT on ½ MS with or without 10 *µ*m of estradiol. The seedlings with representative root lengths were arranged on an agar plate and photographed. Red lines indicate the root tips. Scale bar = 10 mm (applicable to both images in **A**). **B)** Root lengths of *STZ-OE* seedlings as shown in **A)**. Significant differences were determined by comparing seedlings treated with estradiol to those that were untreated using a Student's *t*-test (***P < 0.001, **P < 0.01, *P < 0.05). Boxes, the first and third quartiles split by median; whiskers, the range; points, individual data points; E, estradiol (10 *µ*m). **C and D)** Confocal microscopy images of *STZ-OE* seedling roots prepared as shown in **A and B)**. The red arrowhead marks the junction between the meristem and the transition zones. The numbers in parentheses in each panel indicate the number of samples with the presented phenotype among the total individuals analyzed. Scale bar = 50 *µ*m (applicable to all images within **C and D)**. **E)** The expression levels of *STZ* and *STZ* homologs in the meristem were determined using ddPCR. The numbers in the table denote the number of transcripts of each gene present in 10 ng of total RNA in the meristem. WT Col-0 seedlings without the PEP1 treatment (“-PEP1”) were used as the background. The PEP1 (1 *µ*m) and estradiol (E01, 1 *µ*m of estradiol; E10, 10 *µ*m of estradiol) treatment was performed for 24 h on seedlings at 4 DAT. *STZ-OE*, *STZ* overexpression line; DAT, days after transfer to the growth chamber.

We then investigated whether STZ overexpression alone can recapitulate the PEP1-mediated root growth response. To this end, we developed *STZ*-overexpressing transgenic lines using a well-established estrogen-inducible XVE system (Zuo et al. 2000; Siligato et al. 2016). We transformed Col-0 plants with the vector carrying *p35S::XVE>>pLexA::STZ* (*STZ-OE*) transgene. After selection for Basta resistance, the induction levels of *STZ* in T2 *STZ-OE* seedlings were estimated using RT-qPCR 24 h post-estradiol treatment (Supplementary Fig. S9A). We selected 3 *STZ-OE* lines (Lines #2, #6, and #9 with 3,883.3-, 730.7-, and 263.8-fold *STZ* expression, respectively, compared with Col-0 seedlings), which had very high, high, and moderate induction levels of *STZ* expression. When grown on half-strength Murashige and Skoog (½ MS) medium, the T2 and T3 generations of *STZ-OE* Lines #2, #6, and #9 displayed root growth comparable to that of Col-0 seedlings. However, when grown in 10 *µ*m estradiol, seedlings of Line #2 displayed the most severe reduction of root growth with pale yellow cotyledon phenotype (Fig. 3, A and B; Supplementary Fig. S9B). In contrast, Line #9 exhibited mild suppression of root growth in the estradiol medium (Supplementary Fig. S9B). Line #6 showed the root phenotype in-between Lines #2 and #9.

To understand the root growth suppression of *STZ-OE*, we imaged the root meristems of *STZ-OE* lines. Meristem cells were completely exhausted in T3 individuals of *STZ-OE* Line #2. By contrast, Line #9 maintained intact meristem cells after long-term *STZ* induction (Fig. 3, C and D; Supplementary Fig. S10). *STZ-OE* #6 retained a small size of meristem cells, again showing the in-between phenotype. Since the shoot growth of *STZ-OE* Line #2 was severely inhibited in response to estradiol, we investigated whether the root phenotype was an indirect effect of the shoot growth defect or the local action of highly expressed STZ in the root. To resolve this issue, we performed reciprocal grafting between STZ-OE #2 and WT Col-0. The rootstock of *STZ-OE* #2 grafted under the WT scion still exhibited exhaustion of the root meristem in response to estradiol (Supplementary Fig. S11), indicating that a high dose of STZ locally acted to suppress root meristem activity.

*STZ* levels in selected *STZ-OE* lines were unusually high compared with those induced by PEP1. This raised a question of whether the differential expression of other *STZ* homologs in *STZ-OE* lines is primarily responsible for suppressing the root meristem. To address this, we measured *STZ* and its homologs in the root meristems dissected from *STZ-OE* lines in response to estradiol and WT Col-0 treated with PEP1 (Fig. 3E; Supplementary Fig. S12). Digital droplet PCR (ddPCR) of cDNAs from total RNAs of root meristems indicated that *STZ-OE* lines required much higher *STZ* transcripts to trigger the suppression of root meristem activity. Interestingly, the expression of several STZ homologs was suppressed in *STZ-OE* #2 and #6 but not in *STZ-OE* #9. This suggests that complicated negative feedback regulation exists between STZ and its homologs. Nevertheless, the ddPCR data collectively rule out the possible primary involvement of other STZ homologs in root meristem suppression.

### STZ controls stem cell niche and xylem domains in the root meristem

Exhaustion of the root meristem in *STZ-OE* #2 seedlings can occur when the stem cell niche in the root meristem is not maintained. To further analyze this aspect, we transferred *STZ-OE* T3 seedling lines germinated and grown on ½ MS medium with estradiol for 7 d to a ½ MS medium without estradiol and inspected their root growth recovery over 8 d after the transfer. *STZ-OE* #9 seedlings had fully restored root growth, similar to that in WT seedlings; however, *STZ-OE* #2 and #6 seedlings did not (Supplementary Fig. S13). These results suggest that a very high dose of STZ irreversibly disrupts the stem cell population in the root meristem. In contrast to *STZ-OE* #2 and #6 seedlings, the WT seedlings, which germinated and grew on ½ MS medium with PEP1 for 6 d, fully restored their root growth when placed back in a medium without PEP1 (Supplementary Fig. S14).

**Figure 4.**
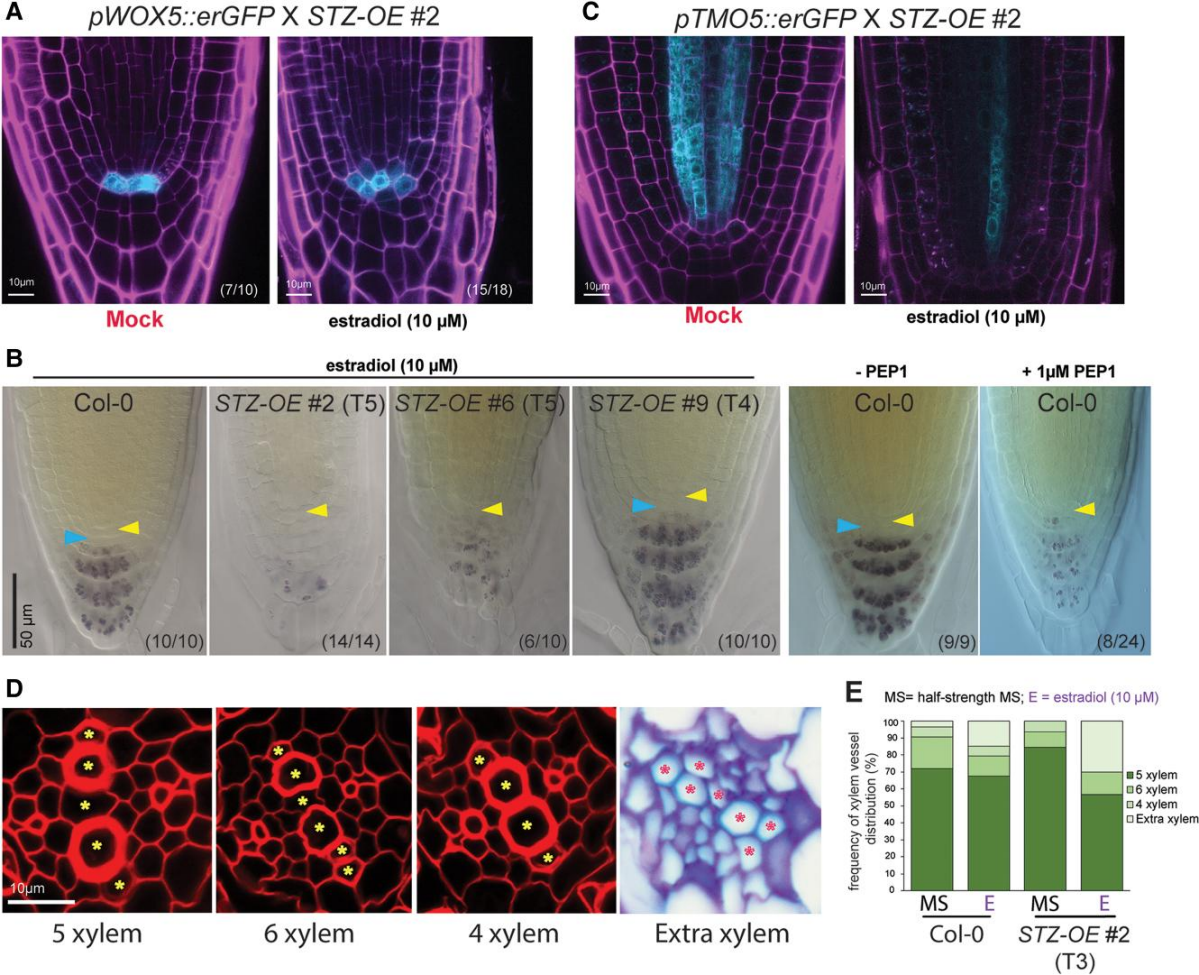
A high dosage of *STZ* perturbs the stem cell niche and xylem patterning. **A)** Analysis of *pWOX5::erGFP*, a QC marker, in the presence of a high dose of *STZ*. Five DAT F2 seedlings between *pWOX5::erGFP* and *STZ-OE* #2, showing a stunted root phenotype after estradiol treatment, were compared with seedlings without estradiol. **B)** Lugol's staining of the estradiol (10 *µ*m)-treated root tips of Col-0, *STZ-OE* #2, *STZ-OE* #6, and *STZ-OE* #9 seedlings (from left to right) and the root tips of Col-0 seedlings with and without PEP1 (1 *µ*m) treatment. Seedlings at 3 DAT on ½ MS medium were transferred and grown on ½ MS medium supplemented with estradiol or PEP1 for 48 h before Lugol's staining. The yellow and blue arrowheads indicate the QC and columella stem cell (CSC) layers, respectively. Scale bar = 50 *µ*m (applicable to all images within **B)**. **C)** Analysis of *pTMO5::erGFP*, a xylem precursor marker, in the presence of a high dose of *STZ*. F2 seedlings at 5 DAT between *pTMO5::erGFP* and *STZ-OE* #2, showing a stunted root phenotype after estradiol treatment (9 individuals), were compared with seedlings without estradiol (4 individuals). **D)** Representative images of 4 typical xylem arrangements found in the roots of WT Col-0 and *STZ-OE* #2 seedlings at 5 DAT; the “5 xylem,” “6 xylem,” “4 xylem,” and “extra xylem” phenotypes. Yellow and red asterisks indicate the differentiated xylem vessels. Scale bar = 10 *µ*m (applicable to all images within **D)**. **E)** Quantification of the xylem differentiation phenotypes in 5 DAT WT Col-0 and *STZ-OE* #2 seedlings grown on ½ MS medium supplemented with 10 *µ*m estradiol. The seedlings grown on ½ MS medium without estradiol (MS) were used as a control. Section images and detailed xylem vessel distribution frequencies are presented in Supplementary Fig. S17 and Supplementary Table S12, respectively. Sample number (*n*) = 30 to 34. Mock treatment = seedlings grown on ½ MS medium supplemented with an equal volume of ethanol as estradiol (10 *µ*m); E, estradiol (10 *µ*m) = seedlings grown on ½ MS medium supplemented with 10 *µ*m estradiol. The numbers in parentheses in each panel indicate the number of samples with phenotypes similar to the representative image among all independent root samples analyzed. *STZ-OE*, *STZ* overexpression line. GFP: cyan; PI staining: magenta. DAT, days after transfer to the growth chamber.

To further investigate the status of the root stem cell niche in *STZ-OE* #2 seedlings, we introduced a marker of quiescent center (QC), *pWOX5::erGFP* (Sarkar et al. 2007), into *STZ-OE* #2 seedlings by genetic crossing, and monitored the GFP expression patterns in 5-d-old F2 generation seedlings grown on estradiol-containing medium. *WOX5* domain frequently expanded beyond the original QC position (Fig. 4A; Supplementary Fig. S15), indicating disruption of QC quiescence. When the QC loses its function, the organization of the columella cell layers is frequently affected. Thus, we performed Lugol's staining on *STZ-OE* seedling lines and compared the results with those of the Col-0 lines treated with PEP1 (Fig. 4B; Supplementary Fig. S16). Interestingly, starch granules disappeared as the STZ dosage increased in the *STZ-OE* lines. This phenomenon was also observed in Col-0 seedlings treated with PEP1, indicating that STZ is the primary mediator of changes in the root stem niche in response to PEP1.

PEP1 promotes xylem differentiation in the WT roots (Dhar et al. 2021). We investigated whether *STZ-OE* seedlings mimicked the PEP1-mediated xylem phenotype. The expression level of *pTMO5::erGFP* (Lee et al. 2006), a marker for xylem precursor cells, introduced into *STZ-OE* Line #2 seedlings, drastically decreased (Fig. 4C). We also scored xylem differentiation patterns in the 5-d-old roots of T3 *STZ-OE* #2 seedlings grown on 10 *µ*m estradiol medium in comparison with WT seedlings (Fig. 4, D and E; Supplementary Fig. S12; Zhou et al. 2013; Dhar et al. 2021; Seo and Lee 2021). In the medium without estradiol, WT seedling roots showed “5 xylem cells” as the most prevalent type (∼72%), followed by the “6 xylem cells” (∼19%), “4 xylem cells” (∼6%), and “extra xylem” (∼3%) types (Fig. 4E; Supplementary Fig. S17A and Table S12). The *STZ-OE* #2 seedlings showed xylem patterns similar to that of WT seedlings with the presence of 84% of the “5 xylem cells” type, followed by 9% of the “6 xylem cells” and 6% of “4 xylem cells” types (Fig. 4E; Supplementary Fig. S17B and Table S12). However, estradiol-treated *STZ-OE* #2 seedlings displayed a significant increase in “extra xylem” vessel differentiation compared with the WT seedlings grown on 10 *µ*m estradiol plates (∼15% in WT vs. ∼30% in *STZ-OE*#2) (Fig. 4E; Supplementary Fig. S17, C and D and Table S12). These *STZ-OE* phenotypes mimic the PEP1-influenced WT phenotypes, supporting the crucial role of STZ in PEP-mediated reprogramming of root development (Dhar et al. 2021).

### Identification of downstream regulators of the PEP-STZ pathway

**Figure 5.**
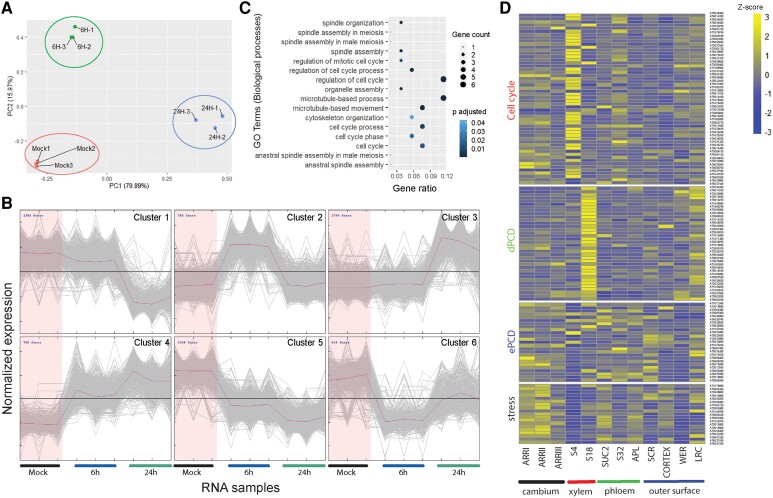
*STZ* transcriptionally regulates a group of cell cycle, dPCD, ePCD, and stress regulatory genes downstream of PEP1 signaling. **A)** PCA of the 9 *STZ-OE* RNA-seq samples based on the top 5,000 most variable genes. We used RNA samples from 2 time points (6 and 24 h) after *STZ* induction and 1 set of mock samples. The sample information and raw data are presented in Supplementary Tables S13 and S14, respectively. **B)** K-means clustering of 8,435 DEGs (Supplementary Table S15). A list of the genes in each cluster is presented in Supplementary Table S16. **C)** GO enrichment (biological processes) analysis (FDR < 0.05) of common cell cycle target DEGs that were suppressed by both PEP1 treatment and STZ overexpression; data are presented in Supplementary Tables S17 and S18. **D)** Root expression patterns of the cell cycle, dPCD, ePCD, and stress regulatory TFs downstream of STZ (Supplementary Table S19). The abbreviated information for the cell-type-specific markers is presented in Supplementary Table S28. The color scale bars denote the *Z*-score as obtained through the “scale” function while plotting using the “pheatmap” package in R. *STZ-OE*, *STZ* overexpression line.

To understand how *STZ* transcriptionally modulates downstream signaling cascades, we generated RNA-seq data for the roots of T3 STZ-OE #2 seedlings sampled at 6 and 24 h post-*STZ* induction (Supplementary Fig. S18A). The RPKM values of all mapped transcripts were obtained for 9 samples with over 500 million high-quality paired-end reads (Supplementary Tables S13 and S14). PCA with the top 5,000 variable genes revealed sample integrity between replicates (Fig. 5A). At a 1.5-fold cutoff, we identified 8,435 DEGs compared with the mock samples (Supplementary Fig. S18, B and C and Table S15). Although STZ acts as a transcriptional repressor, more than 50% of the DEGs were upregulated after STZ induction (Supplementary Fig. S18C). K-means clustering of these DEGs revealed 6 clusters (Fig. 5B), with Clusters 1, 5, and 6 showing suppression by STZ and Clusters 2, 3, and 4 showing rapid to late induction by STZ (Fig. 5B; Supplementary Table S16).

Since proliferative cell division in the root meristem was suppressed by both *STZ* overexpression and PEP1 treatment, we searched for cell cycle genes by overlaying genes suppressed by PEP1 and STZ (Clusters 1, 5, and 6 for *STZ-OE* data and Cluster 9 for PEP1 data; Supplementary Tables S4 and S16). Sixty-one overlapping genes (Supplementary Table S17 and Fig. S18D) belonged to the GO terms, “regulation of cell cycle” (GO:0051726) and “regulation of mitotic cell cycle” (GO:00007346) (Fig. 5C; Supplementary Table S18). Interestingly, these 61 genes were significantly enriched in developing xylem precursor (S4) cells (Fig. 5D; Supplementary Table S19).

The T3 *STZ-OE* #2 seedlings exhibited early and ectopic xylem differentiation (Fig. 4, D and E; Supplementary Fig. S17), similar to seedlings treated with PEP1 (Dhar et al. 2021). Therefore, we investigated whether STZ increased developmentally induced PCD (dPCD) genes. We compared the DEGs from the STZ-induced clusters with a core set of 98 conserved dPCD marker genes that were previously identified in the context of tracheary elements and lateral root cap differentiation (Lee et al. 2006; Olvera-Carrillo et al. 2015), and identified 36 shared dPCD genes (Supplementary Fig. S18E and Table S20). We also identified 26 environmentally induced PCD (ePCD) marker genes upregulated by STZ (Supplementary Fig. S18F and Table S19), out of 84 known ePCD genes upregulated by several shared biotic, genotoxic, and osmotic stressors (Olvera-Carrillo et al. 2015). The expression profiles of dPCD (36/98) and ePCD (26/84) genes in our transcriptome data are shown in Supplementary Fig. S18, G and H. In the cell-type-specific root expression data, STZ downstream dPCD genes tended to show enriched expression in the maturing xylem (S18), whereas ePCD genes were sporadically expressed throughout various cell layers (Fig. 5D; Supplementary Table S19). These findings suggest that STZ preferentially controls xylem-enriched cell cycle- and differentiation-related genes in roots.

Furthermore, among the 344 TFs induced by PEP1 treatment in our time-course transcriptome data, 190 were found as DEGs in the *STZ-OE* data, and 66 were predicted to be direct targets of STZ (O’Malley et al. 2016; Supplementary Table S21). Among 190 TFs regulated by STZ in the PEP1-triggered GRN, 19 were directly associated with STZ in the STRING database with functional annotations in stress responses (Supplementary Table S22). Interestingly, these stress regulators were highly enriched in the cambium (ARRI), developing cambium (ARRII), companion cells (SUC2), phloem (APL), and cortex layers in the root (Fig. 5D). STZ direct targets included TFs involved in the vascular development such as MONOPTEROS (MP; Przemeck et al. 1996), VASCULAR-RELATED NAC DOMAIN 6 (VND6; Kubo et al. 2005), OBF BINDING PROTEIN 4 (OBP4; Miyashima et al. 2019), TARGET OF MONOPTEROS 6 (TMO6; Schlereth et al. 2010), and HIGH CAMBIAL ACTIVITY2 (HCA2; Guo et al. 2009; Miyashima et al. 2019), further supporting the notion that STZ is a core TF in the PEP1-triggered GRN, which modulates stress responses and meristem/vascular development.

### STZ regulates stress response and root growth with a discrete threshold

**Figure 6.**
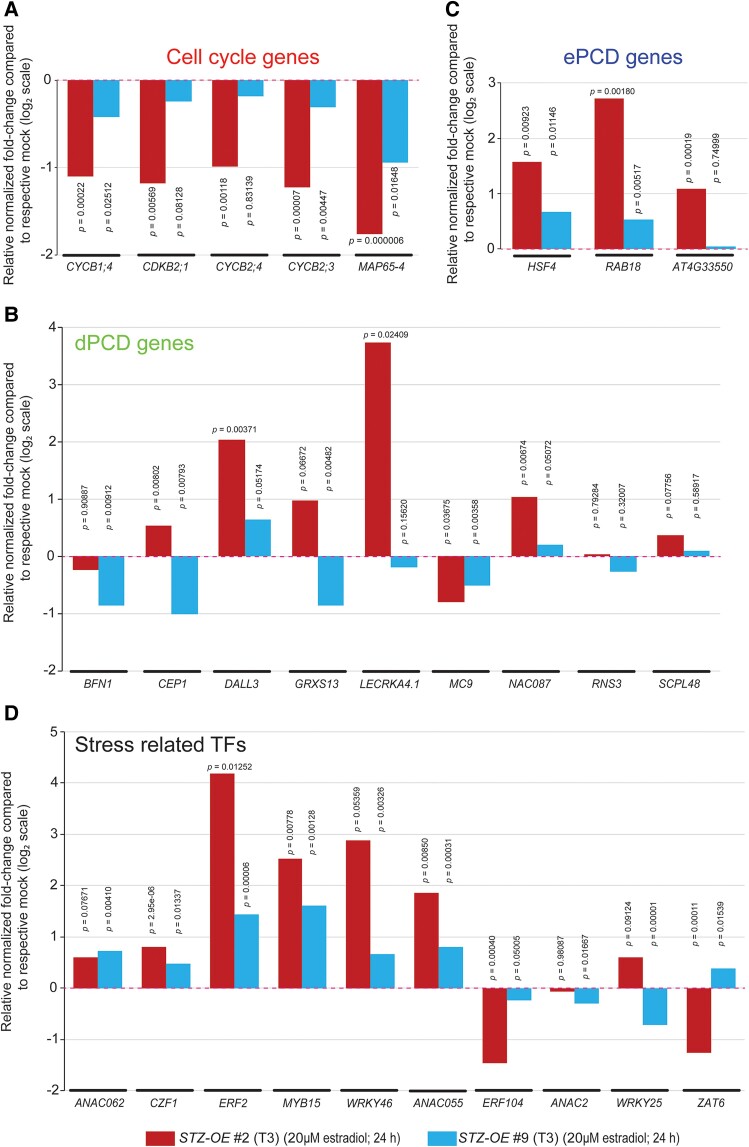
Dosage-dependent regulation of cell cycle, dPCD, ePCD, and stress regulators by STZ. **A to D)** Expression levels of the selected cell cycle **A)**, dPCD **B)**, ePCD **C)**, and stress regulatory TFs **D)** analyzed by RT-qPCR in the roots of *STZ-OE* #2 and #9 seedlings. The 4 DAT seedlings of STZ-OE #2 and #9 were treated with 20 *µ*m estradiol or mock (an equal volume of ethanol used for dissolving estradiol) for 24 h, and the bottom half of their roots were dissected for total RNA isolation. *UBQ10* was used as an internal control, and the normalized fold change was determined in the root samples treated with 20 *µ*m estradiol for 24 h when compared with mock. The normalized expression values are presented as the ±SEM from 3 technical replicates in Supplementary Table S23. The bar graph represents the log_2_ |normalized fold change| from 3 technical replicates. Pink dotted lines over the bar graphs signify the normalized transcript levels of each respective gene at mock treatment (mock = 1). The statistical significance of differences was determined using a Student's *t*-test compared with mock, and the *P* values are presented. *STZ-OE*, *STZ* overexpression line; DAT, days after transfer to the growth chamber.

Given the significant difference in root growth and stem cell population maintenance between *STZ-OE* #2 and #9 seedlings and the intermediary phenotype of #6 (Figs. 3 and 4; Supplementary Figs. S9, S10, and S13), STZ seemed to regulate downstream processes in a dose-dependent manner. To explore this aspect further, we measured the expression of selected cell cycle, dPCD, and ePCD genes in the roots of T3 *STZ-OE* #2 and #9 seedlings using RT-qPCR (Fig. 6, A to C). As expected, the cell cycle genes exhibited more than 50% suppression on average after estradiol treatment for 24 h in the *STZ-OE* #2 roots compared with the mock treatment, whereas *STZ-OE* #9 seedlings maintained overall transcript levels of approximately 80% of the mock treatment (Fig. 6A; Supplementary Table S23). We also monitored the expression of well-characterized dPCD regulators, which include *ARABIDOPSIS NAC DOMAIN CONTAINING PROTEIN 87* (*ANAC087*), *SERINE CARBOXYPEPTIDASE-LIKE 48* (*SCPL48*), *GLUTAREDOXIN 13* (*GRXS13*), *RIBONUCLEASE 3* (*RNS3*), *BIFUNCTIONAL NUCLEASE 1* (*BFN1*), *DAD1-LIKE LIPASE 3* (*DALL3*), *LECTIN RECEPTOR KINASE A4.1* (*LECRKA4.1*), *METACASPASE 9* (*MC9*), and *CYSTEINE ENDOPEPTIDASE 1* (*CEP1*) (Fig. 6B; Supplementary Table S23). In Arabidopsis, ANAC046 and ANAC087, 2 NAC domain TFs in xylem vessels, have been shown to mediate PCD via BFN1 and RNS3 (Olvera-Carrillo et al. 2015; Fujimoto et al. 2018; Huysmans et al. 2018). In general, the results indicated higher induction of dPCD genes in the roots of *STZ-OE* #2 seedlings than in that of #9 seedlings (Fig. 6B; Supplementary Table S23). Similar patterns were observed for 3 ePCD marker genes: *HEAT SHOCK FACTOR 4* (*HSF4*), *RESPONSIVE TO ABA 18* (*RAB18*), and *BIFUNCTIONAL INHIBITOR/LIPID-TRANSFER PROTEIN* (AT4G33550) (Fig. 6C; Supplementary Table S23). These analyses indicated that a very high dose of STZ in the root influences the cell cycle and differentiation gene cascades.

Among the 19 stress response TFs downstream of both PEP1 and *STZ-OE* #2 (Fig. 5D; Supplementary Fig. S18I and Table S22), the expression of 8 well-known stress regulators and 2 direct targets of STZ that belong to PEP1-induced TFs (O’Malley et al. 2016; Supplementary Fig. S18I and Table S24) was monitored in the roots of T3 *STZ-OE* #2 and #9 seedlings using RT-qPCR (Fig. 6D; Supplementary Table S23). *WRKY46*, *MYB DOMAIN PROTEIN 15* (*MYB15*), *ANAC055*, and *ETHYLENE RESPONSE FACTOR-2* (*ERF2*) were highly upregulated (>4-fold compared with the mock treatment) in *STZ-OE* #2 seedlings (Fig. 6D; Supplementary Table S23). Although *STZ-OE* #9 seedlings did not show root growth suppression upon STZ induction (Fig. 3, A and B), they displayed a significant increase (1.5- to 3-fold compared with the mock treatment) in RNA levels for these stress regulators. The expression of *CZF1* and *ANAC062*, which are the direct targets of STZ (O’Malley et al. 2016), was also increased in both lines (Fig. 6D; Supplementary Table S23). Furthermore, *ZAT6*, a close STZ homolog, was differentially regulated by STZ doses, that is, it was suppressed in Line #2 but activated in Line #9 (Fig. 6D; Supplementary Table S23). These observations suggest that STZ alters the magnitude of stress resistance in roots, depending on the *STZ* level. However, the basal level of STZ for inducing stress resistance seems to be lower than the level required for growth suppression, as *STZ-OE* #9 seedlings activated stress resistance genes without suppressing growth-related genes. These discrete thresholds of STZ required for stress resistance and growth suppression might enable plants to determine the growth–defense tradeoff.

### Engineering STZ doses for making stress-resilient plants

**Figure 7.**
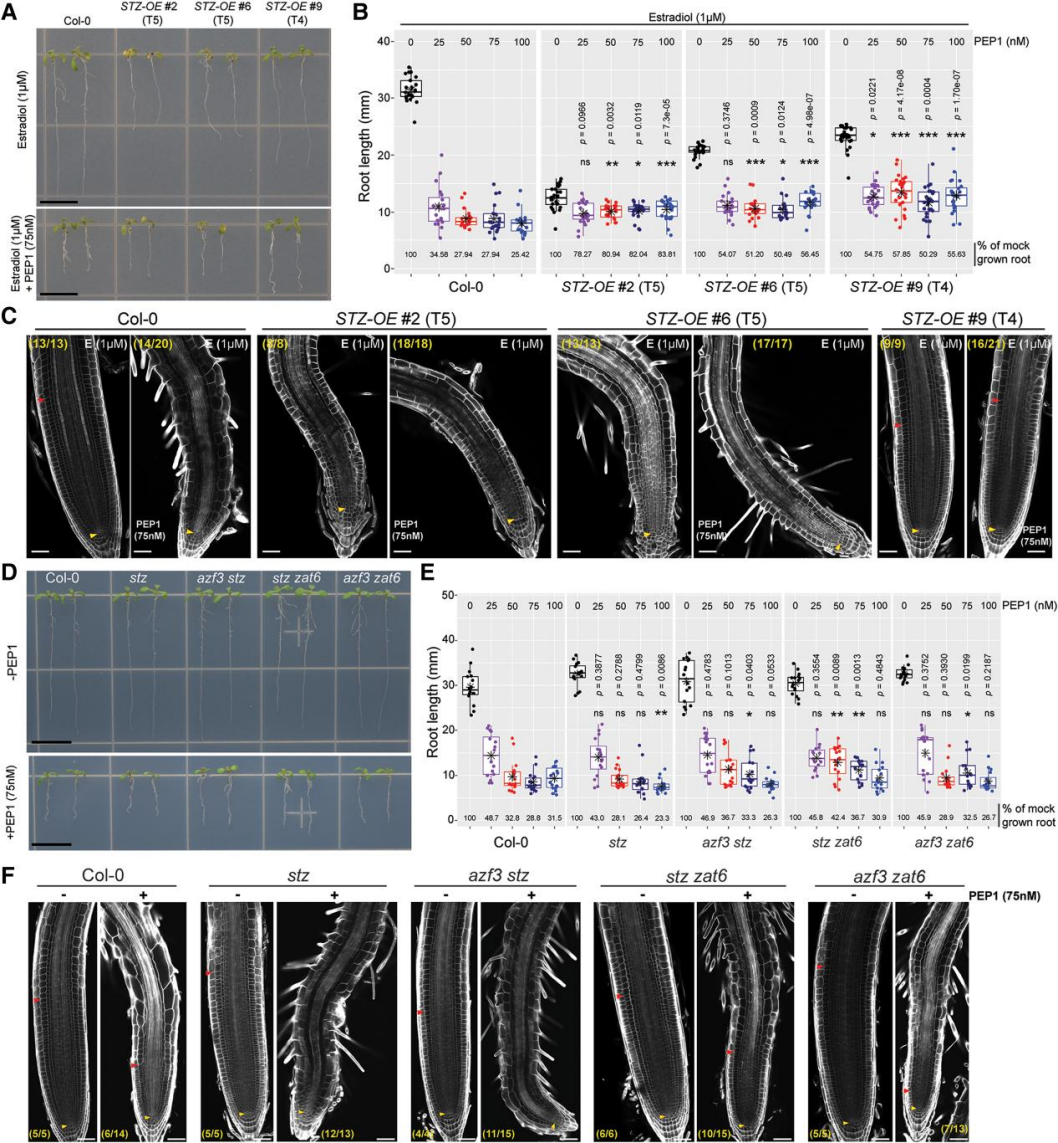
STZ-dependent engineering of stress-responsive root growth. **A)** Photographs of Col-0 and *STZ-OE* seedlings at 7 DAT grown in the presence of 1 *µ*m estradiol with or without 75 nm PEP1 for 4 d. Seedlings at 3 DAT germinated and grown on ½ MS medium were transferred to ½ MS medium containing 1 *µ*m estradiol with or without 75 nm PEP1. The seedlings with representative root lengths were arranged on an agar plate and photographed. The *STZ-OE* seedlings used in this experiment were T4 or T5 generations of lines #2, #6, and #9. Scale bar = 10 mm (applicable to both images under **A)**. **B)** Box plots of root lengths of 7 DAT Col-0 and *STZ-OE* seedlings treated with estradiol (1 *µ*m) and PEP1 (25, 50, 75, and 100 nm) for 4 d as shown in **A)**. Significant differences were determined by comparing WT Col-0 and *STZ-OE* seedlings grown under the same treatment conditions using a 1-way Student's *t*-test (***P < 0.001, **P < 0.01, *P < 0.05; ns, nonsignificant). The quantitative data and other biological replicate data are presented in Supplementary Table S25 (*n* = 18 to 26). Boxes, the first and third quartiles split by median; whiskers, the data range; points, individual data points; asterisks within the boxplot, average. **C)** Confocal microscopy of root meristems of Col-0 and *STZ-OE* seedlings under the conditions described in **A)**. **D)** Photographs of 7 DAT Col-0 and *stzl* mutant seedlings grown with or without 75 nm PEP1. Seedlings at 3 DAT that germinated and grew on ½ MS medium were transferred to ½ MS medium with or without 75 nm PEP1 for 4 d. The seedlings with representative root lengths were arranged on an agar plate and photographed. Scale bar = 10 mm (applicable to both images under **D)**. **E)** Box plots of root lengths of Col-0 and *st*z*l* mutant seedlings at 7 DAT treated with PEP1 (25, 50, 75, and 100 nm) for 4 d, as shown in **D)**. Significant differences were determined by comparing Col-0 seedlings to *stzl* mutants grown under the same treatment conditions using a 1-way Student's *t*-test (**P < 0.01, *P < 0.05; ns, nonsignificant). The quantitative and other biological replicate data are presented in Supplementary Table S25 (*n* = 15 to 18). Boxes, the first and third quartiles split by median; whiskers, the data range; points, individual data points; asterisks within the boxplot, average. **F)** Confocal microscopy of root meristems of Col-0 and *stzl* mutant seedlings under the conditions described in **D)**. Yellow and red arrowheads in **C and F)** indicate the QC and meristem/transition zone boundaries, respectively. The numbered ratio in parentheses indicates samples with similar results to the total independent root samples analyzed. Scale bar = 30 *µ*m (applicable to all images under **C and F)**. *STZ-OE*, *STZ* overexpression line; DAT, days after transfer to the growth chamber.

Our study suggests that PEP-mediated signaling employs *STZ* to control stress resistance and growth in a dose-dependent manner. It still remains to answer whether the STZ doses would benefit root growth under the influence of stress-triggering signals (Fig. 7, A and B; Supplementary Fig. S19, A and B). We monitored the root growth in T4 or T5 *STZ-OE* #2, #6, and #9 seedlings, after the mock treatment (1 or 10 *µ*m of estradiol) and various doses of PEP1 (25, 50, 75, and 100 nm with 1 or 10 *µ*m of estradiol). Root growth suppression occurred at both estradiol concentrations following the magnitude of STZ induction: *STZ-OE* #2, #6, and #9 (Fig. 7, A and B; Supplementary Fig. S19, A and B and Table S25). When *STZ-OE* seedlings were grown under different doses of PEP1 together with estradiol, their root growth suppression was less than that of WT seedlings, that is, the higher the *STZ* concentration, the higher the resistance to PEP1. Following this trend, *STZ-OE* #6 and #9 seedlings had longer roots than WT seedlings, depending on the doses of PEP1 and estradiol (Fig. 7B; Supplementary Fig. S19B). Furthermore, when we analyzed the meristems of the *STZ-OE* lines in the mock and PEP1 treatments, the meristems of *STZ-OE* #9 roots were more intact than those of the WT roots (Fig. 7C; Supplementary Figs. S19C and S20A). We further explored this aspect by inducing *STZ* 1 d before exposing the seedlings to PEP1 (Supplementary Fig. S21, A and B). In this priming experiment, *STZ-OE* #6 seedlings showed significantly greater root growth after PEP1 treatment than the WT. These findings suggest the potential for engineering STZ doses to improve root growth performance under stresses.

Finally, using CRISPR/Cas9 knockout mutant combinations of STZLs, we explored the effects of in vivo STZL (STZ, ZAT6, and AZF3) levels on PEP1-mediated root growth suppression. A versatile multiplex CRISPR/Cas9 assembly system (Ursache et al. 2021; Supplementary Fig. S22) was employed to introduce multiple Cas9-gRNAs for *AZF3* and *ZAT6* simultaneously into the *stz* T-DNA insertion mutant (SALK_054092), and Col-0, and *azf3 stz*, *stz zat6*, and *azf3 zat6* double mutants were successfully identified (Supplementary Fig. S23, A to C). The root growth sensitivity of Col-0, *stz*, *azf3 stz*, *stz zat6*, and *azf3 zat6* seedlings to PEP1 was measured in seedlings that were germinated and grown on ½ MS medium for 3 d and then grown for another 4 d on ½ MS medium with or without variable doss of PEP1 (Fig. 7, D and E; Supplementary Table S25). Consistent with the contribution of STZ to PEP1 resistance in the analysis of *STZ-OE* lines, the *stz* seedlings displayed hypersensitive root growth suppression compared with Col-0 seedlings at higher doses of PEP1 (75 and 100 nm). Such hypersensitive root growth suppression was not observed in *azf3 stz* and *azf3 zat6* seedlings (Fig. 7E; Supplementary Table S25). Interestingly, *stz zat6* seedlings showed less sensitive root growth suppression than Col-0 seedlings when exposed to 50 and 75 nm of PEP1 (Fig. 7E; Supplementary Table S25). Consistent with the root growth response, the *stz zat6* seedlings maintained the meristem to a level comparable to that of Col-0 seedlings, whereas most *stz* seedlings displayed complete exhaustion of the meristem following PEP1 exposure (Fig. 7F; Supplementary Fig. S20B). These observations collectively suggest that *STZ* is a core regulator of the growth–defense tradeoff while interacting with *STZ* homologs in a rather complicated manner.

## Discussion

Plant roots are constantly exposed to complex environmental stressors. These stressors activate the endogenous danger signals to act on the growth–defense tradeoff (Huot et al. 2014; Figueroa-Macias et al. 2021). PEP, a danger signal activated (Bartels and Boller 2015) by various biotic and abiotic stressors, triggers defense responses while suppressing apical root growth and inducing the differentiation of root hairs and vascular tissues (Poncini et al. 2017; Jing et al. 2019; Dhar et al. 2021). However, the mechanisms by which PEP1 transcriptionally manipulates root developmental pathways and defense responses remain elusive (Bartels and Boller 2015). This study aimed to understand how stress perception affects developmental outcomes by combining genome-wide expression analysis, gene network-based functional inferences, various gene perturbations, and detailed phenotypic analyses.

The meristem is the center that governs organ growth; thus, it is expected that PEP manipulates developmental regulatory programs in the root meristem to cope with environmental stressors. Indeed, our high-resolution root meristem-specific RNA-seq dataset in response to synthetic PEP1 revealed the rapid induction of a plethora of stress-responsive TFs in the meristem as early as 3 h post-PEP1 treatment. We hypothesized that some TFs were responsible for suppressing cell cycle genes in the meristem as a part of the growth–defense tradeoff (Figs. 1 and 2). We used a network based on information built into the STRING database as a proxy for candidate identification. Our search for connections between the downstream genes of PEP1 signaling, which belong to the cell cycle regulators and stress response TFs, indicated that *STZ* is one of the most highly connected TFs in the network with an indirect link to the cell cycle networks. The DAP-seq data are not implemented in the STRING database. Therefore, we visited these data and found that STZ may directly regulate more cell cycle genes in the meristem than any other highly connected TFs in this network.

STZ, AZF3, and ZAT6 are the most closely related members of the C2H2 zinc finger TF family. The expression dynamics of STZ, AZF3, and ZAT6 in response to PEP1 treatment further supported their potential role in regulating the cell cycle network, as inferred from our STRING database analysis. Their expression is normally absent in the root meristem but is rapidly induced in the root apical meristem with PEP1. STZ and ZAT6 proteins were distributed in the root stem cell niche following PEP1 treatment, indicating that PEP1-mediated reprogramming of root development may occur through STZ and its homologs (Fig. 3). Indeed, administering a very high dose of STZ in the root meristem using an estradiol-inducible transactivation system (*STZ-OE* Line #2) resulted in the (i) exhaustion of the stem cell niche, (ii) early and ectopic xylem vessel differentiation, and (iii) aberrant behaviors of the QC and columella (Fig. 4). These phenotypes were very similar to those of seedlings treated with PEP1 (Jing et al. 2019; Dhar et al. 2021; Okada et al. 2021). RNA-seq analyses further support the *STZ-OE* transgenic Line #2 phenotypes. STZ-suppressed clusters were enriched with cell cycle genes, whereas the STZ-activated clusters were enriched with dPCD-/ePCD-associated regulators and executors (Olvera-Carrillo et al. 2015; Figs. 5 and 6). Most dPCD-associated genes were enriched in the maturing xylem, suggesting the role of STZ in the differentiation of xylem vessels, where STZ is expressed in the roots without PEP1 treatment.

Fine-tuning the growth–defense tradeoff is critical for plant sustainability in constantly changing environments (Karasov et al. 2017), particularly in the root system. Once the stress-responsive network is activated, growth is suppressed. However, the degree of growth suppression depends on multiple factors, including stress intensities. We found that STZ regulates the stress response and growth-related genes at different levels (Fig. 6). Among the estradiol-inducible *STZ-OE* lines, #9 seedlings with a moderate *STZ* induction level showed root and meristem growth comparable to that of the WT seedlings even after *STZ* induction; however, it exhibited activation of the key stress regulators, *ANAC055*, *MYB15*, *ERF2*, *ZAT6*, *CZF1*, and *ANAC062* (Fig. 6). Contrary to STZ-OE #9 seedlings, *STZ-OE* Line #2 seedlings exhibited both stress gene induction and root growth suppression (Figs. 6 and 7). Consistent with the induction of key stress regulators, the roots of *STZ-OE* #9 seedlings exposed to PEP1 grew longer than those of Col-0 seedlings without compromising the meristem (Fig. 7). Aligned with this result, the *stz* mutant showed stronger root growth suppression after PEP1 treatment than the WT seedlings. These data suggest that plants might control doses of STZ depending on the severity of stresses to decide discretely whether to increase a mild level of stress resistance without compromising growth or solely invest in stress resistance without diverting energy to the growth program.

**Figure 8.**
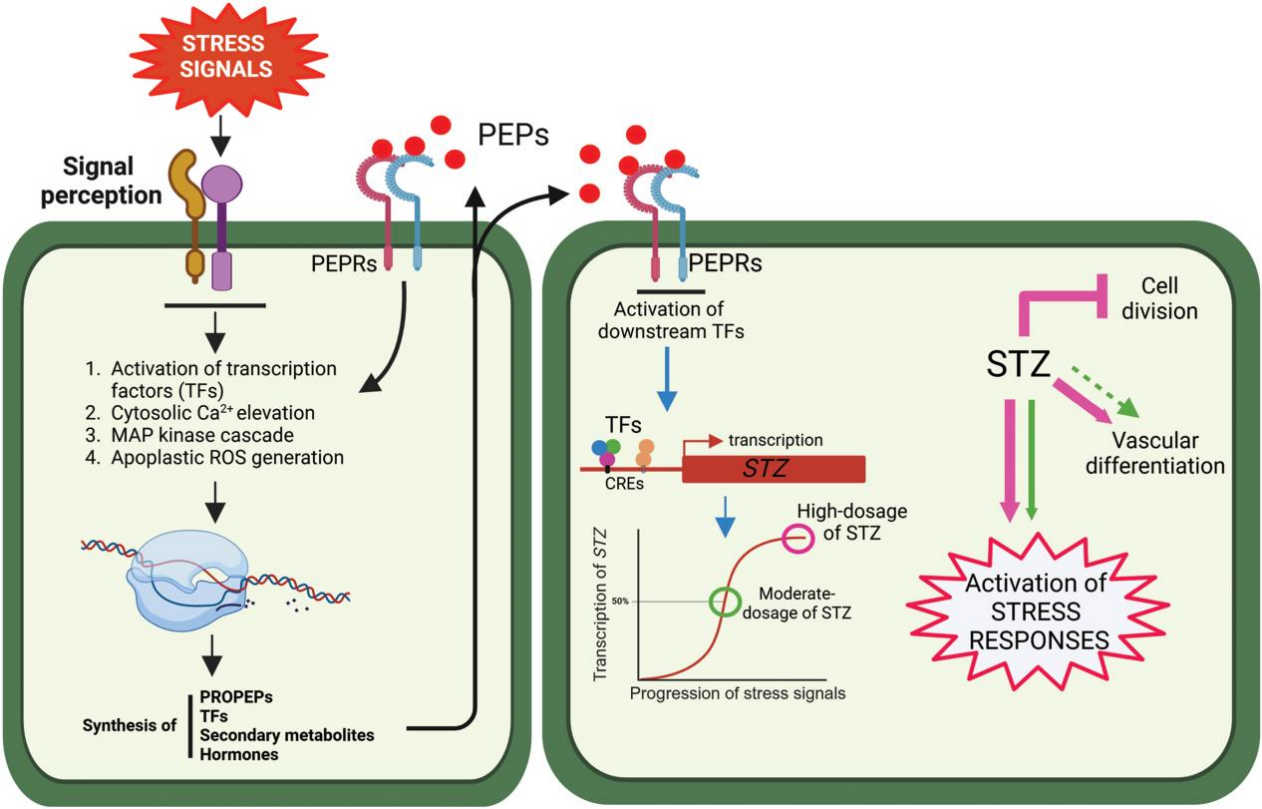
A working model showing how the perception of stress signals leads to a stress-growth tradeoff. In this study, synthetic PEP1 was used as a representative stress-induced DAMP. PEPs, secreted in response to exogenous stress signals, activate a group of core stress response TFs, including STZ. The magnitude of the stress signals controls the transcript levels of STZ, which differentially regulates stress responses and growth in the root meristem. A very high dose of STZ strongly activates stress resistance while compromising root meristem activity and growth (shown in pink arrows; pointed for activation and blunted for repression). In contrast, a moderate dose of STZ activates a stress resistance network without compromising root growth (shown in green arrows), which may be beneficial for sustaining plant yield in a moderately harsh environment. Images were created using BioRender.com. Black arrow, a reported stress-signaling pathway; blue arrow, PEP1-mediated activation of stress-related TF genes, including *STZ*.

This study revealed a comprehensive transcriptional landscape of the root meristem in response to synthetic PEP1, a danger signal activated under biotic and abiotic stress conditions. *STZ* and its homologs appear to serve as a nexus between danger signal perception and molecular reprogramming of the meristem. Our findings suggest that the PEP1-mediated suppression of cell division, induction of cell differentiation, and activation of stress regulators in the root meristem depended on the dose of *STZ*, which varies depending on the exposure or magnitude of the stress signals (Fig. 8). Collectively, these findings suggest the possibility of designing plants with enhanced stress resistance without compromising growth by modulating STZ levels.

## Materials and methods

### Plant materials and growth conditions

The Arabidopsis (*A. thaliana*) plants used in the present study belonged to the Columbia (Col-0) ecotype. Col-0 plants were used as the WT controls. Mutants *stz* (SALK_054092) (Hoang et al. 2020) and *pepr1 pepr2* (Nakaminami et al. 2018), and transgenic lines *ProWOX5:erGFP* (Sebastian et al. 2015) and *ProTMO5::erGFP* (Lee et al. 2006), and PlaCCI (Desvoyes et al. 2020) have been previously reported. All seeds were surface-sterilized, vernalized, and grown on ½ MS medium, which comprises 0.22% Murashige and Skoog medium, 0.025% MES, and 1% sucrose adjusted to pH 5.7 further supplemented with 1% Plant Agar (Duchefa Biochemie) under extended day conditions (16-h/8-h light/dark cycle) with 100 *μ*mol m^−2^ s^−1^ light intensity at 22 to 23 ℃ in a plant growth chamber.

### Root growth and meristem phenotyping

The synthetic PEP1 (ATKVKAKQRGKEKVSSGRPGQHN) was obtained from Peptron (Korea; http://www.peptron.com) (Dhar et al. 2021). The peptide was dissolved in distilled water to make stock solutions of 10 mm each and stored at −20 ℃ until use.

To analyze the root growth in *STZ-OE* seedlings, we surface-sterilized seeds and plated the respective lines of *STZ-OE* into ½ MS medium supplemented with 10 *µ*m estradiol (Sigma-Aldrich, USA). The seeds cold-treated on ½ MS medium were germinated and grown at 22 ℃ in the plant growth chamber under extended day conditions up to 5 d after transfer (DAT) to the growth chamber. For the root growth phenotyping in *STZ-OE*, seedlings grown on ½ MS medium were transferred at 3 DAT to fresh ½ MS medium supplemented with various concentrations of PEP1 and estradiol (1 or 10 *µ*m) and allowed to grow for another 4 d. The transgenic generations (T2/T3/T4/T5) of the *STZ-OE* seedlings used in each experiment are described in the Results section. For root growth phenotyping of *stzl* mutant seedlings germinated and grown on ½ MS medium for 3 DAT were transferred to fresh ½ MS medium supplemented with various concentrations of PEP1 and grown for an additional 4 d. Seedlings in the plates containing ½ MS medium were photographed using a Nikon digital camera, and their root lengths were measured using ImageJ (https://imagej.net/ij/).

Seedlings were harvested and fixed overnight in 4% paraformaldehyde (PFA) to visualize root meristems. Seedlings were washed with 1× phosphate-buffered saline (PBS; 137 mm NaCl, 2.7 mm KCl, 10 mm Na_2_HPO_4_, 1.8 mm KH_2_PO_4_, and pH 7.4) and treated with ClearSee (Kurihara et al. 2015) for 3 to 5 d. Seedlings were stained with 0.1% (v/v) calcofluor white 2MR (Sigma-Aldrich) for at least 1 h and observed using a confocal microscope (Leica TCS SP8 or Zeiss LSM700) at the preset excitation/emission wavelengths of calcofluor white 2MR (laser: 405 nm, laser power: 4.5, gain: 35, emission wavelength: 410 to 458 nm).

### Tissue harvest, RNA extraction, and sequencing

For PEP1 time-course transcriptome data, 4 to 5 DAT Col-0 seedlings were treated with or without (mock treatment) 1 *µ*m PEP1 for 3, 6, 12, and 24 h in such a manner that the age of the seedlings at harvest was 5 DAT. The root meristems were dissected from each set of seedlings, and the tissues were fixed overnight using RNA*later* solution (Sigma-Aldrich). Total RNA was extracted using the RNeasy Plant Micro Kit (Qiagen, Germany) with an on-column DNase digestion step to remove genomic DNA contamination.

RNA samples were prepared to obtain time-course *STZ-OE* RNA-seq data; 4 to 5 DAT STZ-OE #2 (T3) seedlings were treated with an equal volume of ethanol (mock treatment) or 20 *µ*m estradiol for 24 and 6 h, respectively. The bottom half of the roots were dissected from the seedlings at 5 DAT, and total RNA was isolated using RNeasy Plant Mini Kit (Qiagen) with an on-column DNase digestion step following the manufacturer's instructions.

The RNA quality, integrity, and quantity were measured using a NanoVue spectrophotometer (GE Healthcare, USA) and a 2100 Agilent Bioanalyzer with a plant RNA NanoChip assay (Agilent Technologies, USA) (Supplementary Table S26). The library was prepared using the TruSeq Stranded mRNA Library Prep Kit (Illumina, USA) with an average insert size of >300 bp. The NGS library for each sample was sequenced using an Illumina NovaSeq6000 system to obtain 100-bp paired-end reads at Macrogen Inc. (Seoul, Korea). Sequencing files were deposited in the Gene Expression Omnibus (http://www.ncbi.nlm.nih.gov/geo/) with the GEO accession numbers GSE252414 (PEP1 RNA-seq) and GSE252415 (*STZ-OE* RNA-seq).

### Data analysis for RNA-seq

The read quality of the FASTQ files was assessed using FastQC (https://www.bioinformatics.babraham.ac.uk/projects/fastqc/). Trimming of adapter sequences and low-quality reads was performed using the CLC Genomics Workbench (version 22.0.1; Qiagen) with a parameter-quality limit of 0.05. To estimate transcript abundance, high-quality trimmed reads were mapped to the Arabidopsis genome (TAIR10) using the following parameters: mismatch cost, 2; insertion cost, 3; deletion cost, 3; length fraction, 0.8; similarity fraction, 0.8; and the expression levels were normalized as reads per kilobase per million mapped reads (RPKM) using the CLC Genomics Workbench. The DEGs (FDR < 0.01 and |fold change| ≥ 1.5) were determined by comparing the PEP1 or estradiol-treated samples to their respective mock sample.

### PCA, clustering, GO, and KEGG pathway analysis of the DEGs

For PCA, the top 5,000 DEGs were used. The R packages “ggfortify” and “ggrepel” were used to visualize the PCA data. The DEGs were combined and K-means clustered using MeV software (version 4.9.0; Saeed et al. 2003). GO term enrichment analyses were performed using a hypergeometric distribution test and compared against Arabidopsis reference genes. GO and GOSlim categories were assessed using the “BINGO” package (Maere et al. 2005) in Cytoscape (www.cytoscape.org), with significance set at *P* ≤ 0.05. The data presentation was created using the “ggplot2” package in R (Villanueva and Chen 2019).

### Gene expression quantification by RT-qPCR

The bottom half of the roots from seedlings at 5 DAT were dissected, and the total RNA was isolated using RNeasy Plant Mini Kit (Qiagen) with an on-column DNase digestion step following the manufacturer's instructions. The details of the respective mock, PEP1, and estradiol treatments are mentioned in the figure legends. Approximately 2 *µ*g of purified RNA was used as a template for cDNA biosynthesis using SuperScript III Reverse Transcriptase (Invitrogen, USA) in a 20-*µ*L reaction. The synthesized cDNA was diluted 5-fold by adding 80 *µ*L of autoclaved distilled water, and 1 *µ*L of cDNA was used as a template for RT-qPCR using iQ SYBR Green Supermix (BIO-RAD, USA) on a CFX96 Real-Time PCR machine (BIO-RAD, USA) following the manufacturer's instructions (95 ℃ for 3 min, followed by 40 cycles of 95 ℃ for 20 s, 58 ℃ for 20 s, 72 ℃ for 30 s) (Kim et al. 2020; Dhar et al. 2021). The sequences of the gene-specific primers are given in Supplementary Table S27.

### Determination of the numbers of *STZL* transcripts in the root meristem through ddPCR

The meristem regions (∼750 *µ*m long root tips) were dissected from the roots of WT Col-0 and *STZ-OE* seedlings at 5 DAT. WT Col-0 seedlings were treated with either 1 *µ*m of PEP1, or 1 or 10 *µ*m of estradiol for 24 h before dissection. *STZ-OE* #2 seedlings were treated with 1 or 10 *µ*m of estradiol, and STZ #6 and #9 seedlings were treated with 10 *µ*m of estradiol for 24 h before dissection. The root meristems dissected from each set of seedlings were kept in RNA*later* solution (Sigma-Aldrich) overnight, and then, the total RNA was isolated using a RNeasy Plant Micro Kit (Qiagen). Total RNA (1 *µ*g) for reverse transcription was obtained using SuperScript III Reverse Transcriptase (Invitrogen) in a 20-*µ*L reaction. The synthesized cDNA was diluted to 100 *µ*L by adding 80 *µ*L of autoclaved distilled water. Droplet generation, PCR, quantification, and data analysis were performed as described previously (Kim et al. 2020; Hoang et al. 2022). The PCR steps were as follows: 1 cycle at 95 ℃ for 5 min, 40 cycles of 95 ℃ for 30 s and 60 ℃ for 1 min, followed by 1 cycle of 4 ℃ for 5 min and 90 ℃ for 5 min. The gene-specific primers used for the ddPCR are listed in Supplementary Table S27.

### Molecular cloning and generation of transgenic plants

To generate transcriptional reporter lines of *STZ*, *AZF3,* and *ZAT6*, MultiSite Gateway system (Invitrogen) was used as previously described (Lee et al. 2006; Kim et al. 2020). The promoter regions of *STZ* (Sakamoto et al. 2004), *AZF3* (Sakamoto et al. 2004), and *ZAT6* (Shi et al. 2014) were amplified from WT Col-0 genomic DNA using PCR and inserted into pDONR P4-P1r via BP recombination. The reporter component, *eGFPGUS* (Furuta et al. 2014) was cloned into pDONR221. To obtain *pSTZ::eGFPGUS*, *pAZF3::eGFPGUS*, and *pZAT6::eGFPGUS*, final recombination was performed in the binary vector dpGreenBarT using Multisite Gateway LR recombination. To create the translational fusion lines of *STZ* (*pSTZ::STZ-GFP*), AZF3 (*pAZF3::AZF3-GFP*), and *ZAT6* (*pZAT6::ZAT6-GFP*), the coding sequences of *STZ*, *AZF3*, and *ZAT6* were amplified using cDNA from Col-0 root and cloned into pENTR/D-TOPO vector (Thermo Fisher Scientific, USA). Each cDNA clone was inserted into the binary vector dpGreenBarT with the previously prepared cassettes of the respective promoters at pDONR P4-P1r and GFP at pDONR P2R-P3 using LR Clonase II Plus (Invitrogen).

To generate the inducible overexpression lines of *STZ* (*STZ-OE*), pDONR P4-P1r vector containing *p35S::XVE>>pLexA* was obtained from Dr. Ari Pekka Mähönen (Siligato et al. 2016). The *p35S::XVE>>pLexA::STZ* was constructed into dpGreenBarT by LR recombination. All the clones in the binary vector were introduced into Agrobacterium GV3101 using pSOUP for Arabidopsis transformation via floral dipping (Clough and Bent 1998). Primer sequences used for cloning are listed in Supplementary Table S27.

### Generation of CRISPR-based mutant lines

We used CRISPR–Cas9 technology to generate single- and double-mutant combinations (Ursache et al. 2021). The guide RNA (gRNA) was designed using the web-based program CRISPR-PLANT (www.omap.org/crispr) (Xie et al. 2014). Because the coding sequences were small (ZAT6 = 717 bp and AZF3 = 582 bp) for each gene, we designed 2 gRNAs (Supplementary Table S27). The first and second gRNAs were cloned into pRU41 and pRU42 vectors, respectively, following a previously described method (Ursache et al. 2021). Golden gate assembly of gRNAs was performed to generate a single cassette containing both gRNAs in the pSF463 vector (Ursache et al. 2021). Finally, the gRNA cassettes containing the 2 gRNAs were switched to the gateway-compatible pRU53 (*AZF3* gRNA) and pRU54 (*ZAT6* gRNA) vectors using LR recombination. A schematic representation of the cloning is shown in Supplementary Fig. S22.

To generate the *stz azf3* and *stz zat6* double mutants, *AZF3* (pRU53 with FastRed) and *ZAT6* (pRU53 with FastGreen) gRNA-Cas9 transgenes were introduced into *stz* mutant by floral dipping. The T1 generation was screened using an INCell 2000 analyzer (GE Healthcare) based on fluorescence. To generate the *azf3 zat6* double mutant, gRNAs for *AZF3* (pRU53 with FastRed) and *ZAT6* (pRU54 with FastGreen) were introduced to WT Col-0 seedlings. The primary selection for the T1 generation was performed based on seeds with both FastRed and FastGreen signals. The seedlings used in the experiment were the T3 generation and contained Cas9 in their genome. To confirm the nucleotide changes, the target regions were amplified from the genomic DNA of the transgenic plants. The sequencing results are shown in Supplementary Fig. S22, and the primers used for this experiment are listed in Supplementary Table S27.

### Grafting between Col-0 and *STZ-OE* seedlings

We performed reciprocal grafting using WT Col-0 and *STZ-OE* #2 seedlings as previously reported (Lee et al. 2023). The hypocotyls of seedlings at 4 DAT were cut using a sterile surgical blade under a dissection microscope (Leica S6E, Leica, Germany), and WT Col-0 or *STZ-OE* scion shoots were carefully placed on top of *STZ-OE* or WT Col-0 rootstocks. The grafted seedlings were recovered on sterile water-soaked filter paper at 28 °C under an extended day condition for 6 d. Once the vascular connections were formed, the seedlings were transferred to ½ MS medium and grown for 3 d for further recovery. Adventitious roots from the scions were removed regularly. Seedlings 9 d post-grafting on solid ½ MS medium were briefly flooded with 10 *µ*m of estradiol diluted in liquid ½ MS solution. Seedlings were processed for imaging of their meristems 48 h after estradiol treatment using ClearSee.

### Root growth sensitivity experiment after transient overexpression (priming) of *STZ*

To identify whether transient overexpression of *STZ* affects PEP1-responsive root growth behavior, we performed the following priming experiment. Briefly, the WT Col-0 and *STZ-OE* #2, #6, and #9 seedlings were germinated and grown on ½ MS medium at 3 DAT and flooded with an equal volume of 10 *µ*m estradiol diluted in liquid ½ MS solution under a sterile laminar air flow hood. After 30 min of treatment, the excess estradiol solution was decanted, and the seedlings were further grown vertically. In 24 h post-priming with *STZ* overexpression, the seedlings were transferred to ½ MS medium supplemented with or without PEP1 at different concentrations. Seedlings on the MS plates were photographed at the time of transfer (4 DAT) and collection (7 DAT) using a Nikon digital camera, and root lengths were measured using ImageJ software (https://imagej.net/ij/). These data are presented in Supplementary Table S25.

### Imaging of transcriptional and translational marker lines

To identify the expression patterns of transcriptional and translational reporter lines, seedlings 4 DAT were treated with 1 *µ*m PEP1 for 24 h before they were imaged using a confocal microscope (Leica TCS SP8 and Zeiss LSM700). To visualize the GFP protein, seedlings were stained with 1 *µ*m propidium iodide (PI; Life Technologies) solution for 1 min and observed with the following excitation and detection windows: GFP, 488 nm/500 to 530 nm; PI, 555 nm/591 to 635 nm. For the Z-scan imaging, the same positions of the root and laser scanning area were selected. GUS staining was performed following standard methods (Jefferson et al. 1987), and the seedlings were photographed using a digital microscope (Nikon, Japan) with DIC optics.

### Histological analysis

To analyze the stele internal tissue organization, seedlings at 5 DAT grown on ½ MS medium supplemented with 10 *µ*m estradiol were used. Five to 6 seedlings were arranged within a 4% low-melting SeaPlaque, Agarose (Lonza) blocks. The solidified agarose was dissected into 120 to 130 *µ*m root sections using a vibratome (Leica VT1000S). Sections of WT (MS), WT (estradiol), and *STZ-OE* #2 (T3) (MS) samples were created using the 4 to 6 mm basal region from the root tip and observed using a confocal microscope (Zeiss LSM700), as previously reported (Kim et al. 2020; Seo and Lee 2021). Due to the small roots of *STZ-OE* #2 (T3) (estradiol) seedlings, plastic sectioning was performed to determine the internal tissue organization. Seedlings were harvested and fixed overnight in 4% PFA at room temperature. The samples were then washed with 1× PBS and dehydrated in an ethanol series [30%, 50%, 70%, 90%, and 100% (v/v)], and the plastic blocks were prepared using the Technovit 8100 kit according to the manufacturer's instructions. As described previously, 5-µm-thick plastic sections were created from the 1-mm basal region of root tips using a Leica RM2255 microtome (Kim et al. 2020; Dhar et al. 2021). Sections were photographed using a Nikon Eclipse N*i*-U microscope with DIC optics (Supplementary Fig. S17).

### Lugol's staining

The roots of the seedlings were submerged in Lugol's solution (Sigma-Aldrich) for 1 to 2 min. After staining, the roots were mounted on microscope slides using chloral hydrate. The root tips were photographed using DIC optics on an Axio Imager A1 microscope (oil immersion 40× objective; Zeiss, Germany).

### Phylogenetic tree analysis

Alignment and phylogenetic reconstructions were performed using the function “build” of ETE3 3.1.3 (Huerta-Cepas et al. 2016) as implemented on the GenomeNet (https://www.genome.jp/tools/ete/). A distance-based tree was inferred with the BioNJ algorithm (Gascuel 1997) using PhyML v20160115 (Guindon et al. 2010) ran with model and parameters: --pinv e --alpha e --nclasses 4 -o lr -f m --bootstrap --2.

### Quantification and statistical analysis

Statistical analyses for RT-qPCR and root growth phenotyping were performed using Microsoft Excel and are provided in Supplementary Tables S23, S25, and S29.

Plots and graphs were generated using the “ggplot2,” “tidyverse,” and “pheatmap” packages in R software (https://www.rstudio.com) and Excel. Venn diagrams were constructed using an online tool (http://bioinformatics.psb.ugent.be/webtools/Venn). All analyses in CLC Genomics Workbench (Qiagen) and the Linux environment were conducted on a local server hosted by the Plant Systems Genetics Laboratory at Seoul National University, Korea.

### Accession numbers

The RNA-seq data reported in this study were submitted to the NCBI GEO database (http://www.ncbi.nlm.nih.gov/geo) with accession numbers GSE252414 (PEP1 RNA-seq.) and GSE252415 (*STZ-OE* RNA-seq). The information of the genes used in this study is listed in the Arabidopsis Genome Initiative under the following accession numbers: AT4G33550, *ABI5* (AT2G36270), *ANAC2* (AT1G01720), *ANAC055* (AT3G15500), *ANAC062* (AT3G49530), *ANAC087* (AT5G18270), *AZF3* (AT5G43170), *BFN1* (AT1G11190), *CDKB2;1* (AT1G76540), *CEP1* (AT5G50260), *CYCB1;4* (AT2G26760), *CYCB2;3* (AT1G20610), *CYCB2;4* (AT1G76310), *CZF1* (AT2G40140), *DAG1* (AT3G61850), *DALL3* (AT2G30550), *ERF2* (AT5G47220), *ERF104* (AT5G61600), *GAPDH* (AT1G13440), *GRXS13* (AT1G03850), *HCA2* (AT5G62940), *HSF4* (AT4G36990), *LECRKA4.1* (AT5G01540), *MAP65-4* (AT3G60840), *MC9* (AT5G04200), MP (AT1G19850), *MYB15* (AT3G23250), *OBP4* (AT5G60850), *PEPR1* (AT1G73080), *PEPR2* (AT1G17750), *PIF1* (AT2G20180), *RNS3* (AT1G26820), *RAB18* (AT5G66400), *SCPL48* (AT3G45010), *STZ* (AT1G27730), *TMO5* (AT3G25710), *TMO6* (AT5G60200), *UBQ10* (AT4G05320), *VND6* (AT5G62380), *WOX5* (AT3G11260), *WRKY25* (AT2G30250), *WRKY46* (AT2G46400), and *ZAT6* (AT5G04340).

## Supplementary Material

koae327_Supplementary_Data

## Data Availability

All data and materials that support the findings of this study are included in the manuscript or in the Supplementary Materials.

## Supplementary data

The following materials are available in the online version of this article.

**Supplementary Figure S1.** The root apical growth in response to 1 *μ*m PEP1.

**Supplementary Figure S2.** The DEGs in PEP1-treated time-course RNA-seq experiment.

**Supplementary Figure S3.** Cell-type-specific expression patterns of PEP1-repressed Clusters 9 and 11.

**Supplementary Figure S4.** Expression of PEP1-induced TFs is enriched within the root stele.

**Supplementary Figure S5.** *STZ* is 1 of the hub genes of 344 PEP1-regulated TFs.

**Supplementary Figure S6.** Expression profile of the transcriptional and translational fusion lines of STZL without and with PEP1 treatment.

**Supplementary Figure S7.** Expression dynamics of the transcriptional fusion lines of STZL with time-course PEP1 treatment.

**Supplementary Figure S8.** *STZ* is downstream of the PEP1-PEPR perception pathway.

**Supplementary Figure S9.** Validation of *STZ-OE* transgenic lines.

**Supplementary Figure S10.** Meristem images of *STZ-OE* transgenic lines expressing different dosages of *STZ*.

**Supplementary Figure S11.** *STZ* overexpression locally suppresses cell division at the root meristem.

**Supplementary Figure S12.** The root apical growth of *STZ-OE* seedlings in response to estradiol treatment for 24 h.

**Supplementary Figure S13.** Root growth rescue test of the *STZ-OE* seedlings.

**Supplementary Figure S14.** Root growth rescue test using PEP1-treated Col-0 seedlings.

**Supplementary Figure S15.** Overexpression of *STZ* deforms QC identity.

**Supplementary Figure S16.** Additional images of the Lugol's stained root tips of *STZ-OE* and PEP1-treated Col-0 seedlings.

**Supplementary Figure S17.** The cross-sectional images of roots used to quantify xylem vessel distribution phenotypes.

**Supplementary Figure S18.** STZ transcriptionally regulates cell cycle, dPCD, ePCD, and stress-related genes, as evidenced by the time-course RNA-seq experiment using *STZ-OE* seedling roots.

**Supplementary Figure S19.** Replicate analysis of the contribution of STZ dosages to the PEP1-induced root growth suppression.

**Supplementary Figure S20.** Confocal microscopy showing additional root meristem phenotypes from *STZ-OE* and *stzl* seedlings grown in the presence of PEP1.

**Supplementary Figure S21.** Transient overexpression of *STZ* led to sustained root growth of *STZ-OE* seedlings under PEP1 treatment.

**Supplementary Figure S22.** Schematic map for gRNA cloning.

**Supplementary Figure S23.** Confirmation of CRISPR–Cas9-based mutation to generate *stzl* mutant combinations.

**Supplementary Table S1.** Summary table of PEP1-treated samples and RNA-seq reads.

**Supplementary Table S2.** The RPKM (reads per kilobase of transcript per million mapped reads) with the corresponding gene IDs in the transcriptome dataset.

**Supplementary Table S3.** A list of 8,296 DEGs identified from PEP1-treated samples compared with the mock samples (FC ≥ 1.5; FDR < 0.01) and their expression values in RPKM (reads per kilobase of transcript per million mapped reads).

**Supplementary Table S4.** K-means clustering of the 8,296 DEGs regulated by PEP1.

**Supplementary Table S5.** GO enrichment (biological processes) analysis of DEGs induced (Clusters 1, 3, 5, and 7) and repressed (Clusters 2, 6, 8, 9, and 11) by PEP1 by BINGO (corrected *P*-value ≤0.01).

**Supplementary Table S6.** GOSlim analysis of DEGs induced (Clusters 1, 3, 5, and 7) by PEP1 using BINGO (corrected *P*-value ≤0.05).

**Supplementary Table S7.** List of TFs from PEP1-induced Clusters 1, 3, 5, and 7.

**Supplementary Table S8.** Classification of the 344 PEP1-induced TFs based on protein domains.

**Supplementary Table S9.** The in silico interaction scores between PEP1-induced TFs and the degree of each TF.

**Supplementary Table S10.** The in silico interaction scores of a network between PEP1-induced TFs with genes in the Cluster 9.

**Supplementary Table S11.** DAP-seq predicted STZ targets among the PEP1-suppressed cell cycle genes.

**Supplementary Table S12.** Frequency of xylem vessel distribution in the roots of *STZ-OE* #2 (T3) seedlings.

**Supplementary Table S13.** Summary table of *STZ-OE* #2 (T3) samples used for RNA-seq and RNA-seq reads.

**Supplementary Table S14.** The RPKM (reads per kilobase of transcript per million mapped reads) with the corresponding gene IDs in the STZ-OE RNA-seq dataset.

**Supplementary Table S15.** List of 8,435 DEGs identified from *STZ-OE* RNA-seq datasets compared with the mock samples (FC ≥ 1.5; FDR < 0.01) and their expression values in RPKM (reads per kilobase of transcript per million mapped reads).

**Supplementary Table S16.** K-means clustering of the 8,435 genes differentially regulated by STZ.

**Supplementary Table S17.** List of 61 common cell cycle genes that both STZ and PEP1 suppress.

**Supplementary Table S18.** GO term (biological processes) enrichment analysis of the 61 cell-cycle-related DEGs suppressed by both PEP1 and *STZ-OE* (corrected *P*-value ≤0.05).

**Supplementary Table S19.** Arabidopsis root cell-type-specific expression (Brady et al. 2007; Zhang et al. 2019) patterns of the STZ-regulated cell cycle, dPCD, ePCD, and stress-related genes.

**Supplementary Table S20.** Common dPCD and ePCD genes that STZ upregulates.

**Supplementary Table S21.** List of 190 among 344 TFs induced by PEP1, which are differentially expressed under *STZ-OE*.

**Supplementary Table S22.** Stress regulators connected with STZ on the STRING database and differentially expressed by both PEP1 and *STZ-OE*.

**Supplementary Table S23.** Expression levels of the selected cell cycle, dPCD, ePCD, and stress-responsive genes in *STZ-OE* #2 (T3) and *STZ-OE* #9 (T3) lines as measured by RT-qPCR in Arabidopsis roots.

**Supplementary Table S24.** Stress regulators downstream of STZ, selected for RT-qPCR, presented in Fig. 6D.

**Supplementary Table S25.** Root growth data of the *STZ-OE* lines and *stzl* mutants under PEP1.

**Supplementary Table S26.** Concentration, RIN values, and rRNA ratios of RNA samples used for RNA-seq in this study.

**Supplementary Table S27.** A list of primers used in this study.

**Supplementary Table S28.** Description of the abbreviated cell-type-specific markers/tissue layers.

**Supplementary Table S29.** Results of the statistical tests performed.

## Dive Curated Terms

The following phenotypic, genotypic, and functional terms are of significance to the work described in this paper:

STZ Gramene: AT1G27730
STZ Araport: AT1G27730

CDKB2;1 Gramene: AT1G76540
CDKB2;1 Araport: AT1G76540

CYCB1;4 Gramene: AT2G26760
CYCB1;4 Araport: AT2G26760

CYCB2;3 Gramene: AT1G20610
CYCB2;3 Araport: AT1G20610

CYCB2;4 Gramene: AT1G76310
CYCB2;4 Araport: AT1G76310

WRKY33 Gramene: AT2G38470
WRKY33 Araport: AT2G38470

WRKY40 Gramene: AT1G80840
WRKY40 Araport: AT1G80840

ZAT6 Gramene: AT5G04340
ZAT6 Araport: AT5G04340

AZF3 Gramene: AT5G43170
AZF3 Araport: AT5G43170

PEPR1 Gramene: AT1G73080
PEPR1 Araport: AT1G73080

PEPR2 Gramene: AT1G17750
PEPR2 Araport: AT1G17750

WOX5 Gramene: AT3G11260
WOX5 Araport: AT3G11260

ZAT12 Gramene: AT5G59820
ZAT12 Araport: AT5G59820

DREB2A Gramene: AT5G05410
DREB2A Araport: AT5G05410
