# Supplementary material for: The molecular framework balancing growth and defense in response to plant elicitor peptide-induced signals in Arabidopsis
Source: Plant Cell. 2024 Dec 19;37(1):koae327. doi: 10.1093/plcell/koae327 (PMC11684079; doi:10.1093/plcell/koae327)
Supplement: koae327_Supplementary_Data [file koae327_supplementary_data.zip › Supplemental figures_TPC.pdf]

Supplementary Figures

The molecular framework balancing growth and defense in response to PEP-induced signals in Arabidopsis roots

Souvik Dhar, Soo Youn Kim, Heeji Shin, Jongsung Park, and Ji-Young Lee*

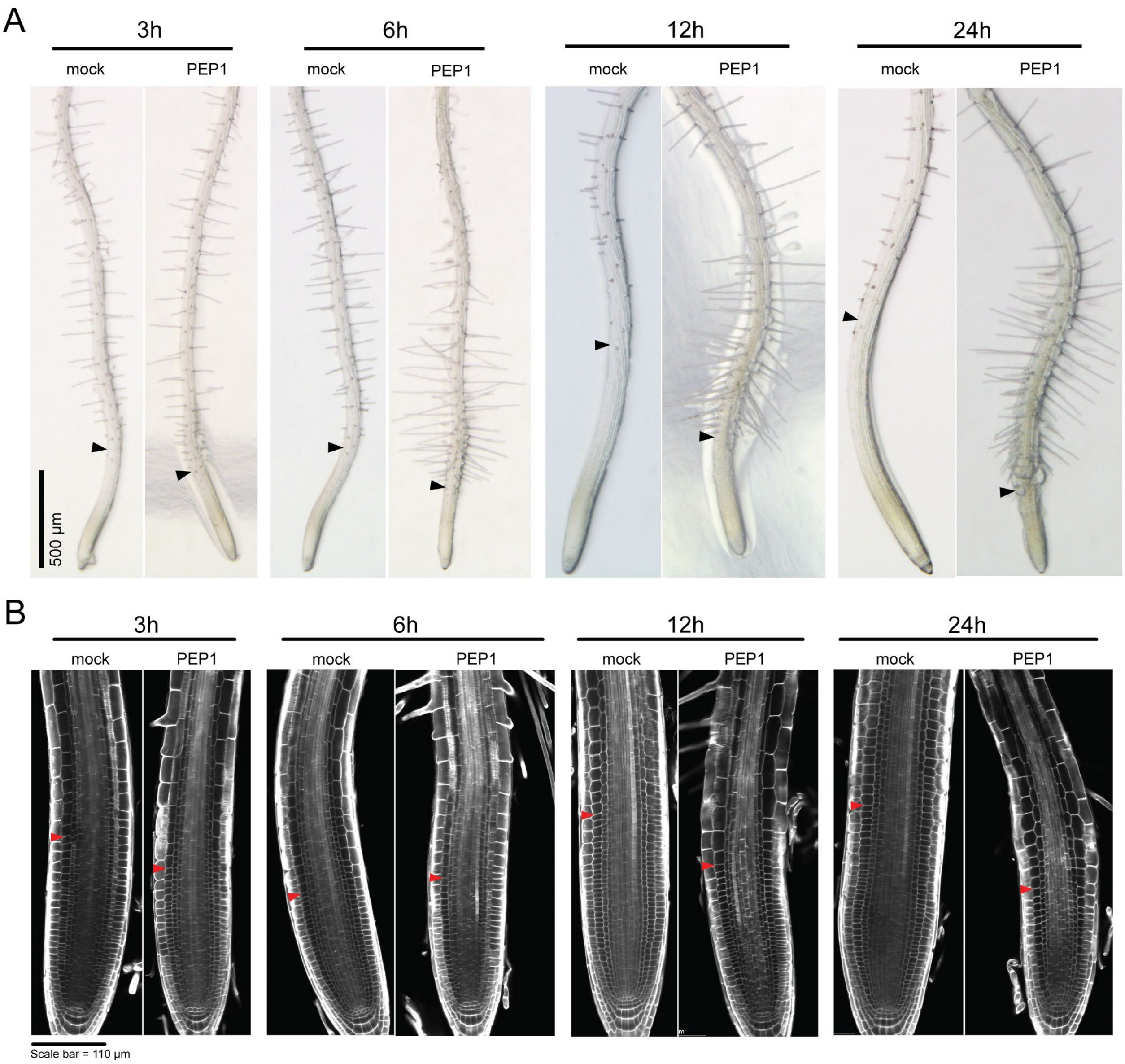

Supplementary Figure S1. The root apical growth in response to 1 µM PEP1 (Supports Figure 1).

(A) The roots of seedlings at 4 DAT were treated with or without (mock treatment) 1 µM PEP1 for 3, 6, 12, and 24 h. Black arrowheads indicate the region from which the root was dissected for RNA sample preparation. Scale bar = 500 µm. (**B)** Confocal microscopy of the root meristems of Col-0 seedlings under the same conditions as described in panel A. Red arrowheads indicate the junctions between the meristem and elongation zones. Scale bar = 110 µm. The scale bars in A and B are applicable to all images under the same panel. DAT = Days After Transfer to the growth chamber.

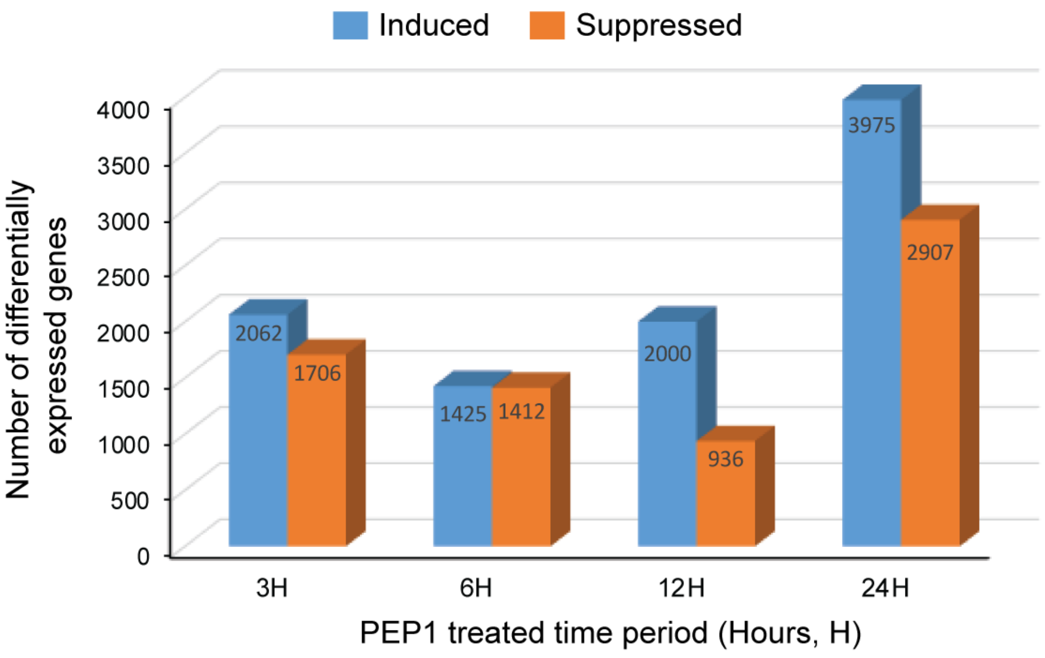

Supplementary Figure S2. The differentially expressed genes (DEGs) in PEP1-treated time-course RNA-seq experiment (Supports Figure 1).

The digits on the bar graph indicate the number of DEGs (Fold-change≥1.5 fold; false discovery rate (FDR) < 0.01) that were induced and suppressed in our time-course PEP1 treatment data.

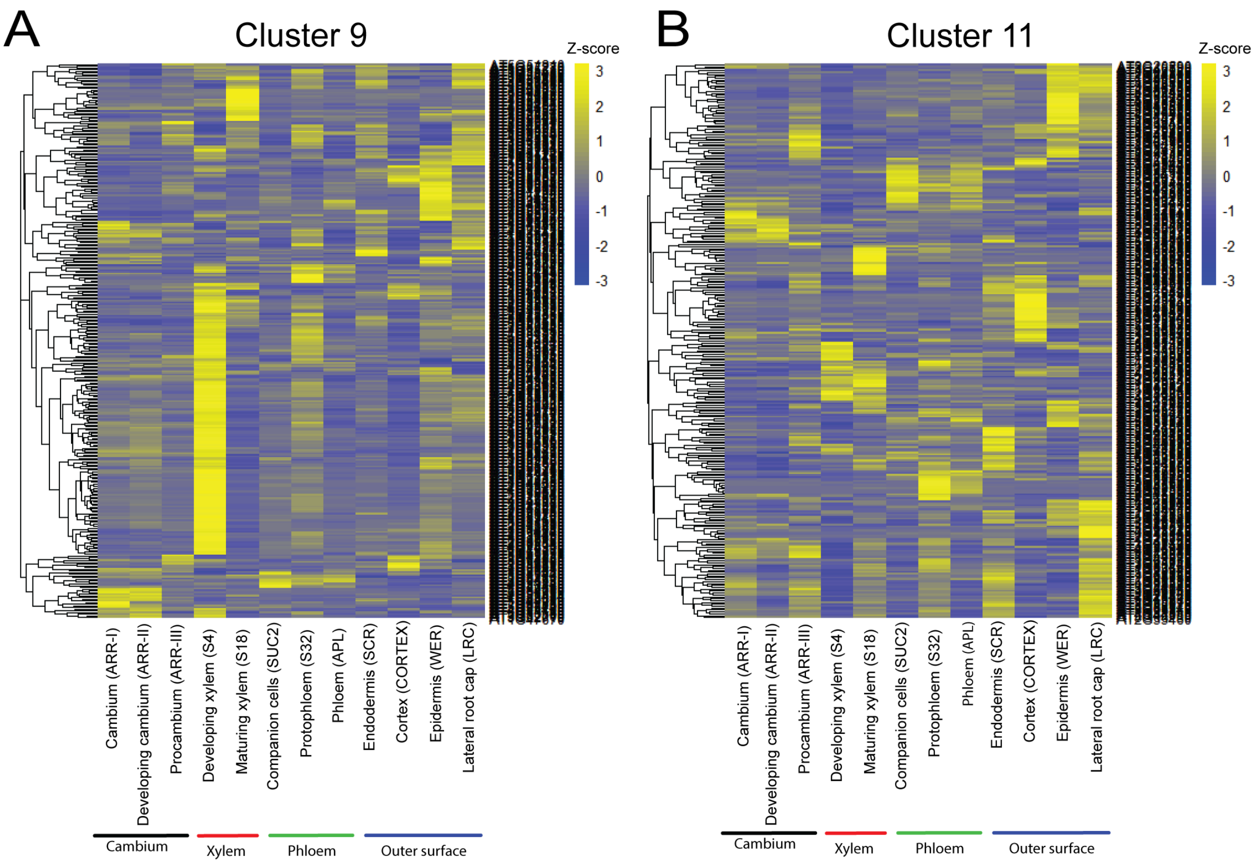

Supplementary Figure S3. Cell-type specific expression patterns of PEP1-repressed clusters 9 and 11 (Supports Figure 1).

(A) Cluster 9, which contained PEP1-repressed cell cycle genes, was highly enriched in the developing xylem (S4). The data were normalized by gene (row-wise) and plotted using the "pheatmap" package in R. (**B)** Cell type-specific expression patterns of PEP1-suppressed in cluster 11, which contained genes involved mainly in root development and morphogenesis. The expression of these genes is enriched in various root cell types. The color scale bars denote the Z-score obtained through the "scale" function while plotting using the "pheatmap" package in R.

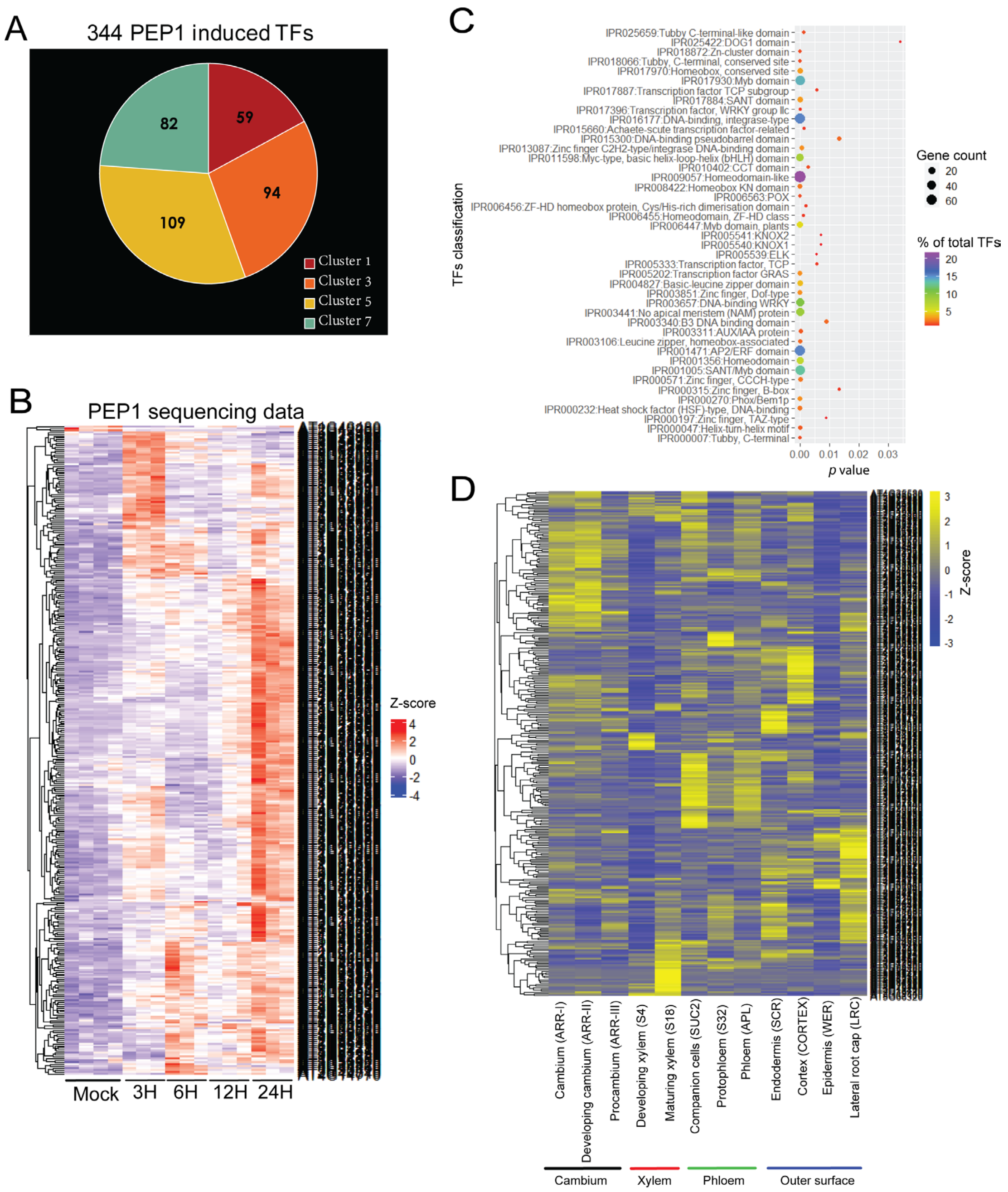

Supplementary Figure S4. Expression of PEP1-induced transcription factors (TFs) is enriched within the root stele (Supports Figure 1).

(A) Pie chart showing the number of TFs induced by PEP1 in clusters 1, 3, 5, and 7. (**B)** Heatmap showing the expression patterns of 344 TFs in the four PEP1-activated clusters. The expression values were normalized along the rows. The color scale bar denotes the z-score. (**C)** The 344 TFs were classified into 43 distinct categories based on their protein domains using the InterPro database (https://www.ebi.ac.uk/interpro); data are presented in Supplementary Table S8. The

dot plot represents the classification from Supplementary Table S8. (**D)** Cell type-specific data exhibited enrichment of PEP1-induced TFs within the stele (cambium, xylem, and phloem). The color scale bar denotes the Z-score obtained through the “scale” function while plotting using the “pheatmap” package in R.

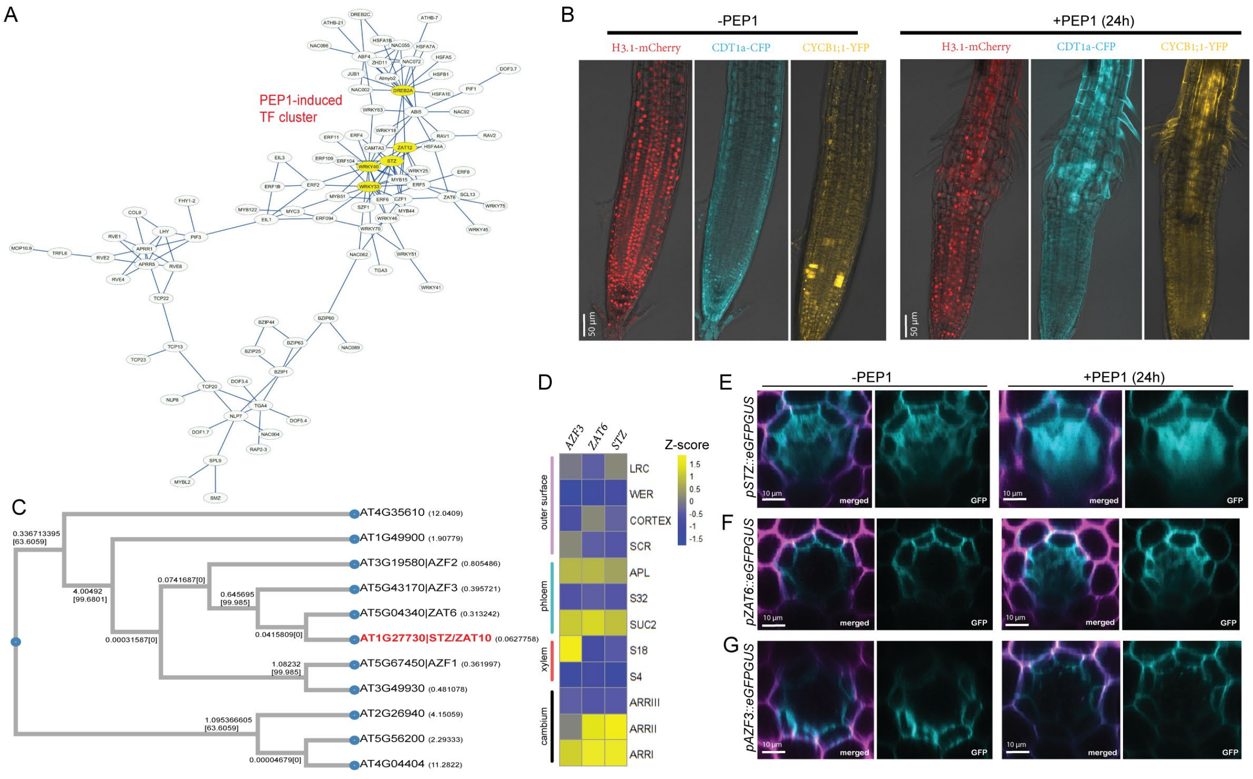

Supplementary Figure S5. STZ is one of the hub genes of 344 PEP1-regulated transcription factors (TFs) (Supports Figure 2).

(**A**) An interaction network of 344 PEP1-induced TFs as inferred from the STRING database (version 12.0; interaction score > 0.7). The interaction scores are listed in Supplementary Table S9. The nodes highlighted in "yellow" indicate the top five highly connected TFs among the 344 PEP1-induced TFs clusters obtained from the STRING database. (**B)** Plant Cell Cycle Indicator (PlaCCI) exhibited suppression of cell cycle marker genes in the meristems of seedlings at 5 DAT after 24 h of 1 µM PEP1 treatment. (**C)** The phylogenetic tree generated on GenomeNet (https://www.genome.jp/tools/ete/) indicates that *ZAT6* (AT5G04340) and *AZF3* (AT5G43170) are the closest homologs of *STZ* (AT1G27730). Branch supports are the Chi2-based parametric values returned by the approximate likelihood ratio test. (**D)** *STZ*, *AZF3*, and *ZAT6* were enriched in the stele. Expression values were normalized by gene expression (column-wise). The color scale bar denotes the Z-score obtained through the "scale" function while plotting using the "pheatmap" package in R. (**E-G)** The cross-sectional (Z-stack) confocal images of the transcriptional reporter lines of *STZ* (*pSTZ::eGFP-GUS*), *ZAT6* (*pZAT6::eGFP-GUS*), and *AZF3* (*pAZF3::eGFP-GUS*) in the root differentiation zone following 24 h of 1 µM PEP1 treatment. *STZ* and *ZAT6* exhibited significant enrichment within the stele 24 h after PEP1 treatment.

Images were obtained from the roots of seedlings at 5 DAT with or without 24 h of PEP1 treatment. DAT = Days After Transfer to the growth chamber.

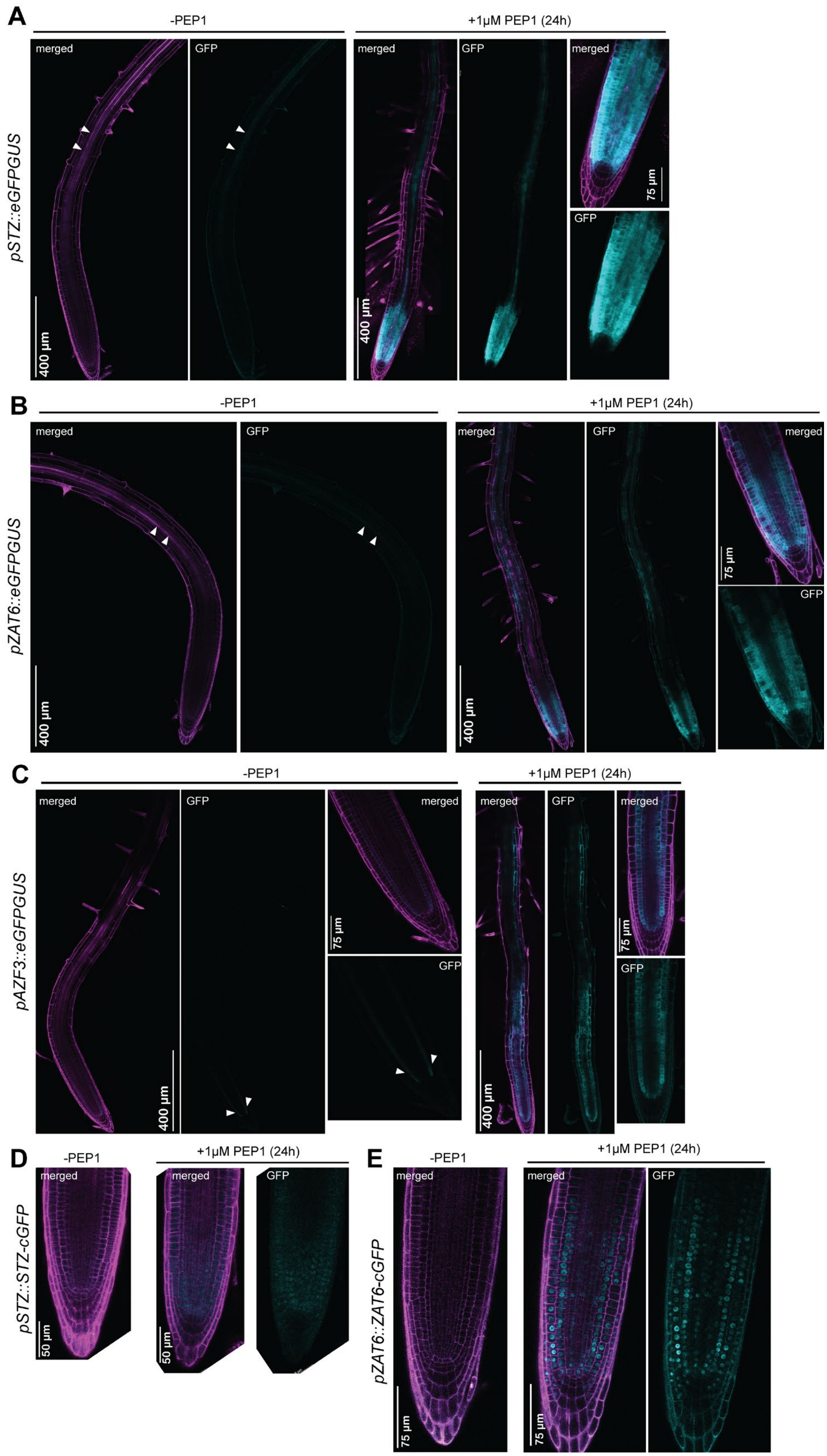
A
-PEP1
+1μM PEP1 (24h)
merged
GFP
pSTZ::eGFPGUS
400 μm
75 μm
B
-PEP1
+1μM PEP1 (24h)
merged
GFP
pZAT6::eGFPGUS
400 μm
75 μm
C
-PEP1
+1μM PEP1 (24h)
merged
GFP
pAZF3::eGFPGUS
400 μm
75 μm
D
-PEP1
+1μM PEP1 (24h)
merged
GFP
pSTZ::STZ-cGFP
50 μm
E
-PEP1
+1μM PEP1 (24h)
merged
GFP
pZAT6::ZAT6-cGFP
75 μm

Supplementary Figure S6. Expression profile of the transcriptional and translational fusion lines of STZL without and with PEP1 treatment (Supports Figure 2).

(A-C) Confocal microscopy of expression patterns of transcriptional fusion lines of *STZ* (*pSTZ::eGFP-GUS*), *ZAT6* (*pZAT6::eGFP-GUS*), and *AZF3* (*pAZF3::eGFP-GUS*) at 5 DAT with or without 1 μM PEP1 treatment for 24 h. White arrowheads indicate a faint GFP signal in the roots without PEP1 treatment. The confocal root images shown are the composite images obtained through tile scanning. (**D** and **E**) The translational fusion lines of *STZ* (*pSTZ::STZ-cGFP*) and *ZAT6* (*pZAT6::ZAT6-cGFP*) exhibit visible protein localization in the meristem following PEP1 treatment. The imaging was performed on seedlings at 4 DAT after treating 1 μM PEP1 at 3 DAT for 24 h. DAT = Days After Transfer to the growth chamber.

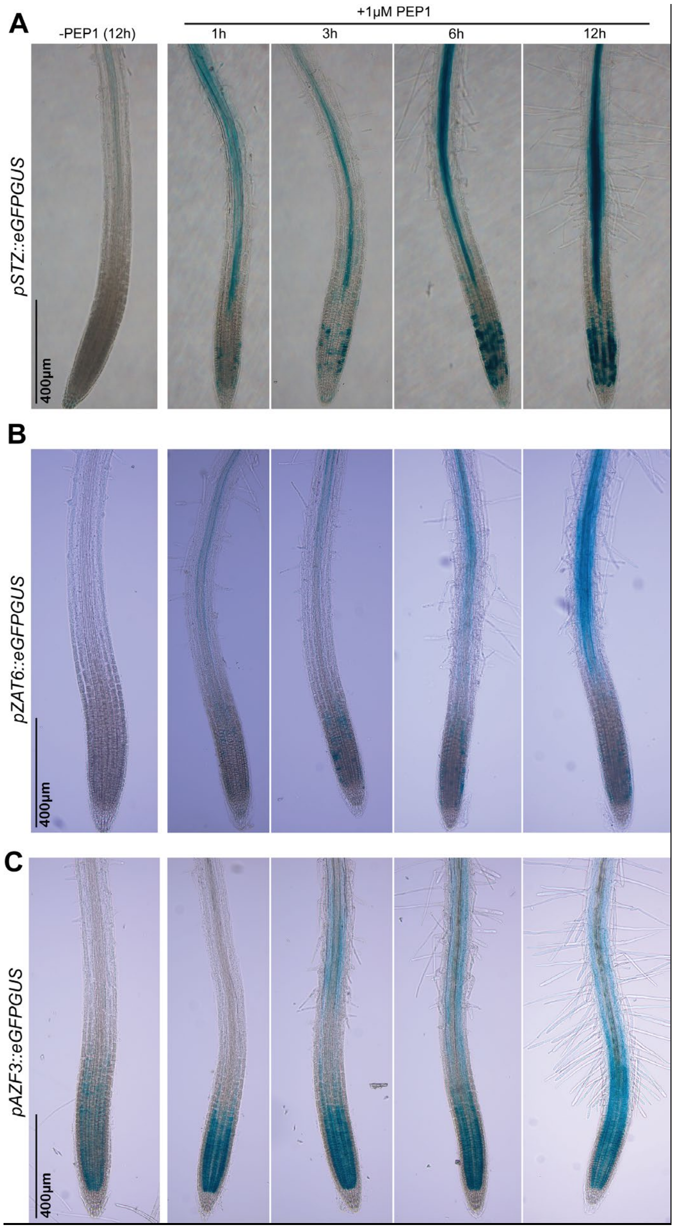

Supplementary Figure S7. Expression dynamics of the transcriptional fusion lines of STZL with time-course PEP1 treatment (Supports Figure 2).

(A-C) Expression patterns of *STZL* (*STZ*, *ZAT6*, and *AZF3*) after treatment with 1 µM PEP1 at different time points as determined by GUS staining. We used WT Col-0 seedlings at 4 DAT harboring transcriptional constructs of *STZ* (*pSTZ::eGFP-GUS*), *ZAT6* (*pZAT6::eGFP-GUS*), and *AZF3* (*pAZF3::eGFP-GUS*) and treated them with 1 µM PEP1 for 1, 3, 6, and 12 h. "without PEP1 (12 h)" was used as an experimental control. The scale bars mentioned in A, B, and C are applicable to all images under the same panel. DAT = Days After Transfer to the growth chamber.

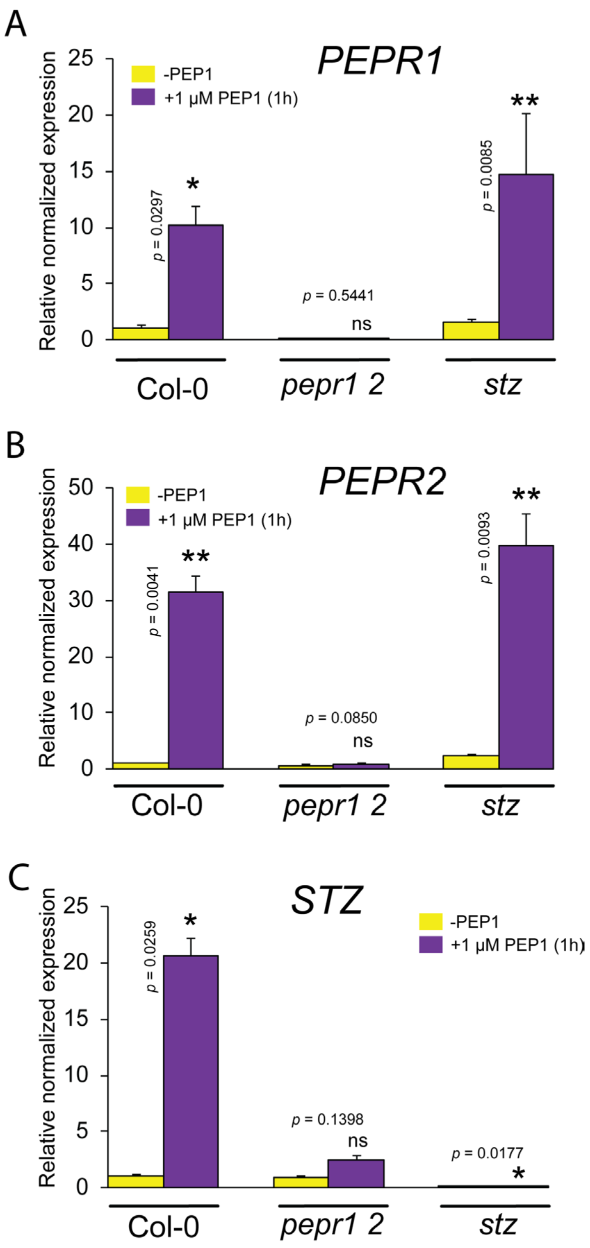

Supplementary Figure S8. *STZ* is downstream of the PEP1-PEPR perception pathway (Supports Figure 3).

(A-C) Expressions of *PEPR1*, *PEPR2*, and *STZ* in Col-0, *pepr1 2*, and *stz* genotypes at 1 h of 1 µM PEP1 treatment, measured by RT-qPCR. The data are presented as ± SEM from three technical replicates. *GAPDH* was used as the internal control. The expression values for "–PEP1" samples for each genotype were arbitrarily set as 1, and the statistical significance of differences was compared with the "+1µM PEP1 (1h)" sample using Student's t-test (**P < 0.01, *P < 0.05, ns = not significant).

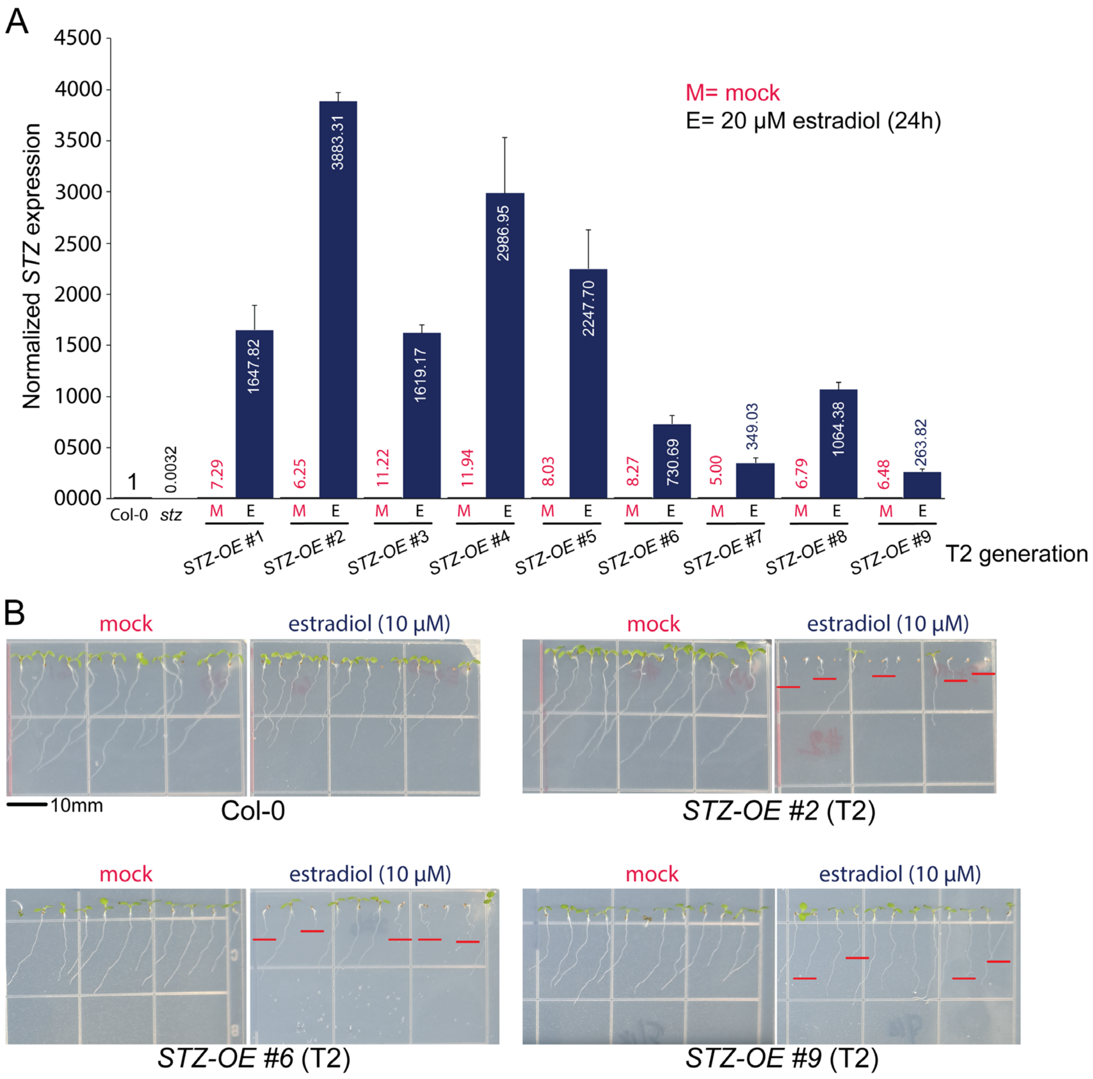

Supplementary Figure S9. Validation of *STZ-OE* transgenic lines (Supports Figure 3).

(**A**) Normalized *STZ* expression in T2 lines of *STZ-OE* seedlings validated by RT-qPCR. The T2 seedlings after BASTA selection were treated either with no estradiol (mock treatment) or estradiol (20 μM) for 1 d. RNA was extracted from whole seedlings to measure *STZ* transcript levels by RT-qPCR. The data are presented as ± SEM from three technical replicates. *GAPDH* was used as an internal control. (**B)** Representative images of selected T2 lines of *STZ-OE* (#2, #6, and #9) seedlings grown without estradiol (mock treatment; ½ MS medium) or on ½ MS medium containing 10 μM estradiol. Red lines indicate the ends of the roots. Since the seedlings were in the T2 generation, individuals with root growth suppression in response to estradiol segregated. The scale bars mentioned in B are applicable to all images under the same panel. *STZ-OE*, *STZ* overexpression line.

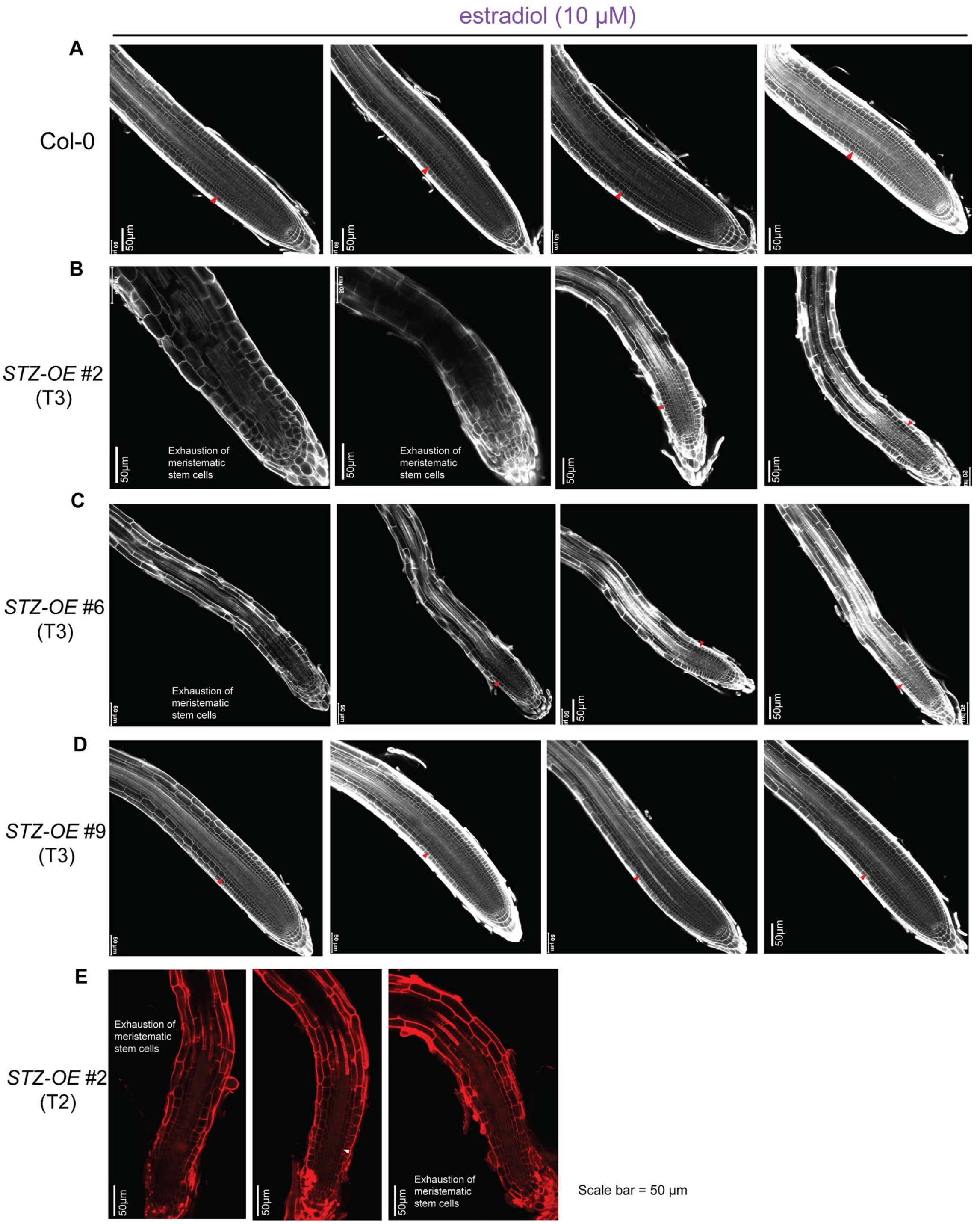

Supplementary Figure S10. Meristem images of *STZ-OE* transgenic lines expressing different dosages of *STZ* (Supports Figure 3).

(A-E) Confocal microscopy of cleared meristems of Col-0 or *STZ-OE* 5 DAT seedlings grown on ½ MS medium containing 10 μM estradiol. Similar to Col-0, *STZ-OE* #9 seedlings exhibited

proliferative meristem cells, whereas in *STZ-OE* #2, *STZ-OE* #6, and *STZ-OE* #2 seedlings (T2 generation), the proliferative meristem cells appear to be exhausted. The red or yellow arrowheads indicate the junction between the meristem and elongation zone. Notably, the differentiated protoxylem strands were proximal to the tips of *STZ-OE* #2 seedling roots. Scale bar = 50 μm (applies to all images in panels A-E). *STZ-OE*, *STZ* overexpression line; DAT = Days After Transfer to the growth chamber.

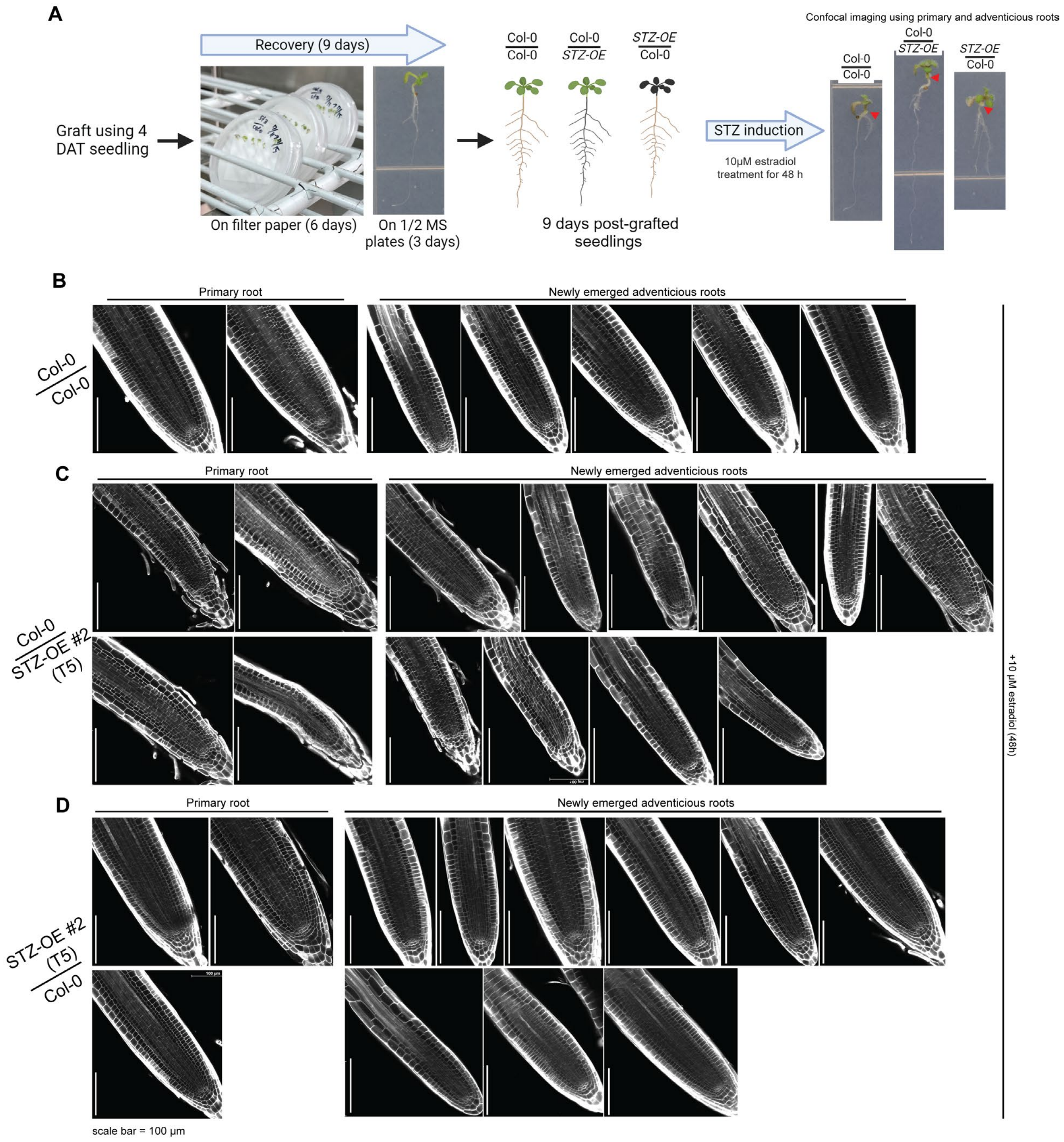

Supplementary Figure S11. *STZ* overexpression locally suppresses cell division at the root meristem (Supports Figure 3).

(A) Schematic overview of the grafting experiment. We used WT Col-0 and *STZ-OE* #2 seedlings at 4 DAT for grafting. The reciprocal grafts were generated and given 9 d for recovery before they were treated with 10 μM estradiol for 48 h. Red arrowheads indicate graft junctions. Primary and newly emerged adventitious roots were imaged using a confocal microscope. (**B-D**) Confocal

images represent the grafted seedlings' primary and newly emerged adventitious roots treated with estradiol. Col-0 scions grafted onto Col-0 rootstocks (Col-0/Col-0) were used as experimental controls. “Col-0/*STZ-OE* #2 (T5)” indicates that Col-0 scions were grafted onto *STZ-OE* #2 (T5 generation) rootstocks, and “*STZ-OE* #2 (T5)/Col-0” indicates that *STZ-OE* #2 (T5 generation) scions were grafted onto Col-0 rootstocks. Scale bar = 100 μm (applies to all images under panel B-D). *STZ-OE*, *STZ* overexpression line; DAT = Days After Transfer to the growth chamber.

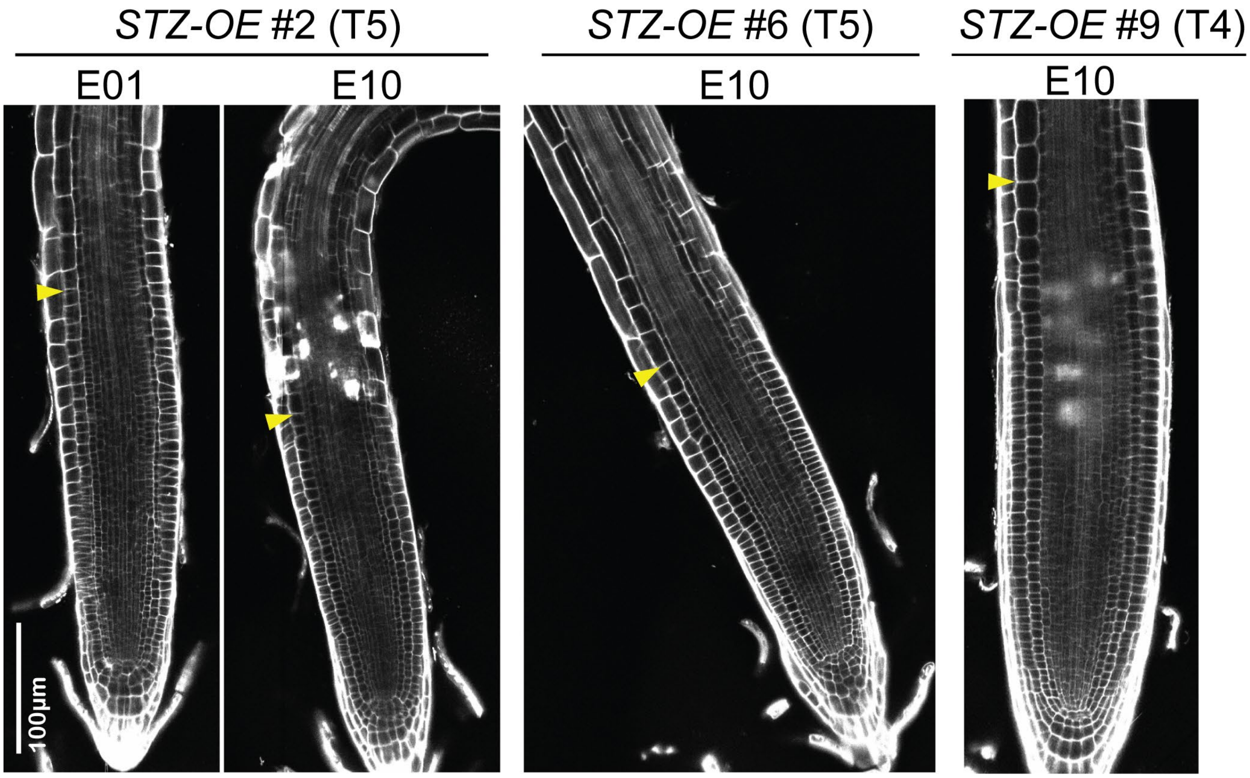

Supplementary Figure S12. The root apical growth of *STZ-OE* seedlings in response to estradiol treatment for 24 h (Supports Figure 3).

Seedling roots at 4 DAT were treated with estradiol (1 μM; E01 or 10 μM; E10) for 24 h. Confocal imaging of the meristems was performed 24 h after estradiol treatment. The yellow arrowheads indicate the junction between the meristem and elongation zone. Scale bar = 100 μm (applicable to all images presented). *STZ-OE*, *STZ* overexpression line; DAT = Days After Transfer to the growth chamber.

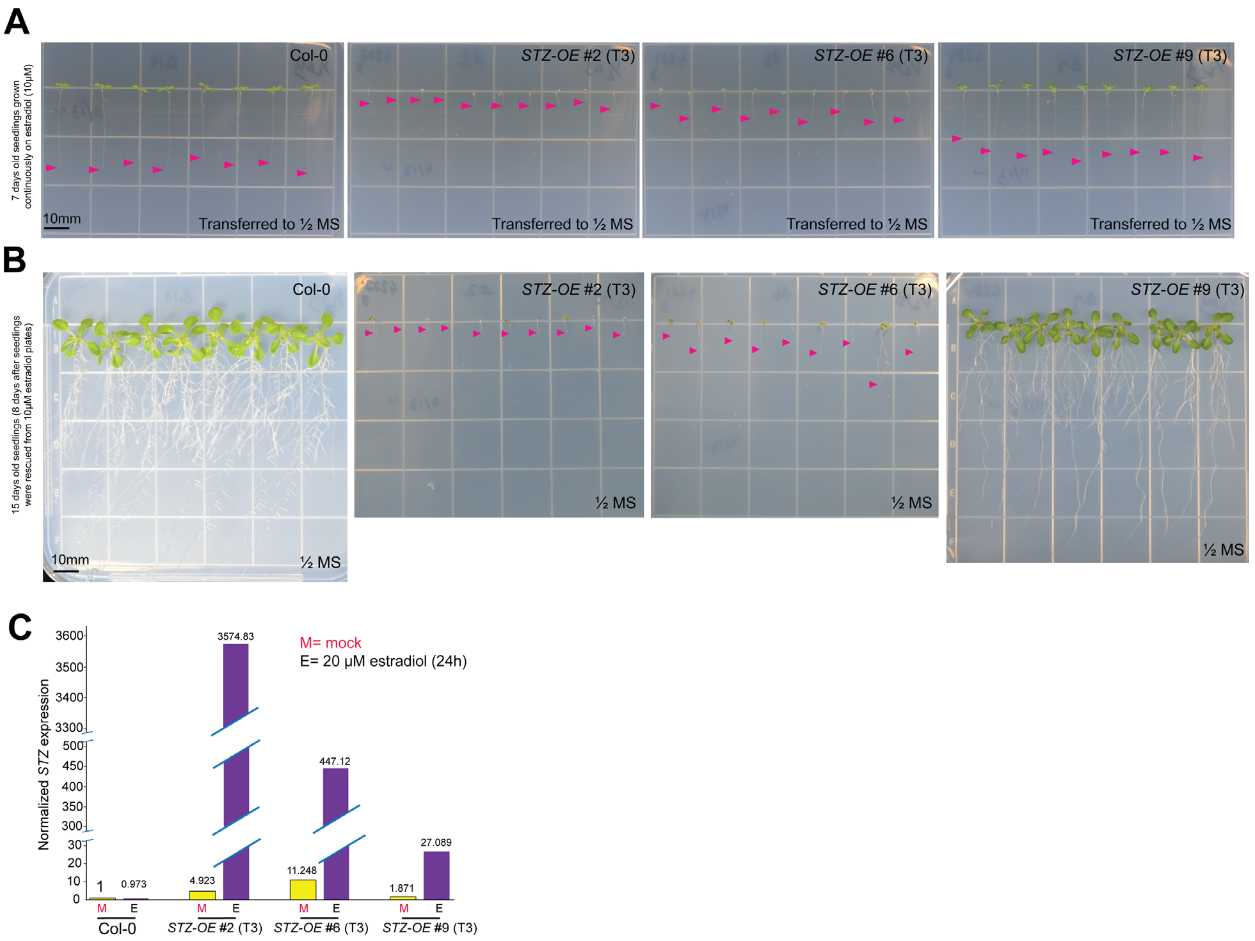

Supplementary Figure S13. Root growth rescue test of the *STZ-OE* seedlings (Supports Figure 4).

(A) Seven DAT Col-0, *STZ-OE* #2, *STZ-OE* #6, and *STZ-OE* #9 seedlings continuously grown on estradiol (10 µM) containing ½ MS medium, were transferred to ½ MS plates without estradiol for growth recovery. Scale bar = 10 mm (applies to all images presented) **(B)** These recovery plates were photographed 8 d after recovery. The pink arrowheads indicate the root tips. Scale bar = 10 mm (applies to all images presented). (**C**) The expression levels of *STZ* in *STZ-OE* seedling lines after 24 h of estradiol treatment were analyzed by RT-qPCR. Four DAT seedlings were treated with or without (mock treatment with ethanol) estradiol for 24 h, and the roots were dissected to isolate total RNA. The mock-treated Col-0 sample was used as a control to determine the fold change. The average expression values from three technical replicates were normalized by *GAPDH* and presented. *STZ-OE*, *STZ* overexpression line; DAT = Days After Transfer to the growth chamber.

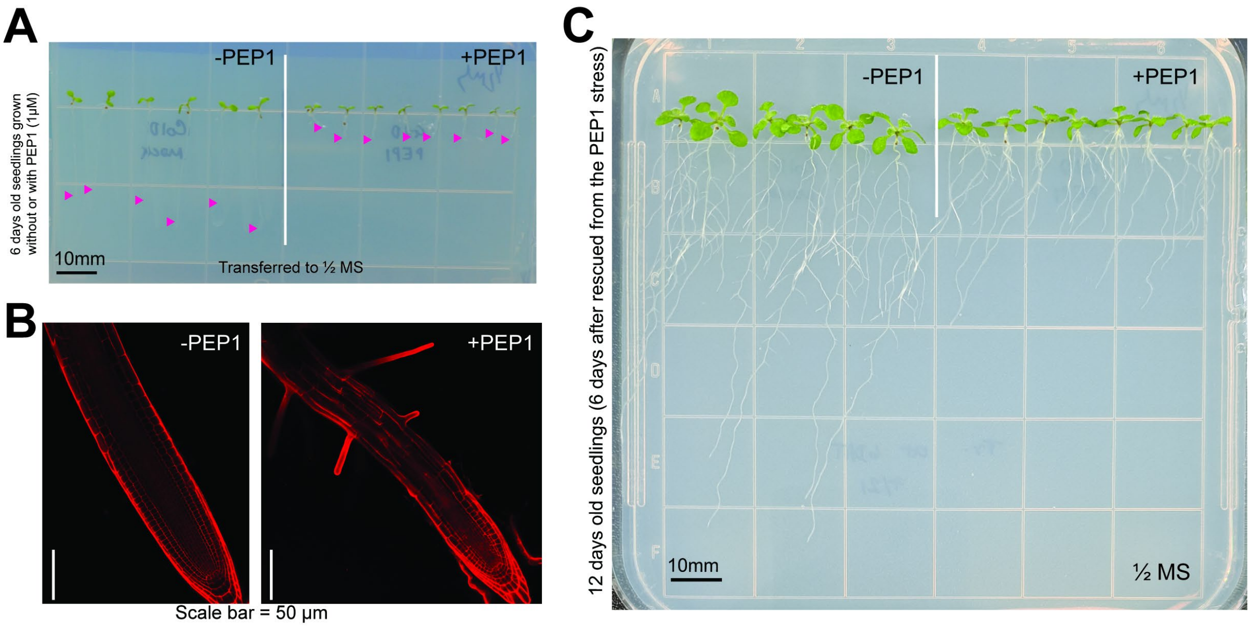

Supplementary Figure S14. Root growth rescue test using PEP1 treated Col-0 seedlings (Supports Figure 4).

(A) Col-0 seedlings at 6 DAT were germinated and grown continuously with or without 1 µM PEP1 on plates containing ½ MS medium and then transferred to a new ½ MS plate without PEP1 for growth recovery. Pink arrowheads indicate the root tips. (**B)** Representative images of the meristems of Col-0 seedlings grown with (+PEP1) or without PEP1 (-PEP1) for 6 d as shown in panel A. (**C)** The recovery plate mentioned in panel A was photographed 6 d after recovery. DAT = Days After Transfer to the growth chamber.

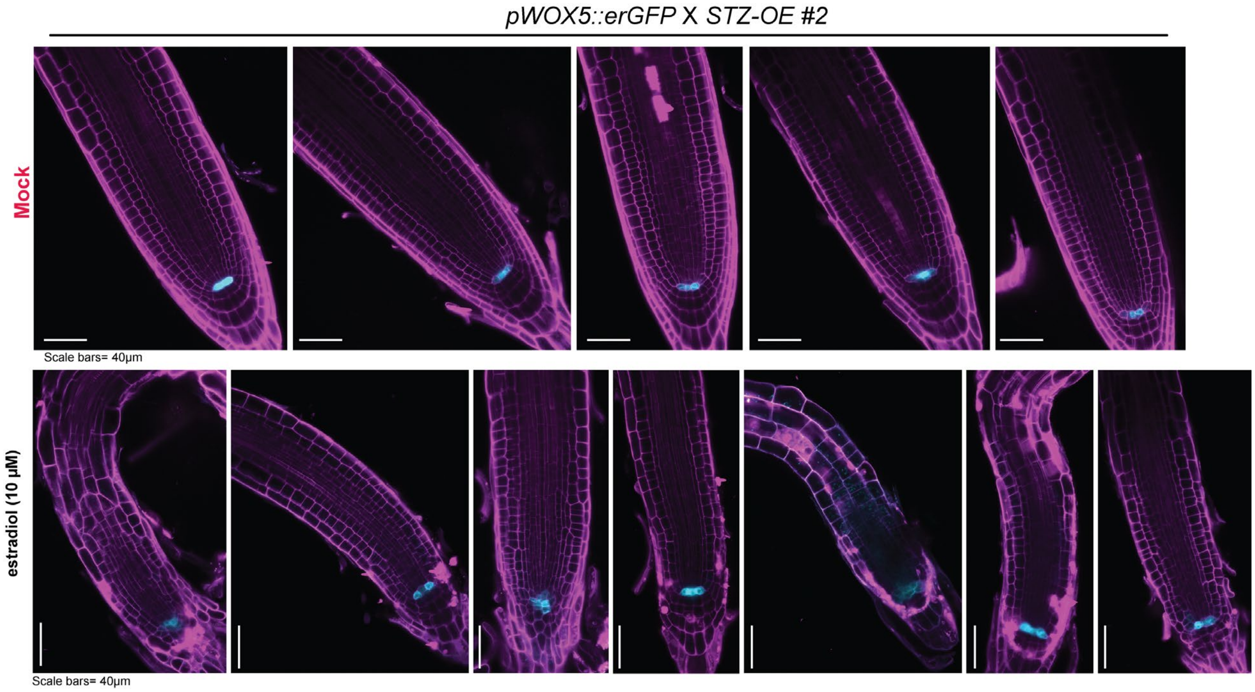

Supplementary Figure S15. Overexpression of *STZ* deforms QC identity (Supports Figure 4).

The *pWOX5::erGFP* signal was monitored in the F2 seedlings from a cross between *pWOX5::erGFP* and *STZ-OE* #2 plants. When grown on mock medium (½ MS medium plates supplemented with an equal volume of ethanol used for estradiol), the *pWOX5::erGFP* signal was specific to the QC. However, F2 seedlings with the "stunted-root" growth phenotype on estradiol (10 µM) plates exhibited a wide range of *pWOX5::erGFP* domains, including expansion and suppression of expression in the QC position. GFP: cyan; PI: magenta. Scale bar = 40 µm (applies to all images presented). *STZ-OE*, *STZ* overexpression line.

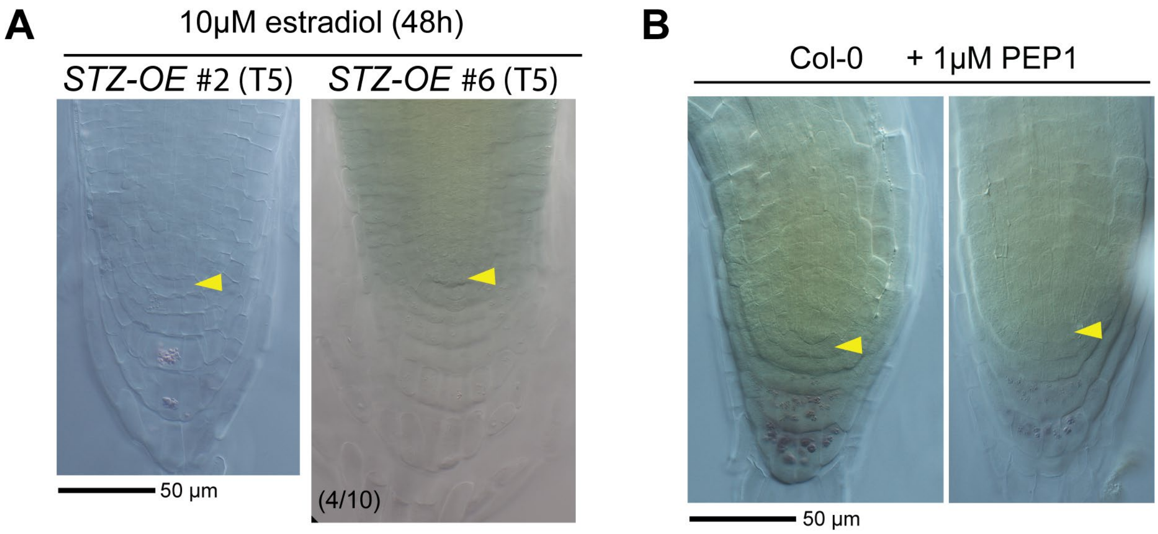

Supplementary Figure S16. Additional images of the Lugol's stained root tips of *STZ-OE* and PEP1 treated Col-0 seedlings (Supports Figure 4).

(A) Lugol's-stained root tips of *STZ-OE* #2 and #6 seedlings treated with estradiol for 48 h. The numbers in parentheses of the right panel indicate the number of samples with phenotypes similar to the representative image among all independent root samples analyzed. (**B)** Lugol's-stained root tips of Col-0 seedlings treated with PEP1 for 48 h. We used *STZ-OE* or Col-0 seedlings at 3 DAT grown on ½ MS medium and treated them with estradiol- and PEP1-containing ½ MS medium plates, respectively, for 48 h. Yellow arrowheads indicate QC. Scale bar = 50 µm (applicable to all images presented in panels A and B). *STZ-OE*, *STZ* overexpression line. DAT = Days After Transfer to the growth chamber.

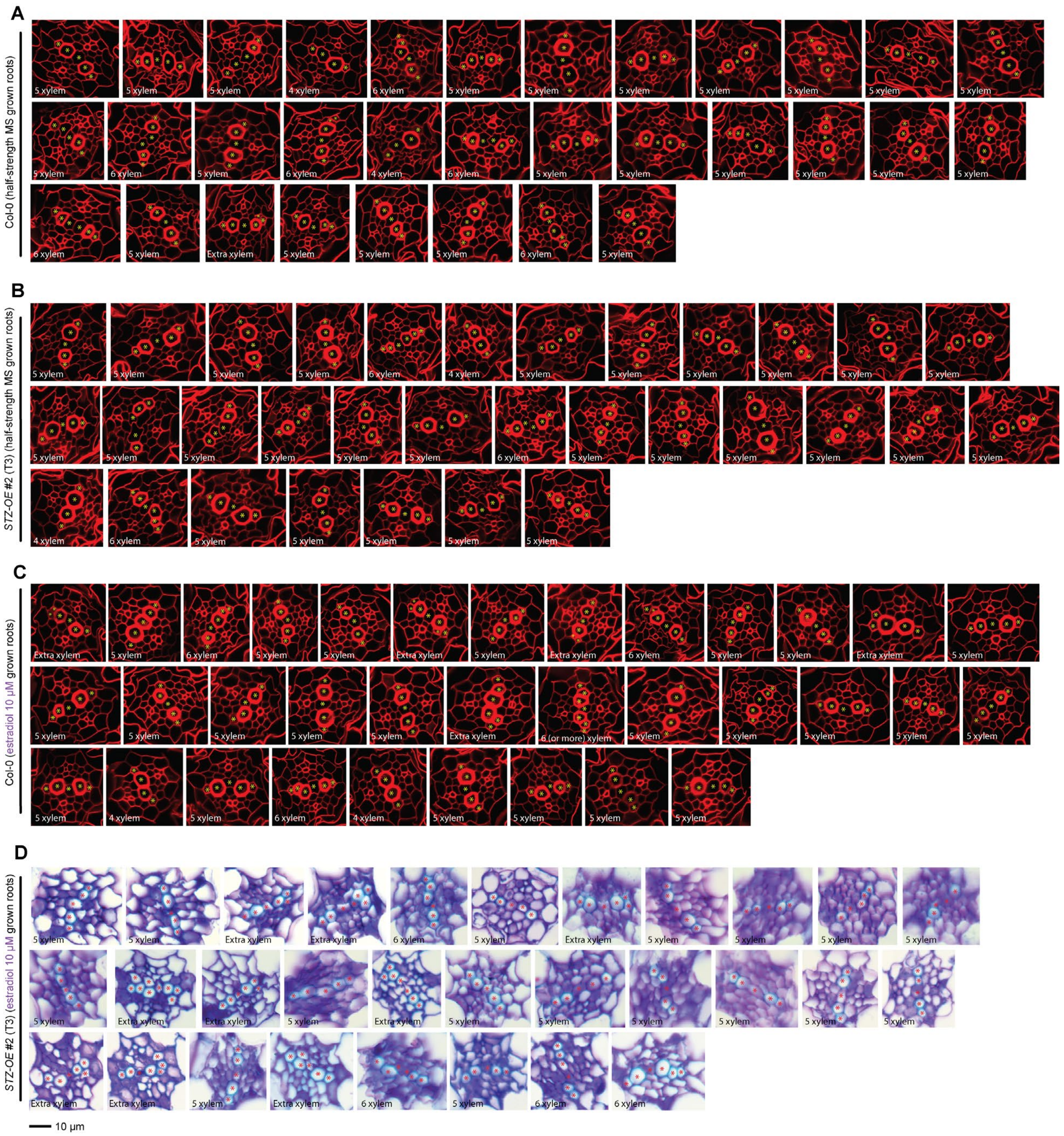

Supplementary Figure S17. The cross-sectional images of roots used to quantify xylem-vessel distribution phenotypes (Supports Figure 4).

(A-C) Vibratome root section images of Col-0 and *STZ-OE* #2 seedlings grown on ½ MS medium without estradiol and Col-0 seedlings grown on ½ MS medium with 10 μM estradiol. Sectioning was performed on 4–5 mm of the basal region from root tips. Yellow asterisks indicate xylem vessels. (**D)** Plastic sections from *STZ-OE* #2 seedling roots grown on ½ MS medium with 10 μM

estradiol. Sectioning (5-μm thick) was performed from 500 μm to 1 mm of the basal region of root tips. Red asterisks indicate differentiated xylem vessels. Scale bar = 10 μm (applicable to all images presented in panels A - D). *STZ-OE*, *STZ* overexpression line.

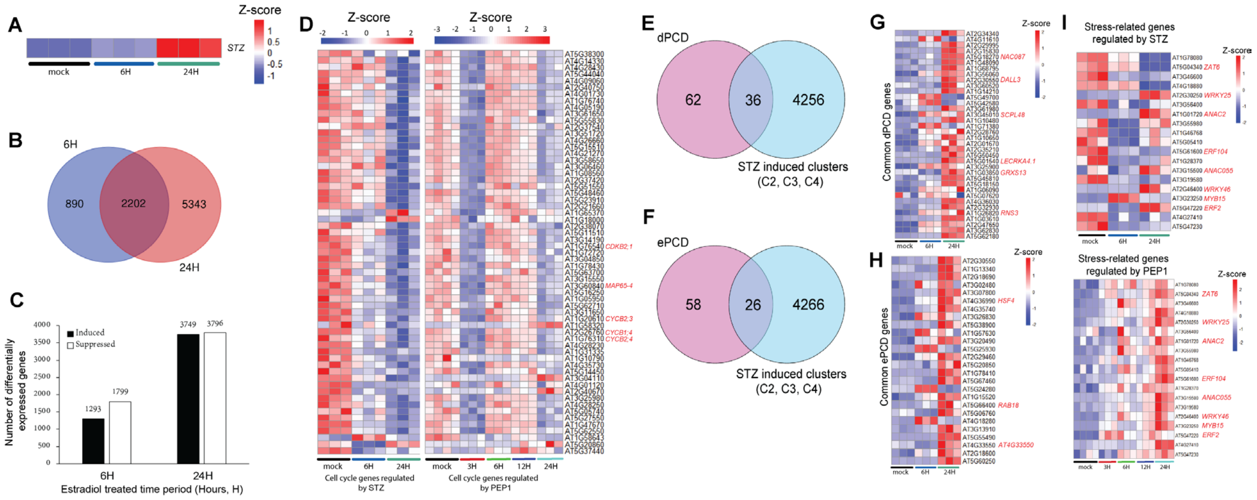

Supplementary Figure S18. STZ transcriptionally regulates cell cycle, dPCD, ePCD, and stress-related genes, as evidenced by the time-course RNA-seq experiment using *STZ-OE* seedling roots (Supports Figure 5).

(A) Heatmap representing the RPKM values for *STZ* transcripts at 6 h and 24 h after estradiol treatment. (**B)** A comparison of differentially expressed genes [DEGs; false discovery rate (FDR) < 0.01, |fold change| $\geq$ 1.5)] identified in *STZ-OE* RNA-seq analysis. Comparisons were made between DEGs found in root samples 6 h and 24 h after estradiol treatment. In total, 8435 unique DEGs were identified (listed in Supplementary Table S15). (**C)** The bar graph shows the numbers of induced and suppressed DEGs in the *STZ-OE* RNA-seq experiment. (**D)** Heatmap showing expression profiles of 61 commonly suppressed cell cycle genes following *STZ* overexpression or PEP1 treatment. (**E** and **F)** Venn diagram showing the common developmental and environmental programmed cell death (dPCD and ePCD) genes between DEGs in *STZ*-induced clusters (C2, C3, and C4) and PCD genes reported in other studies. The common dPCD and ePCD genes are listed in Supplementary Table S20. (**G** and **H)** Heatmap showing the expression profiles of common dPCD and ePCD DEGs in response to STZ overexpression. (**I)** Heatmap showing the transcriptional profiles of stress-responsive DEGs regulated by *STZ* overexpression and PEP1 treatment. The heatmaps were created using the "pheatmap" package in R. The values in all the heatmaps were row-normalized. The color scale bar denotes the Z-score as obtained through the "scale" function while plotting using the "pheatmap" package in R. Genes marked in red were used for RT-qPCR analysis. *STZ-OE*, *STZ* overexpression line.

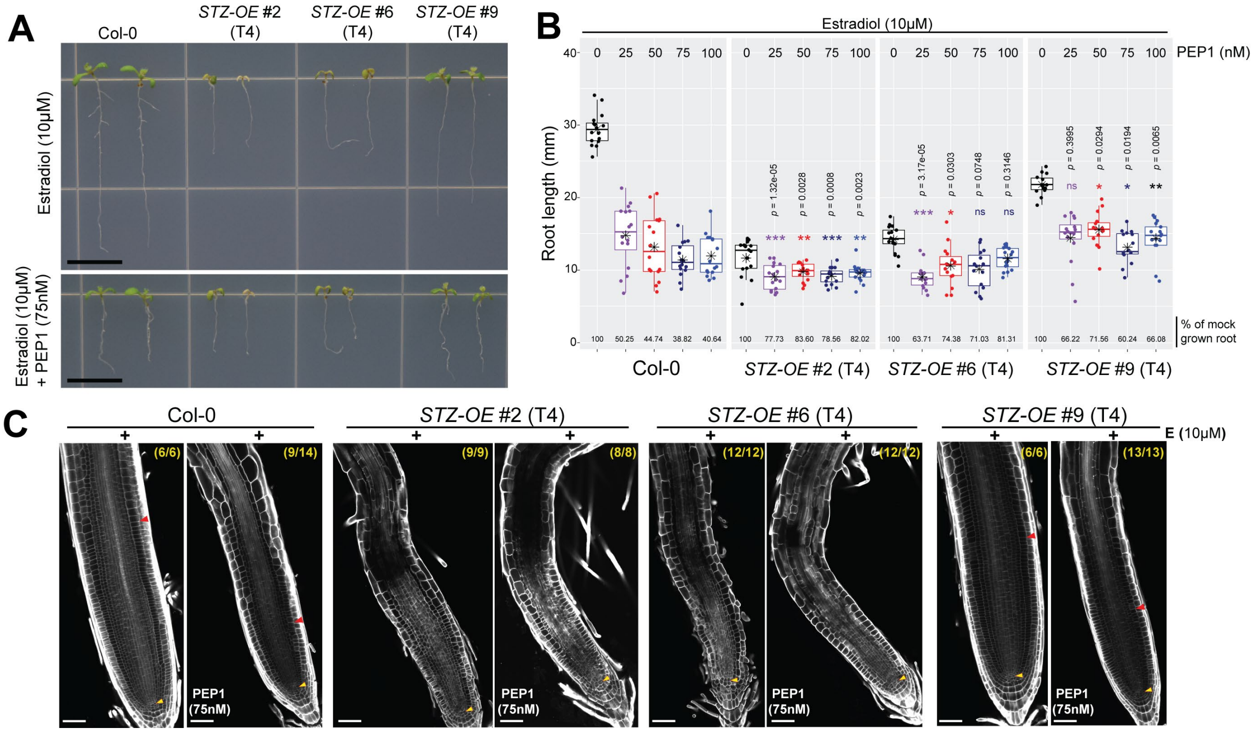

Supplementary Figure S19. Replicate analysis of the contribution of *STZ* dosages to the PEP1-induced root growth suppression (Supports Figure 7).

(A) Photographs of root growth of Col-0 and *STZ-OE* seedlings at 7 DAT grown in the presence of 10 μM estradiol and 75 nM PEP1 for 4 d. We used ½ MS grown Col-0 and *STZ-OE* seedlings at 3 DAT and grew them for another 4 d on ½ MS medium containing 75 nM PEP1 with 10 μM estradiol. The seedlings with representative root lengths were arranged on an agar plate and photographed. The *STZ-OE* seedlings used in this experiment were the T4 generation of *STZ-OE* lines #2, #6, and #9. Scale bar = 10 mm (applicable to both images under panel A). (**B)** The quantification of the root growth of Col-0 and *STZ-OE* seedlings at 7 DAT treated with estradiol (10 μM) and PEP1 at different doses (25, 50, 75, 100 nM) for 4 d as shown in panel A. Significant differences were determined by comparing WT Col-0 and *STZ-OE* seedlings grown under the same treatment conditions using a one-way Student's *t*-test (***P < 0.001, **P < 0.01, *P < 0.05; ns = non-significant). Boxes show the first and third quartiles split by median; whiskers indicate the range; points, individual data points; asterisks within the boxplot, average. Quantitative growth data, together with another biological replicate, are presented in Supplementary Table S25 (n = 14 - 17). (**C)** Meristem status of Col-0 and *STZ-OE* seedling lines under the conditions described in panel A. Yellow and red arrowheads indicate QC and meristem/transition zone boundaries, respectively. Numbers in parentheses in each panel indicate samples with similar results among

the total independent root samples analyzed. Scale bar = 30 µm (applicable to all the images under panel C). *STZ-OE*, *STZ* overexpression line; DAT = Days After Transfer to the growth chamber.

A

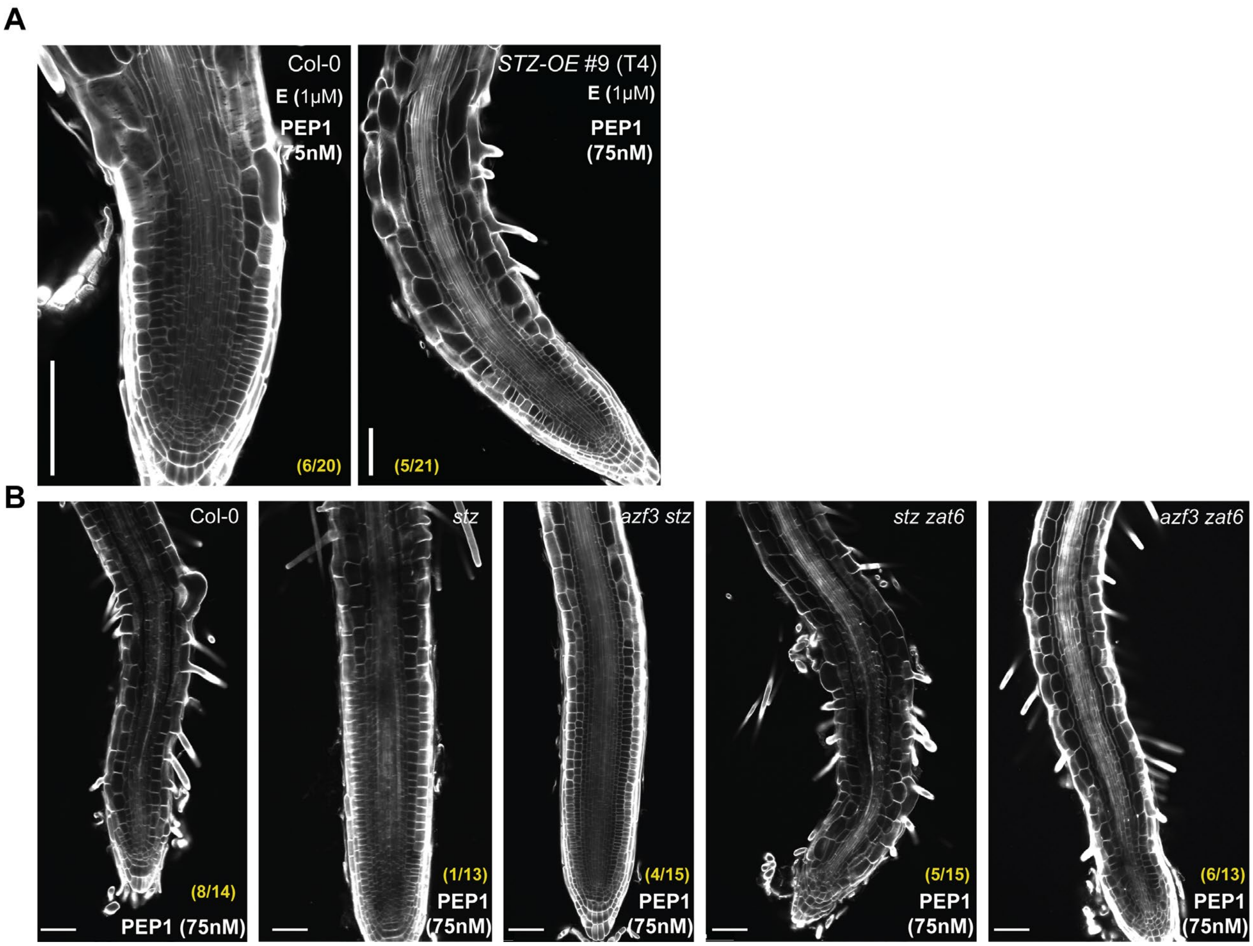

Supplementary Figure S20. Confocal microscopy showing additional root meristem phenotypes from *STZ-OE* and *stzl* seedlings grown in the presence of PEP1 (Supports Figure 7).

(A) Representative images of root meristems of Col-0 and *STZ-OE* #9 seedlings at 7 DAT grown in the presence of estradiol (1 μM) and PEP1 (75 nM) for 4 d. (**B)** Meristem images of Col-0 and *stzl* mutant seedlings at 7 DAT grown in the presence of PEP1 (75 nM) for 4 d. The numbers in parentheses in each panel indicate the number of samples with phenotypes similar to the representative image of all independent root samples analyzed. Scale bar = 30 μm (applicable to all the images).

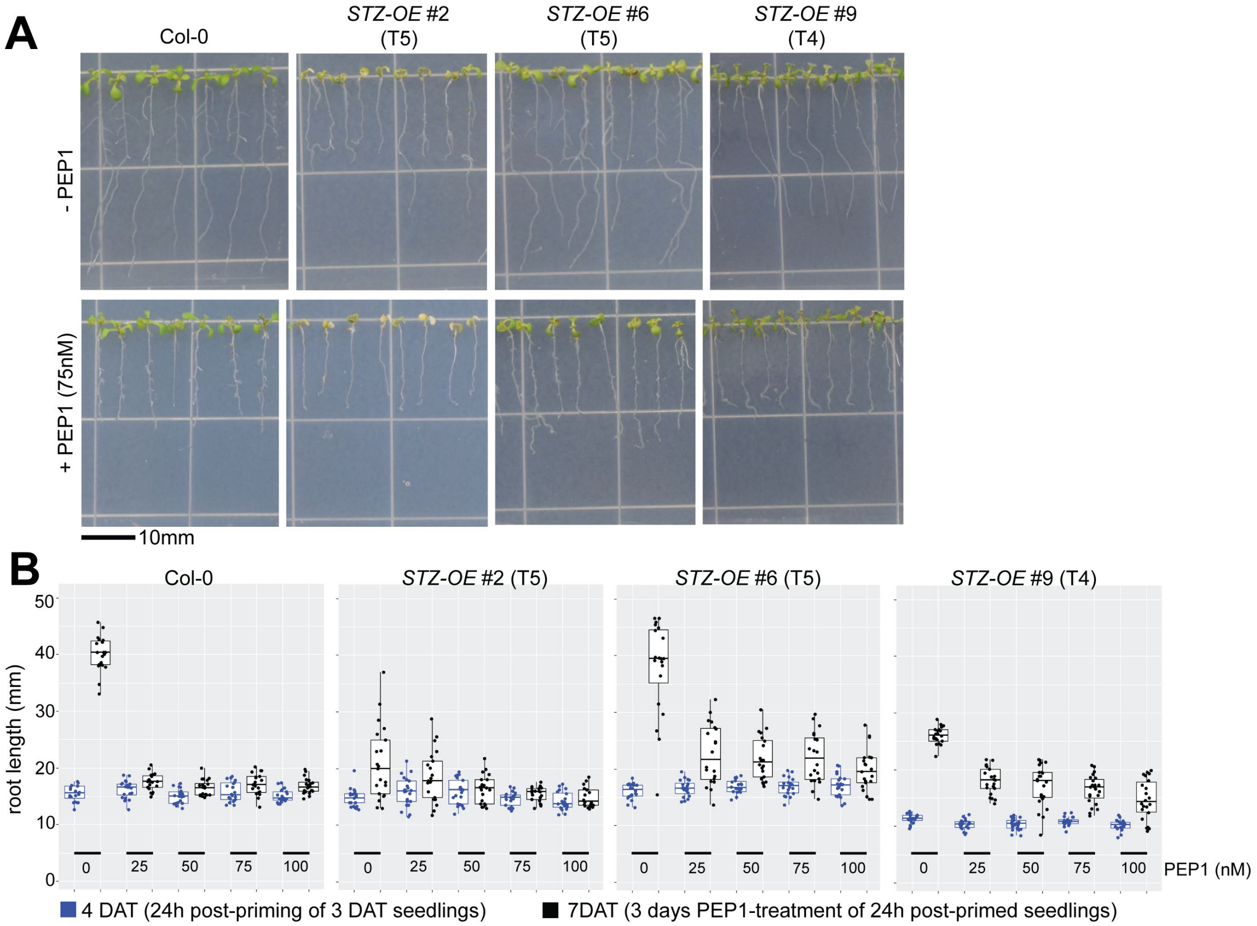

Supplementary Figure S21. Transient overexpression of *STZ* led to sustained root growth of *STZ-OE* seedlings under PEP1 treatment (Supports Figure 7).

(A) Representative images of root growth of Col-0 and *STZ-OE* seedlings at 7 DAT grown with 75 nM PEP1 for 3 d. We used ½ MS grown WT Col-0, *STZ-OE* #2, #6, and #9 seedlings at 3 DAT and transiently overexpressed (primed) *STZ* by adding liquid ½ MS medium containing 10 µM estradiol to the plate. After 24 h of transient overexpression (priming), we transferred the seedlings to ½ MS medium plates with or without PEP1 (75 nM). Scale bar = 10 mm (applicable to all photographs presented in panel A) (**B)** Quantification of root growth of seedlings in the post-primed (4 DAT) and post-PEP1 treatment (7 DAT) (Supplementary Table S25; n = 20 - 23). Boxes, the first and third quartiles split by median; whiskers, the data range; points, individual root lengths. Doses of 25, 50, 75, and 100 nM of PEP1 were used. *STZ-OE*, *STZ* overexpression line; DAT = Days After Transfer to the growth chamber.

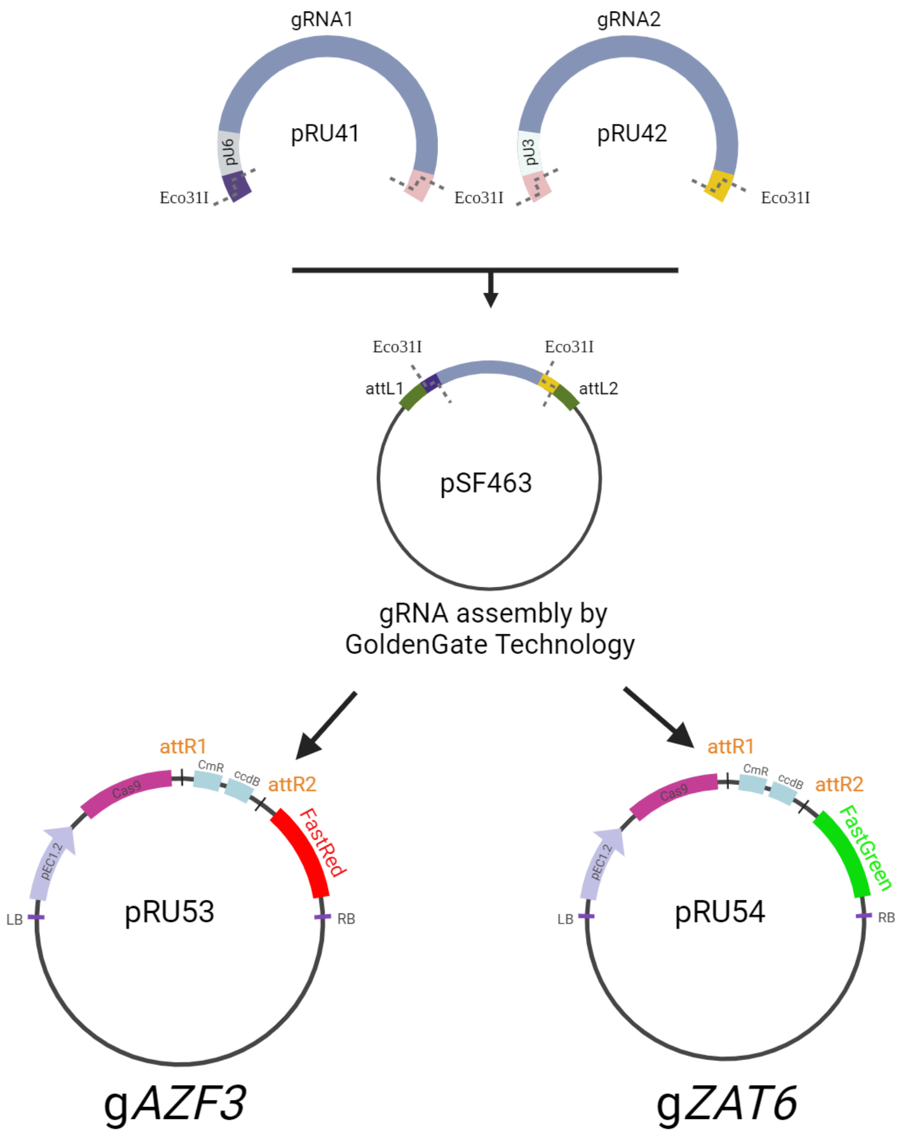

Supplementary Figure S22. Schematic map for guideRNA (gRNA) cloning (Supports Figure 7).

The target genes gRNA1 and gRNA2 were initially cloned into pRU41 and pRU42, respectively, and then recombined into a full cassette of the pSF463 vector through Golden gate assembly. The two gRNA-containing cassettes were then switched into gateway-compatible binary vectors, pRU53 (for *AZF3* gRNAs) and pRU54 (for *ZAT6* gRNAs) for plant transformation. See the Materials and Methods section for further details. The image was created with BioRender.com.

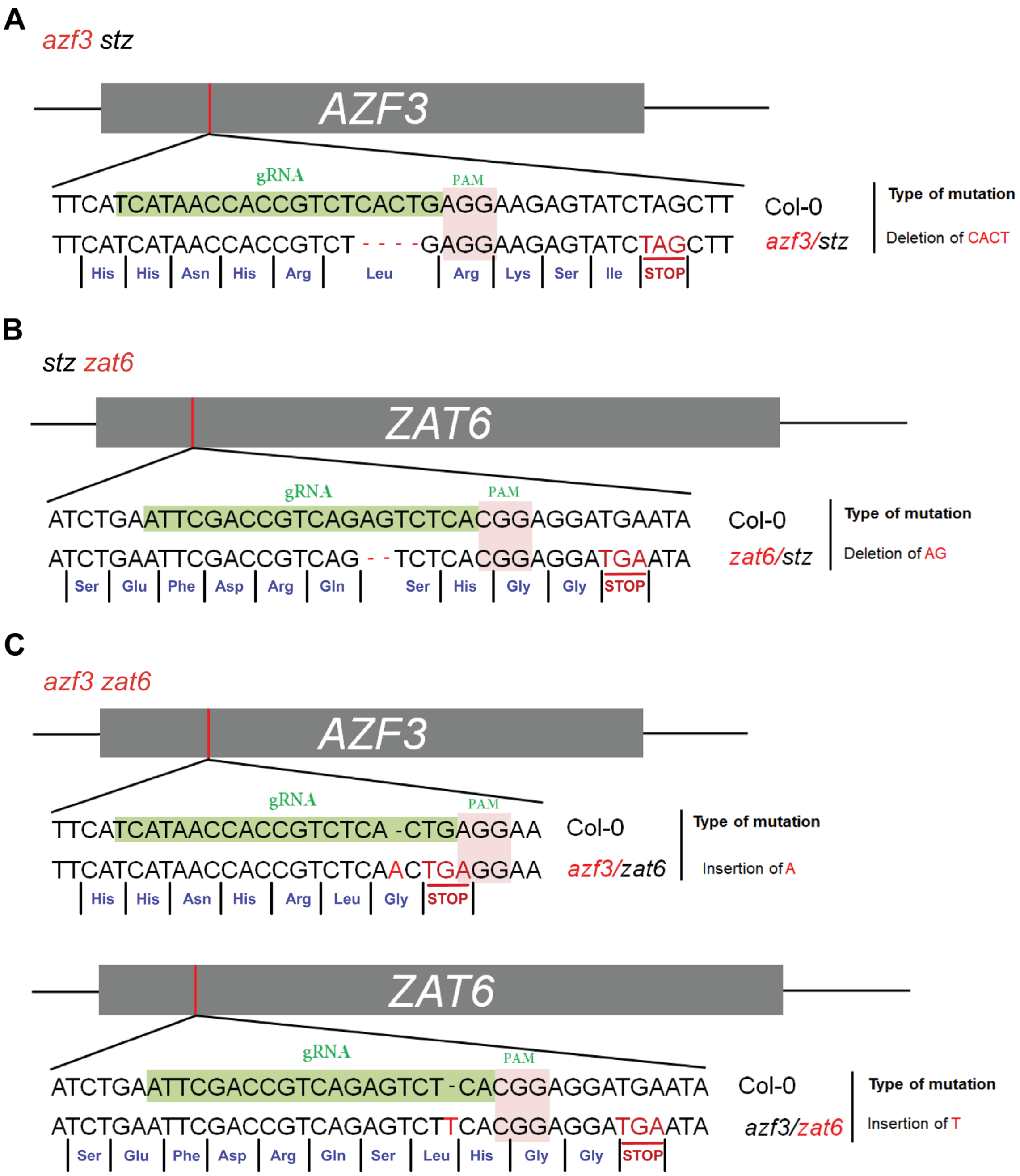

Supplementary Figure S23. Confirmation of CRISPR-Cas9 based mutation to generate *stzl* mutant combinations (Supports Figure 7).

(A) Sequencing result using the genomic DNA (gDNA) of *azf3 stz* double mutant seedlings confirms the deletion of “CACT” from the coding sequence of *AZF3*, which leads to the formation of a precocious stop codon. The background *stz* mutant line is SALK_054092. (**B)** The Sequencing result using the gDNA of the *stz zat6* double mutant seedlings confirms the deletion of "AG" from the coding sequence of *ZAT6*, which leads to the formation of a precocious stop

codon. The background *stz* mutant line for these double mutants is SALK_054092. (**C)** Sequencing result using the gDNA of *azf3 zat6* double mutant seedlings confirms an insertion of "A" into the coding sequence of *AZF3*, which leads to the formation of a precocious stop codon. For the *ZAT6* coding sequence, we observed an insertion of "T" in the gRNA, forming a precocious stop codon. The gDNA used for sequencing was obtained from the T3 generation seedlings. gRNA and PAM sites are highlighted in green and pink, respectively.
