# Supplementary material for: The molecular framework balancing growth and defense in response to plant elicitor peptide-induced signals in Arabidopsis
Source: Plant Cell. 2024 Dec 19;37(1):koae327. doi: 10.1093/plcell/koae327 (PMC11684079; doi:10.1093/plcell/koae327)
Supplement: koae327_Supplementary_Data [file tpc.00408.2024-s08.pdf › tpc.00408.2024-s08.pdf]

THE PLANT CELL

The molecular framework underlying the growth-defense tradeoff in Arabidopsis roots in response to danger signals

Souvik Dhar, Soo Youn Kim, Heeji Shin, Jongsung Park, and Ji-Young Lee

Review Timeline:

Submission Date:	26-Apr-2024
Editorial Decision:	21-Jun-2024
Revision Received:	24-Oct-2024
Accepted:	15-Nov-2024

1st Editorial Decision 21-Jun-2024

Prof. Ji-Young Lee
Seoul National University
Seoul 08826
Korea (South), Republic of

Dear Dr. Lee:

We have received reviews of your manuscript entitled "The molecular framework underlying the growth and defense tradeoff in Arabidopsis roots in response to danger signals." Thank you for submitting your best work to The Plant Cell. The editorial board agrees that the work you describe is substantive, falls within the scope of the journal, and may become acceptable for publication, pending revision and potential re-review.

We ask you to pay attention to the following points in preparing your revision:

1. Focus more on the novel role of the PEP1 in root development, particularly in the vasculature.

2. Strengthen the evidence for STZ's central role in the gene regulatory network. For example, overexpressing STZ in selected cell types rather than ubiquitous overexpression.

We should stress that we are reluctant to see manuscripts undergoing multiple rounds of revision and would be unlikely to offer you more than one chance to satisfy the reviewers.

A note from the Editor-in-Chief: We are trying to make a concerted effort to change green/red comparisons to green/magenta to make our figures understandable to those with color vision deficiencies. We noticed that a small number of your figures utilize red/green contrasts, so as you prepare the final version of the figures, please check the figures for red/green color use. Magenta is a good substitute for red, and a light blue substitutes for green even if the data are labeled as "RFP" or "mCherry" - readers will understand that colors can be changed, and indeed, it can be adjusted relatively quickly by using the "hue" setting in Photoshop or similar software. You might want to check over the Supplemental Figures for similar comparisons. Please note that color changes do not need to be highlighted or tracked in the revised manuscript, but could be noted in the cover letter or response document.

In addition, we find that the writing suffers from some problems with grammar and awkward phrasing, and in general lacks clarity and conciseness. We strongly encourage the use of a professional editing service to ensure that the work is presented in the best manner and reaches the broadest possible audience. Several editing services have agreed to extend a discount to authors coming to their website via this ASPB page https://aspb.org/aspb-journal-submission-editing-services/.

Please contact us if there are ambiguous comments or if you wish to discuss the revision.

Given the nature of the comments, we are offering you 60 days from when we have issued this decision to complete the revision. If a revision is not returned within this time frame, and if you have not been granted an extension, we will withdraw the manuscript, which will leave you free to submit the work elsewhere. If you need an extension, we encourage you to contact us at any point before the 60 days have passed.

When you are ready to submit the revised version, please upload a highlighted copy that indicates all changes made in response to the editor and reviewer recommendations. Include an itemized list of all changes made in response to each of the reviewer's suggestions in the "Response to Reviewers" section; please note that reviewers do not have access to your cover letter, nor was this decision letter shared with them.

Thank you for the privilege of reviewing your work. We look forward to receiving your revised manuscript.

On behalf of the editorial board,

Reviewing Editor, Board of Reviewing Editors
Siobhán Brady, Senior Editor
Blake Meyers, Editor-in-Chief

The Plant Cell

Please note the following:

-The Plant Cell now requires authors to complete and submit an author revisions checklist upon submission of a

revised manuscript. The aim of the checklist is to aid authors in preparing a high-quality manuscript, facilitate the review and assessment of revised manuscripts, and help to ensure that journal standards are maintained across the board. If your manuscript is accepted, the completed checklist will be published as supplemental material attached to the article online. Please download a copy of the checklist (pdf fillable form) at this link, for submission with your revised manuscript: https://tpc.msubmit.net/html/Author_Revisions_Checklist.pdf.

-Supplemental materials should be restricted to large datasets and tables, presentation of replicates, and validation of reagents, methods, or genotypes. Any data that are used to support the major claims must be in the main manuscript. Supplemental figure legends must indicate what figure in the main manuscript is supported by the supplemental data presented. Please justify how each of the supplemental figures meet the criteria.

-Sampling methods and nature of "biological replicates" should be described precisely (i.e. different plants, parts of plants, pooled tissue, independent pools of tissue, sampled at different times, etc), along with a clear description of and rationale for any statistical analyses conducted. The reader should know exactly what was sampled; what forms the basis of the calculation of any means and statistical parameters reported. This is also necessary to ensure that proper statistical analysis was conducted.

-Want to add this revision deadline to your calendar? Click below!

--- Reviewer comments:

Reviewer #1 (Comments for the Author):

Comments
The manuscript entitled: "The molecular framework underlying the growth and defense tradeoff in Arabidopsis roots in response to danger signals" describes the role of a plant elicitor peptide in controlling root growth.
Using RNAseq, the authors identified STZ and related genes as potential outputs for PEP1. They confirmed previous studies on the effect of PEP1 in controlling cell division in the vascular tissue and identified genes related to stress response in the PEP1-induced samples using a time course transcriptome from root tips. The identified genes were fused to fluorescent reporters and were used to check changes in their expression after the treatment with the peptide and confirm their induction and the tissue level.
While the transcriptomic and genetic data are solid, the link to the growth and defense tradeoff based on only one peptide remains weak. Rather than focusing the manuscript on the growth and defense, the authors could focus more on the novel role of the PEP1 in root development, particularly in the vasculature. Alternatively. the authors could perform pathogen assays in their mutants to test if their defense response is compromised after being challenged.
The manuscript will also benefit from a professional editing service as some sentences contain words that do not align with the meaning. I have highlighted some examples below.

Minor comments
In the abstract
Line 40: Change scheme with approaches or tools
Introduction
In lines 67-70, the authors quoted, " However, how PEP1 transcriptionally modulates root development and whether this is an indispensable part of the tradeoff between growth/development and stress resistance/defense, which we collectively call as 'the growth and defense tradeoff' are yet to be elucidated".
This sentence needs to be rephrased; the authors should indicate the logic behind establishing the role of PEP1 in the growth and defense trade. Is it because it is a peptide, and peptides are signaling molecules that might also have a role in stress response? It is important to provide the reasoning that supports the biological question.

The sentence on page 4, lines 88-89: " PEP1 rapidly induces the expression of genes encoding these TFs in the root apical meristem, including a stem cell niche." Please revise this sentence; as it is now, it reads like PEP1 induces a stem cell niche!

Line 90-91, ectopic vascular tissue differentiation, please rephrase correctly.
Line 95-97, the sentence:" These findings have the potential to guide sustainable crop plant growth under stress conditions, which might be possible by fine-tuning the expression of STZ and its homolog"
It is good to refer to potential use in the crop but maybe rephrase the sentence in a way that reflects the significance of the findings in understanding the role of peptides in controlling TF action in growth and defense; I think this is the major finding.
Figures 2D, 2E, and 2F, the pSTZ::GFP, pZAT6:: EGFP, and pAZF3::eGFP are only shown with 1uM PEP1; a control without the peptide should be shown similar to the GUS staining.

Figure 3D, it would be good to see if the columella stem cells are lost upon estradiol treatment.

Reviewer #2 (Comments for the Author):

In this manuscript, Dhar et al. provide a highly detailed transcriptome analysis of the Arabidopsis root meristem PEP1-associated danger response as well as a potential hypothesis for the long sought-after impact of PEP1 on plant growth arrest. The manuscript is well-written, and the claims largely supported by the data. Once reviewer comments are properly answered, I would support the publication of this manuscript in The Plant Cell. I believe that this manuscript is of significant interest to the field of plant danger peptide signaling.

Force of the paper:
Performing transcriptomic on the root meristem, rather than on whole seedlings (normally performed), provides a more relevant the impact of Pep1 on root physiology.

Major concerns:
The authors generated estradiol lines overexpressing STZ using the 35S promoter. While these lines provide insight into the consequence of expressing STZ to abnormally high degrees on plant physiology, it is not possible to speculate that the root phenotype observed by the authors may only be coming from root OE of STZ. The authors observed a 20-fold change difference in the expression of STZ in plants treated with PEP1 compared to mock at 1-hour post-treatment, while a >260-fold change difference in global STZ expression after EST treatment (line 9, which is the least OE competent line). Yet, they observe little-to-no meristem collapse in this line when treating with EST. Considering that STZ can activate PCD in the root, as the authors demonstrated, it is not possible to rule out that the phenotypes (root growth arrest and meristem collapse) they observe is caused by PCD activation in shoots as well, which leads to signal propagation in roots and therefore growth arrest. Considering the STZ is already quite well expressed in the mature stele in untreated conditions (Fig. 1).
Since it is unreasonable to ask the authors to create a new Arabidopsis line that would only be overexpressing STZ in certain root cell types under specific promoters and repeat all the experiments, we propose that the authors perform a grafting experiment with Col-0 WT (shoot) and the STZ lines (root) and provide data on the impact of EST induction in these grafts.

Minor concerns:
Related to the major concern: it would be interesting if the authors could provide a qPCR analysis of STZ expression in the EST-induced line 2-5 OE and compare it to the levels of STZ expressed in the meristem at a time point where a root meristem collapse phenotype occurs following a PEP1 treatment. Since line 2-5OE expresses STZ >260x more than non-induced line but doesn't show a clear root meristem collapse phenotype, it is critical to show that PEP1 triggers STZ to levels correlating that observed in OE lines in which meristem collapse occurs.
The experiment in Figure S9 is quite clever and interesting. Although I don't think it's necessary for publication, it would add good value to repeat this experiment with a Pep1 treatment. Can plants experiencing a meristem collapse following Pep1 recover from such an event?
The authors are using different terminologies for their different OE lines. For example: Line OE STZ 2-4, 2-5, 2-5-4, etc... This is true for other OE lines as well. This is very confusing to the reader. Are the lines 2-4, 2-5, 2-5-4 the same? If not, why changing from one line to another in between figures? Please justify and clarify in text/figures.
Figure S5E: The authors appeared to have only provided «merged» confocal microscopy images. Please provide each image deconvoluted for each field.

Editorial edits:
Line 51: Cited Huot et al., 2014 for growth-to-defense tradeoff review. It would be great if another, more recent review, would be chosen instead.
Line 250-251: I would recommend stating that no statistical difference was observed in STZ expression levels between pepr1/2 mock and treated plants, rather than «we could not observe STZ induction as high as in WT seedlings in response to 1hr PEP1 treatment (Figure S6c)». It would better represent the data.
Line 261-263: The authors write «We selected three STZ-OE lines (lines 2, 6, and 9 with 3883.3-, 730.7-, and 263.8-fold STZ expression compared to Col-0) having very high to relatively low induction levels of STZ expression». I believe that the term «relatively low» is not appropriate when there is >260-fold change difference in the expression of a gene that is clearly observable by GUS staining. Please, revisit the wording.

Line 372: Not actually «very high and moderately high», but rather very high and high levels of STZ. Remove the term

«moderately» as it is not accurate.

Line 509: ANAC055, not ANA055.

Figure5D: What is R1 and R2? Please provide the information in the figure legend.

Figure 6: Revisit the use of the word «moderate dosage of STZ» considering the other comments above.

All figure legends: Please, make sure to state what is the age of the plants at the moment of data acquisition in each figure. Also, make sure to mention for how long plants were subjected to each treatment in each figure. That will help the reader.

Reviewer #3 (Comments for the Author):

The manuscript by Dhar and colleagues from Ji-Young Lee's laboratory presents an interesting work detailing the transcriptome changes in roots of A. thaliana (hereafter Arabidopsis) upon the PEP1 elicitor treatment. The authors chose to use K-means clustering to group the DEGs into different categories according to their induction/repression kinetics over time along the 3-6-12-24-hour timepoints. Based on the PEP1-influenced TF network built upon the RNA-seq data, they started to focus on the C2H2 zinc finger TF family where STZ and other homologs belong to, with an aim to understand reveal the underlying mechanisms of growth-defence balance. Histological analysis of expression patterns of STZ members and analysis of RNA-seq data along with the existing tissue-specific root transcriptome datasets suggested that the these STZ members might play a key role in reshaping the growth pattern upon PEP1-perception, presumably in a tissue-specific way. Indeed, the authors included Figure 5 demonstrating that the STZ downstream genes are mainly enriched in cambium cell types. They attempted to mimic the effect or STZ TFs' induction using the three independent OE lines under the estradiol-induction system, which show varying degrees of induction of the STZ expression itself and growth defects. Using another round of RNA-seq, they addressed the transcriptome changes and revealed the specific downstream targets affected by the OE of STZ, demonstrating tissue-specific changes of different class of genes involved in distinct developmental and/or cell death regulation pathways. Using the OE lines as well as CRISPR-induced double KO lines, links between the mastery role of STZ TFs reorganizing growth patterns of root development were tested.

This work includes a fine-scale analysis of the select zinc finger TFs, STZ and its homologs, ZAT6 and AZF3 for tissue-specific localization upon induction and the report of marker xylem cell reduction in the STZ-OE line, suggesting the role of PEP1-induced regulatory network governed by STZ orchestrating growth and development potentially by tissue-specific manner. The transcriptome of the OE, especially enrichment of S18 for dPCD genes and S4 for cell cycle genes, along with xylem defects in the OE lines further corroborate the notion that STZ-pathway might operate to regulate overall root growth through the regulation of vasculature development. While the fine analysis of STZ-module tissue specificity is well presented as a strong merit of this work, data presented in Figure 5 as well as the usage of OE and its interpretation remain rather weak points of the work. This reviewer also has some doubts of central position of the STZ TF family in the gene regulatory (or interaction as the authors term) network based on current data presentation. Details on the data analysis and interpretation points that casted doubts on this reviewer are listed down below, so that the authors may use as points of improvements.

1. Starting with the network, it would be helpful for the authors reinforce the position of the STZ TF and its homologs in the network. Is the authors' intention to understand the PEP1-induced subnetwork topology, presumably with STZ TFs' making most connections, or to investigate link between the two subnetworks by breaking the connection between the PEP1 (stress)-induced network and cell cycle (growth-regulating) network? The latter seem most direct approach if the intention was to study the trade-off. Nonetheless, network robustness can be discussed and/or further analysed by network dispersal through taking out the target TFs one by one. How would the network topology change after taking out respective TFs belonging to the zinc finger family, at least STZ and ZAT6?
2. As the authors have PEP1-responsive transcriptome, they could evaluate the level of STZ expression in the OE lines shown in Figure2. Is the level in the OE acceptable or comparable to the PEP1-induced level in Arabidopsis? If the OE lines exceed the expression level by different orders of magnitude, what is the rational of using the OE lines? Possibly, the authors attempt to disturb the whole network governed by the STZ and its homologs as a collective output. Are there any known target sites of this class of TF? Discussion on the target site redundancy shared among the other TFs in the same family can be valuable in interpreting this data.
3. As the manuscript started with very nice fine-scale tissue specific analysis, this reviewer was expecting to see tissue-specific disturbance of the network, either OE or KO, although it might be quite challenging. It would be nice to have discussion or some additional data (if authors are already having it) detailing the mode of action of this regulatory network in the vasculature.
4. Figure 5 D, G tables completely bury raw data by showing relative ratios of growth reduction. Here what matters the most is the variation between the samples understandably as these data come from multiple treatment experiments

(especially OE with estradiol along with PEP treatment together). A box plot with individual datapoints all indicated will be highly desirable.
5. Regarding the interpretation of OE data presented in Figure 5, C-D, percentage reduction as a ratio upon PEP1 treatment used as a main value shall be reconsidered. STZ OE lines are already quite compromised with growth in the severe lines (#2 and #6) and their level of uptake of PEP1 would differ already. In addition, their effects in growth reduction in a ratio term obviously must differ as there must be a limit for plant root length to be reduced by a treatment. For the OE experiment, it would be advisable to increase the sample/replicate number "and" independent lines like #9 to further investigate the interesting possibility of slight overexpression (maybe physiologically closer to PEP1-treated samples) making the plants "less" susceptible to PEP1-induced growth defects.
6. The choice of STZ members (or other TFs) for KOs shall be reevaluated, which is related to the issue #1. It was stated in L175 that ZAT12 is another one highly connected in the network, if network topology or connectedness matters for this research. If the authors' choice of genes to follow up was based on rapid induction, Figure 2B indicates that At1g56110 might be an obvious choice, while STZ (first line) might not be super obvious. In the meanwhile, a qRT experiment within 1-hour treatment showed strongest induction in STZ. Any explanation on why there was a low STZ detection at 3 hours by RNA-seq but high induction in 1 hour by qRT for STZ? Overall, the authors seem to need to provide logical explanation regarding the choice of the genes selected for further investigation.

The following comments may help authors address specific issues that this reviewer stumbled upon in understanding.

Specific comments

• Figure 2A: Please indicate what colour of a node means. Several nodes were marked with yellow and orange, but there was no information to indicate what the colour-code means. Same applies to blue-coloured edges.

• Explain the logic behind the interaction in the NTW using STRING database, and discuss shortcomings and the advantages of using this method as to highlight the position of STZ in the network.

• L180-181: The abbreviation PlaCCI should appear right after the full description.

• L189: two "densely" connected? How do the authors know if the connection between the two subnetworks are densely connected? Any dispersal analysis is to be applied to test this dense connection or is this an syntax error of proposing two dense networks are sparsely connected by a handful key factors?

• L214-216: Figure 2 D-E: While the text says there was no expression "without PEP1 treatment", referring to Figure 2d and e, there seems to be a lack of explanation of ZAP6 GUS. In addition, while they claimed that there is no expression of eGFP without PEP1 treatment, data are not shown. The description should be revised to correctly explain the presented data.

• Figure 2 D-H: Is it a convention not to show (data not shown indicated in the Figure legend) confocal image without PEP treatment? As all these are images from new transgenic lines, it would be nice to show some images before treatment. For pAZF3 transcriptional fusion, the main text in L224 indicates specific expression in cortex and endodermis, but it is not clear if that also applies before the treatment given that the GUS expression is found. The authors shall be taking a careful approach to describe what has been shown and what has not been shown, as the main text description reads as if all the negative control was indeed shown. I note that the supplementary data has all the proper control indeed, and the text should be better pointing where the actual data locate.

• Figure 3A: The OE line number should be consistent. In A, the highest OE line number is indicated as #2-4 and all the rest indicates it as #2-5.

• L326-328: Is there any known work or literature supporting the link between xylem differentiation misregulation and dPCD? The link is loose in the current manuscript and this logical jump is not very well appreciable.

• STZ-OE: does it represent the situation where STZ and other homologs are systematically overexpressed (mimicking the situation of PEP treatment)?

• L347: more that > more than or more

• L343-344 What does it mean that dosages independently modulate? Does the authors mean that there is a threshold dividing the effects or dose-dependent gradual effects?

• The explanation on L369-377 should have appeared earlier when the different lines were introduced and to explain the rationale of qRT results.

• Figure 4e: L347-349: It is not clear what the percentage in the main text means; is it in terms of number of genes

affected or overall expression differences quantified? The heatmap presentation with the arbitrary values make it impossible for obtaining the generalized the number. This section requires better description.

• Figure 4D: S18-overrepresentation in dPCD is only pointed out in the Figure legend but not in the main text. Is there any reason why this description is underplayed, while the level of enrichment is quite outstanding and biologically seemingly relevant?

• L475-477: The RNA-seq data has 3-hour time point at its earliest. This description does not go with the data.

Dear Editor and Reviewers:

Thank you very much for your time and efforts in providing constructive review comments. Your comments immensely helped us improve this manuscript. We hope you find the submitted revision delivers the main scope of the manuscript more clearly and presents novel findings that could advance understanding of growth defense tradeoff mechanisms.

Multiple results and figures were restructured while adding new data and addressing review comments. Here is the list of significant changes:

1. The number of main figures increased from 6 to 8. This change aligns with restructuring the result sections from 5 to 7 sections. The last figure illustrating the molecular model is cited in the discussion.
2. Three major experiments were performed, and their results were included in the revision:
   1) In response to the comment by Reviewer #2, grafting between STZ-OE #2 and Col-0 was performed to examine whether the root meristem exhaustion in STZ -OE #2 is due to the locally induced STZ or due to the STZ induced in the shoot. Results are presented in Supplementary Figure S11.
   2) In response to the comment by Reviewers #2 and #3, transcript levels of STZ and its homologs in the root meristem required for suppressing meristem activities in STZ-OE and Col-0 treated with PEP1were analyzed using ddPCR. ddPCR results are presented in Figure 3E, and meristem images at the time of RNA isolation are presented in Supplementary Figure S12.
   3) In response to Reviewer #1, columella stem cells were analyzed for STZ-OE treated with estradiol and Col-0 treated with PEP1, using Lugol's staining. The data are presented in Figure 4B and Supplementary Figure S16.
3. Two revised experiments were performed, and their data were added: 1) time-course analysis of STZL transcriptional fusion lines using GUS staining, presented in Supplementary Figure S7. 2) Root growth of STZ-OE and stzl mutants in response to PEP1. All the raw measurement data are presented as boxplots in Figure 7 and Supplementary Figure S19.
4. Editage, a professional editing service, edited the revised manuscript's English. The certificate of editing was submitted as a supplemental document.

All the details of changes made in response to your comments are available in the point-by-point responses.

--

Reviewer #1 (Comments for the Author):

Comments
The manuscript entitled: "The molecular framework underlying the growth and defense tradeoff in Arabidopsis roots in response to danger signals" describes the role of a plant elicitor peptide in controlling root growth.

Using RNAseq, the authors identified STZ and related genes as potential outputs for PEP1. They confirmed previous studies on the effect of PEP1 in controlling cell division in the vascular tissue and identified genes related to stress response in the PEP1-induced samples using a time course transcriptome from root tips. The identified genes were fused to fluorescent reporters and were used to check changes in their expression after the treatment with the peptide and confirm their induction and the tissue level.

While the transcriptomic and genetic data are solid, the link to the growth and defense tradeoff based on only one peptide remains weak. Rather than focusing the manuscript on the growth and defense, the authors could focus more on the novel role of the PEP1 in root development, particularly in the vasculature. Alternatively, the authors could perform pathogen assays in their mutants to test if their defense response is compromised after being challenged.

Author response: Thank you for raising the point. In this manuscript, we aimed to establish STZ as a downstream of the PEP signaling pathway that controls the defense-growth tradeoff. Here, defense means resistance to a broad range of stressors that are not limited to biotic stressors. We judged that finding the role of STZ in a broad aspect of growth and defense should be done prior to finding the detailed regulation of vascular development at this point. Since we found a strong connection between STZ and several key regulators of vascular development, how this regulation affects the growth-defense tradeoff is our next question. In the revised manuscript, we included many additional data that we believe strengthen the critical points of this study and tried to address the most (if not all) comments you and other reviewers made.

The manuscript will also benefit from a professional editing service as some sentences contain words that do not align with the meaning. I have highlighted some examples below.

Author response: Thank you for your suggestion. To improve clarity, Editage provided a professional English editing service for the revised manuscript.

Minor comments

In the abstract

Line 40: Change scheme with approaches or tools

Author response: Thank you. Change was made.

Introduction

In lines 67-70, the authors quoted, " However, how PEP1 transcriptionally modulates root development and whether this is an indispensable part of the tradeoff between growth/development and stress resistance/defense, which we collectively call as 'the growth and defense tradeoff' are yet to be elucidated".

This sentence needs to be rephrased; the authors should indicate the logic behind establishing the role of PEP1 in the growth and defense trade. Is it because it is a peptide, and peptides are signaling molecules that might also have a role in stress response? It is important to provide the reasoning that supports the biological question.

Author response: Thank you for the comment. We modified the paragraph as follows in lines 58-68.

"Biotic and abiotic stress signals from the environment trigger the production of 'danger signals' in cells. Danger signals in the form of small metabolites or peptides are secreted outside the cell and sensed by neighbors, thereby activating stress responses. Plant elicitor peptides (PEPs) are endogenous danger signals that are produced in response to biotic and abiotic stressors and enhance stress tolerance (Huffaker et al., 2006; Huffaker et al., 2013; Ross et al., 2014; Yamada et al., 2016; Nakaminami et al., 2018; Hander et al., 2019). In Arabidopsis thaliana (hereafter Arabidopsis), synthetic PEP1 treated to seedlings suppresses apical root growth, triggers the formation of excessive root hairs, and alters the vascular tissue arrangement within the stele (Poncini et al., 2017; Dhar et al., 2021; Okada et al., 2021). However, how PEP transcriptionally modulates root development and whether this is a part of the growth-defense tradeoff remain to be elucidated."

The sentence on page 4, lines 88-89: " PEP1 rapidly induces the expression of genes encoding these TFs in the root apical meristem, including a stem cell niche." Please revise this sentence; as it is now, it reads like PEP1 induces a stem cell niche!

Author response: Thank you. The sentence was revised.

In lines 86-87, "PEP1 rapidly induces the expression of these TFs in root apical meristems."

Line 90-91, ectopic vascular tissue differentiation, please rephrase correctly.

Author response: Thank you. The sentence was revised.

In lines 87-89, "Transgenic roots overexpressing STZ phenocopied roots treated with PEP1, which had reduced meristem activity and expanded xylem differentiation domain."

Line 95-97, the sentence:" These findings have the potential to guide sustainable crop plant growth under stress conditions, which might be possible by fine-tuning the expression of STZ and its homolog" It is good to refer to potential use in the crop but maybe rephrase the sentence in a way that reflects the significance of the findings in understanding the role of peptides in controlling TF action in growth and defense; I think this is the major finding.

Author response: Thank you. The sentence was revised as follows in lines 93-96.

"These findings emphasize the significance of PEP signaling in controlling TF action to balance growth and defense and provide potential applications in sustainable crop plant growth under stressful conditions."

Figures 2D, 2E, and 2F, the pSTZ::GFP, pZAT6:: EGFP, and pAZF3::eGFP are only shown with 1uM PEP1; a control without the peptide should be shown similar to the GUS staining.

Author response: Thank you for your suggestion. The expression data of pSTZ::GFP, pZAT6:: EGFP, and pAZF3::eGFP without PEP1 treatment were added in Supplementary Figure S6.

Figure 3D, it would be good to see if the columella stem cells are lost upon estradiol treatment.

Author response: In this revision, we added Lugol's stained images of the stem cell niche of STZ-OE lines (3-day-old seedlings treated with 10µM estradiol for 48 hours) and the Col-0 seedlings treated with PEP1 (3-day-old seedlings treated with 1µM PEP1 for 48 hours). The result is described in lines 327-330.

Reviewer #2 (Comments for the Author):

In this manuscript, Dhar et al. provide a highly detailed transcriptome analysis of the Arabidopsis root meristem PEP1-associated danger response as well as a potential hypothesis for the long sought-after impact of PEP1 on plant growth arrest. The manuscript is well-written, and the claims largely supported by the data. Once reviewer comments are properly answered, I would support the publication of this manuscript in The Plant Cell. I believe that this manuscript is of significant interest to the field of plant danger peptide signaling.

Force of the paper:

Performing transcriptomic on the root meristem, rather than on whole seedlings (normally performed), provides a more relevant the impact of Pep1 on root physiology.

Thank you for your constructive comments on this manuscript. The revised manuscript addresses your concerns as follows.

Major concerns:

The authors generated estradiol lines overexpressing STZ using the 35S promoter. While these lines provide insight into the consequence of expressing STZ to abnormally high degrees on plant physiology, it is not possible to speculate that the root phenotype observed by the authors may only be coming from root OE of STZ.

1. The authors observed a 20-fold change difference in the expression of STZ in plants treated with PEP1 compared to mock at 1-hour post-treatment, while a >260-fold change difference in global STZ expression after EST treatment (line 9, which is the least OE competent line). Yet, they observe little-to-no meristem collapse in this line when treating with EST.

Author response: As you pointed out, we observed >20-fold induction of *STZ* in the root samples treated with PEP1 for 1 hour. For the STZ-OE line #9, >260-fold induction of *STZ* (as presented in Figure S9) was found from RNA extracted from BASTA selected whole seedlings. This discrepancy raised a question of the site of induction of STZ under the estradiol treatment and the involvement of STZ homologs other than STZ as a key player in the root meristem suppression.

To address these, we analyzed the numbers of transcripts of STZ and STZ homologs in the root meristems of STZ-OE seedlings treated with estradiol and Col-0 seedlings treated with PEP1 for 24 hours. We selected 24 hours for both 1µM PEP1 treatment and 1µM estradiol treatment because the reduction in root meristems was observed under these conditions (Supplementary Figure S1 and S12). We extracted total RNA from the

dissected meristems, synthesized cDNAs, and performed droplet digital PCR (ddPCR). The data are presented in Figure 3E. ddPCR results indicate that a very high dose of STZ in STZ-OE #2 rather leads to the suppression of other STZ homologs, ruling out that other STZ homologs suppress the root meristem. However, this ddPCR analysis does not answer why an extra high dosage of STZ is required for root meristem suppression in STZ-OE lines, unlike PEP1-treated roots. We speculate that PEP1 might regulate STZ at both transcriptional and post-transcriptional levels. This speculation is based on our observation of an unusually dim signal of STZ-GFP in the roots treated with PEP1. This could be due to the folding issue of STZ-GFP or the requirement of PEP1 signaling for STZ protein levels. In the follow-up study, we plan to explore the STZ regulation by PEP1 at protein levels.

2. Considering that STZ can activate PCD in the root, as the authors demonstrated, it is not possible to rule out that the phenotypes (root growth arrest and meristem collapse) they observe is caused by PCD activation in shoots as well, which leads to signal propagation in roots and therefore growth arrest. Considering the STZ is already quite well expressed in the mature stele in untreated conditions (Fig. 1). Since it is unreasonable to ask the authors to create a new Arabidopsis line that would only be overexpressing STZ in certain root cell types under specific promoters and repeat all the experiments, we propose that the authors perform a grafting experiment with Col-0 WT (shoot) and the STZ lines (root) and provide data on the impact of EST induction in these grafts.

Author response: As you suggested, we performed grafting experiments. The experimental scheme and data are presented in Supplementary Figure S11, and the results are described in lines 288-294. Among three types of grafting pairs (Col-0/Col-0, Col-0/STZ-OE #2, STZ-OE #2/Col-0), only STZ-OE #2 rootstocks grafted to Col-0 scions showed a reduction in the meristem under estradiol, suggesting a high dose of STZ locally suppresses meristem activities.

Minor concerns:

Related to the major concern: it would be interesting if the authors could provide a qPCR analysis of STZ expression in the EST-induced line 2-5 OE and compare it to the levels of STZ expressed in the meristem at a time point where a root meristem collapse phenotype occurs following a PEP1 treatment. Since line 2-5OE expresses STZ >260x more than non-induced line but doesn't show a clear root meristem collapse phenotype, it is critical to show that PEP1 triggers STZ to levels correlating that observed in OE lines in which meristem collapse occurs.

Author response: Thank you for your suggestions. This comment was addressed in the response to major concern #1.

The experiment in Figure S9 is quite clever and interesting. Although I don't think it's necessary for publication, it would add good value to repeat this experiment with a Pep1 treatment. Can plants experiencing a meristem collapse following Pep1 recover from such an event?

Author response: Thank you for the comment. We have added the rescue test results from the PEP1-treated experiment, as presented in Figure S14. Transferred seedlings from the (+) PEP1 media to the (-) PEP1 media enabled the regaining of root growth.

The authors are using different terminologies for their different OE lines. For example: Line OE STZ 2-4, 2-5, 2-5-4, etc... This is true for other OE lines as well. This is very confusing to the reader. Are the lines 2-4, 2-5, 2-5-4 the same? If not, why changing from one line to another in between figures? Please justify and clarify in text/figures.

Author response: Thank you for pointing this out. Lines 2-4 and 2-4-5 are the T3 and T4 generation from the same parental T2 line, #2. In the revision, we simplified the terminology by just adding T2, T3, T4, or T5 generation behind the original line number of independent T2 collections. For example, #2-4 to #2 T3, and #2-4-5 to #2 T4.

Figure S5E: The authors appeared to have only provided «merged» confocal microscopy images. Please provide each image deconvoluted for each field.

Author response: In the revision, we added the deconvoluted image for each field in Supplementary Figure S5E-G.

Editorial edits:

Line 51: Cited Huot et al., 2014 for growth-to-defense tradeoff review. It would be great if another, more recent review, would be chosen instead.

Author response: Thank you. We added one recent review article.

He, Z., Webster, S., and He, S.Y. (2022). Growth-defense trade-offs in plants. Curr. Biol. 32, R634–R639. **https://www.sciencedirect.com/science/article/pii/S0960982222007060**

Line 250-251: I would recommend stating that no statistical difference was observed in STZ expression levels between pepr1/2 mock and treated plants, rather than «we could not observe STZ induction as high as in WT seedlings in response to 1hr PEP1 treatment (Figure S6c)». It would better represent the data.

Author response: Thank you for your comment. A change was made as suggested.

In lines 262-263, **“**However, in the *pepr1 2* double mutant, no statistical difference was observed in STZ expression levels between mock and PEP1-treated plants (Figure S8c).”

Line 261-263: The authors write «We selected three STZ-OE lines (lines 2, 6, and 9 with 3883.3-, 730.7-, and 263.8-fold STZ expression compared to Col-0) having very high to relatively low induction levels of STZ expression». I believe that the term «relatively low» is not appropriate when there is >260-fold change difference in the expression of a gene that is clearly observable by GUS staining. Please, revisit the wording.

Author response: Thank you for pointing this out. The sentence was revised as follows.

In lines 273-276, “We selected three STZ-OE lines (lines #2, #6, and #9 with 3883.3-, 730.7-, and 263.8-fold STZ expression, respectively, compared with Col-0 seedlings), which had very high, high, and moderate induction levels of STZ expression.”

Line 372: Not actually «very high and moderately high», but rather very high and high levels of STZ. Remove the term «moderately» as it is not accurate.

Author response: Thank you for pointing this out. While restructuring the result section, we removed the whole paragraph that includes this expression.

Line 509: ANAC055, not ANA055.

Author response: Thank you. A correction was made.

Figure5D: What is R1 and R2? Please provide the information in the figure legend.

Author response: In the initial submission, R1 and R2 meant two independent experiments. In this revision, we revised the data presentation for Figure 5D (now 7B and E) following the comment by Reviewer 3. The updated figure presents the box plots with root lengths of individual seedlings for one experiment. The data for a repeated experiment are available in Table S25.

Figure 6: Revisit the use of the word «moderate dosage of STZ» considering the other comments above.

Author response: Thank you for pointing this out. The figure legend (Figure 8 in this revision) was revised as suggested.

All figure legends: Please, make sure to state what is the age of the plants at the moment of data acquisition in each figure. Also, make sure to mention for how long plants were subjected to each treatment in each figure. That will help the reader.

Author response: Thank you. As you suggested, we stated the age of the plants and the treatment time of PEP1 or estradiol in each figure legend. Those needing more details were described in the methods.

Reviewer #3 (Comments for the Author):

The manuscript by Dhar and colleagues from Ji-Young Lee's laboratory presents an interesting work detailing the transcriptome changes in roots of A. thaliana (hereafter Arabidopsis) upon the PEP1 elicitor treatment. The authors chose to use K-means clustering to group the DEGs into different categories according to their induction/repression kinetics over time along the 3-6-12-24-hour timepoints. Based on the PEP1-influenced TF network built upon the RNA-seq data, they started to focus on the

C2H2 zinc finger TF family where STZ and other homologs belong to, with an aim to understand reveal the underlying mechanisms of growth-defence balance. Histological analysis of expression patterns of STZ members and analysis of RNA-seq data along with the existing tissue-specific root transcriptome datasets suggested that the these STZ members might play a key role in reshaping the growth pattern upon PEP1-perception, presumably in a tissue-specific way. Indeed, the authors included Figure 5 demonstrating that the STZ downstream genes are mainly enriched in cambium cell types. They attempted to mimic the effect or STZ TFs' induction using the three independent OE lines under the estradiol-induction system, which show varying degrees of induction of the STZ expression itself and growth defects. Using another round of RNA-seq, they addressed the transcriptome changes and revealed the specific downstream targets affected by the OE of STZ, demonstrating tissue-specific changes of different class of genes involved in distinct developmental and/or cell death regulation pathways. Using the OE lines as well as CRISPR-induced double KO lines, links between the mastery role of STZ TFs reorganizing growth patterns of root development were tested.

This work includes a fine-scale analysis of the select zinc finger TFs, STZ and its homologs, ZAT6 and AZF3 for tissue-specific localization upon induction and the report of marker xylem cell reduction in the STZ-OE line, suggesting the role of PEP1-induced regulatory network governed by STZ orchestrating growth and development potentially by tissue-specific manner. The transcriptome of the OE, especially enrichment of S18 for dPCD genes and S4 for cell cycle genes, along with xylem defects in the OE lines further corroborate the notion that STZ-pathway might operate to regulate overall root growth through the regulation of vasculature development. While the fine analysis of STZ-module tissue specificity is well presented as a strong merit of this work, data presented in Figure 5 as well as the usage of OE and its interpretation remain rather weak points of the work. This reviewer also has some doubts of central position of the STZ TF family in the gene regulatory (or interaction as the authors term) network based on current data presentation. Details on the data analysis and interpretation points that casted doubts on this reviewer are listed down below, so that the authors may use as points of improvements.

1. Starting with the network, it would be helpful for the authors reinforce the position of the STZ TF and its homologs in the network. Is the authors' intention to understand the PEP1-induced subnetwork topology, presumably with STZ TFs' making most connections, or to investigate link between the two subnetworks by breaking the connection between the PEP1 (stress)-induced network and cell cycle (growth-regulating) network? The latter seem most direct approach if the intention was to study the trade-off. Nonetheless, network robustness can be discussed and/or further analysed by network dispersal through taking out the target TFs one by one. How would the network topology change after taking out respective TFs belonging to the zinc finger family, at least STZ and ZAT6?

Author response:

Thank you for your comment. Based on the time course PEP1 responsive transcriptome data, we primarily intended to find the candidates that link between the PEP1-induced stress network and the growth regulating network. We employed the network inferred from the STRING database to find these candidates, as presented in Figure 2. This network is based on the preexisting information, mainly relying on the cooccurrence of the nodes in text mining, as presented in Tables S9 and S10. Therefore, the edges between nodes do not reflect the *in vivo* connectivity. Because of the nature of this network, analyzing its network topology was not the logical step to follow. Instead, we selected candidates based on 1) the number of connections of candidate TFs in the network (degrees) and 2) the experimental evidence of their direct regulation of growth-related genes. Under

these criteria, STZ was the fourth TF with the highest degrees and the TF with the highest number of direct target genes involved in the cell cycle-related processes based on DAP-seq data. The rationale for selecting STZ and its homolog as candidates for this study was described with other experimental data in the second result section (Identification of transcription factors controlling the PEP signaling dependent reprogramming of root development) of the revised manuscript.

2. As the authors have PEP1-responsive transcriptome, they could evaluate the level of STZ expression in the OE lines shown in Figure 2. Is the level in the OE acceptable or comparable to the PEP1-induced level in Arabidopsis? If the OE lines exceed the expression level by different orders of magnitude, what is the rational of using the OE lines? Possibly, the authors attempt to disturb the whole network governed by the STZ and its homologs as a collective output. Are there any known target sites of this class of TF? Discussion on the target site redundancy shared among the other TFs in the same family can be valuable in interpreting this data.

Author response: Thank you for this comment.

While only about 20-fold induction of *STZ* was observed in the root meristems from Col-0 seedlings treated with PEP1, the STZ-OE line #9 showed more than 260-fold induction of *STZ* in the whole seedlings as presented in Figure S9. This discrepancy raised a question of the site of induction of STZ under the estradiol treatment and the involvement of STZ homologs other than STZ as a key player in root meristem suppression.

To address these, we analyzed the numbers of transcripts of STZ and STZ homologs in the root meristems of STZ-OE seedlings treated with estradiol and Col-0 seedlings treated with PEP1 for 24 hours. We selected 24 hours for both 1µM PEP1 treatment and 1µM estradiol treatment because root meristems were reduced under these conditions (Supplementary Figure S12). We extracted total RNA from the dissected meristems, synthesized cDNAs, and performed droplet digital PCR (ddPCR). The data are presented in Figure 3E.

ddPCR results indicate that very high STZ induction in STZ-OE leads to the suppression of other STZ homologs, ruling out the possibility that other STZ homologs suppress the root meristem. In STZ-OE line #9, the STZ level increased about 15 folds in the root meristem, which is still much higher than PEP1 treated roots. In this study, we could not answer why STZ-OE line #9 does not show a reduction in root meristem activities, though the STZ level is much higher than the one induced by PEP1. We speculate that STZ is regulated by PEP1 both transcriptionally and post-transcriptionally. This speculation is based on the comparison between translational and transcriptional fusion lines of STZ in our study. While the increase of transcriptional fusion GFP of STZ in response to PEP1 was very rapid, the signals of translational GFP of STZ were surprisingly low. This could be because the GFP fused to STZ has a problem being photoactivated. Alternatively, GFP-STZ could be posttranscriptionally (or posttranslationally) regulated depending on PEP1 signaling. In this scenario, STZ without PEP1 signals might require more *STZ* transcripts. We plan to explore these aspects in the follow-up study.

3. As the manuscript started with very nice fine-scale tissue specific analysis, this reviewer was expecting to see tissue-specific disturbance of the network, either OE or KO, although it might be quite challenging. It would be nice to have discussion or some additional data (if authors are already having it) detailing the mode of action of this regulatory network in the vasculature.

Author response: In the result section ‘Identification of downstream regulators of PEP-STZ pathway,’ we added the information about the direct targets of STZ among the TFs under the regulation of the PEP-STZ

pathway in lines 395-408. All the direct targets are presented in Supplementary Table S21. Several key TFs in vascular development were found and predicted to be direct targets of STZ. Whether STZ directly regulates these in vivo needs ChIP-seq data in the root. We are preparing this experiment as a follow-up to this study. Stele-specific STZ expression in the root meristem is also under development.

4. Figure 5 D, G tables completely bury raw data by showing relative ratios of growth reduction. Here what matters the most is the variation between the samples understandably as these data come from multiple treatment experiments (especially OE with estradiol along with PEP treatment together). A box plot with individual datapoints all indicated will be highly desirable.

Author response: Thank you for commenting on this. As you suggested, we generated boxplots with original root length data. Of the two replicate experiments, one is shown in Figure 7 (Figure 5 in the first submission) and the other in Supplementary Table S25. In Figure 7B, STZ-OE #9 shows longer roots than WT Col-0 under all the grades of PEP1. Contrary to overexpression lines, root growth suppression in the stz knockout mutant was more sensitive to PEP1 than the WT (Figure 7E). In this revision, we also included the data from priming STZ-OE before the PEP1 treatment (Supplementary Figure S20). Estradiol was treated to 3DAT seedlings for 24 hours, and then these seedlings were grown for 3 more days on the ½ MS media without estradiol but supplemented with varying concentrations of PEP1. After the priming, #6 and #9 continued the root growth in the presence of PEP1 while WT could not. These indicate that STZ confers resistance to PEP1 in a dose-dependent manner.

5. Regarding the interpretation of OE data presented in Figure 5, C-D, percentage reduction as a ratio upon PEP1 treatment used as a main value shall be reconsidered. STZ OE lines are already quite compromised with growth in the severe lines (#2 and #6) and their level of uptake of PEP1 would differ already. In addition, their effects in growth reduction in a ratio term obviously must differ as there must be a limit for plant root length to be reduced by a treatment. For the OE experiment, it would be advisable to increase the sample/replicate number "and" independent lines like #9 to further investigate the interesting possibility of slight overexpression (maybe physiologically closer to PEP1-treated samples) making the plants "less" susceptible to PEP1-induced growth defects.

Author response: This comment is related to the comment #4. In the boxplots presented in Figure 7, actual root lengths for all the individuals analyzed are shown. Results from a replicate experiment for STZ-OE lines are presented in Supplementary Table S25. In both experiments, line #9 showed superior root growth to the Col-0 under PEP1 treatment.

6. The choice of STZ members (or other TFs) for KOs shall be reevaluated, which is related to the issue #1. It was stated in L175 that ZAT12 is another one highly connected in the network, if network topology or connectedness matters for this research. If the authors' choice of genes to follow up was based on rapid induction, Figure 2B indicates that At1g56110 might be an obvious choice, while STZ (first line) might not be super obvious. In the meanwhile, a qRT experiment within 1-hour treatment showed strongest induction in STZ. Any explanation on why there was a low STZ detection at 3 hours by RNA-seq but high induction in 1 hour by qRT for STZ? Overall, the authors seem to need to provide logical explanation regarding the choice of the genes selected for further investigation.

Author response: I hope you agree on the logic behind the network-guided selection of candidate TFs that connect stress response and root growth (cell cycle regulation). At1g55110 (I guess you meant this gene) has nice dynamic expression changes in response to PEP1. However, the heatmap needs to be interpreted with

caution because expression values are row-normalized. The RPKM values of At1g55110 under PEP1 were much lower than STZ.

AGI	Mock3_3H	Mock4_6H	Mock2_12H	Mock1_24H	PEP1_3H_1	PEP1_3H_2	PEP1_3H_3	PEP1_6H_1	PEP1_6H_2	PEP1_6H_3	PEP1_12H_1	PEP1_12H_2	PEP1_12H_3	PEP1_24H_1	PEP1_24H_2	PEP1_24H_3
STZ	5.0	5.5	8.1	7.1	17.5	28.4	24.5	31.6	30.2	35.7	28.5	37.0	46.6	55.8	45.2	33.2
AT1G55110	2.1	2.4	2.2	1.5	5.1	5.6	6.1	3.8	4.2	4.3	3.0	4.2	4.5	6.3	4.6	3.9

The following comments may help authors address specific issues that this reviewer stumbled upon in understanding.

Specific comments

• Figure 2A: Please indicate what colour of a node means. Several nodes were marked with yellow and orange, but there was no information to indicate what the colour-code means. Same applies to blue-coloured edges.

Author response: Sorry for causing confusion. In the revised manuscript, the figure legend explained the information about the colored nodes and edges. The information about the edges is presented in Supplementary Table S10.

"The interaction scores are presented in Supplementary Table S10. Nodes highlighted in "red" represent the top five highly connected TFs among the 344 PEP1-induced TFs. The nodes highlighted in "yellow" represent the intermediary TFs from the most highly connected PEP1-induced TFs to the cell cycle cluster, based on the STRING database."

• Explain the logic behind the interaction in the NTW using STRING database, and discuss shortcomings and the advantages of using this method as to highlight the position of STZ in the network.

Author response: As described in response to comment #1, NTW using the STRING database was employed as a proxy to select candidate TFs potentially responsible for connecting the stress response network and growth-related cell cycle network. 8296 DEGs, yielded from the PEP1 responsive time-course data were too many to be used for building de-novo GRN. Since the STRING database combines rich information from literature, protein-protein interactions, and co-expression, it can provide linkages among functionally related genes. However, edges between genes could be misleading because these are not based on regulatory relationships but contextual linkage. Because of these pros and cons, we considered the STRING database as an *in-silico* guide to find candidates rather than as an actual network reflecting *in vivo* relationships.

• L180-181: The abbreviation PlaCCI should appear right after the full description.

Author response: Thank you. The correction was made as suggested.

• L189: two "densely" connected? How do the authors know if the connection between the two subnetworks are densely connected? Any dispersal analysis is to be applied to test this dense connection or is this an syntax error of proposing two dense networks are sparsely connected by a handful key factors?

Author response: We meant to emphasize the two functionally discrete subnetworks that were loosely connected to each other. Since this seems to cause confusion, we revised the sentence as follows:

In lines 188-191, "This *in silico* analysis revealed a large network made of 330 genes with 680 edges, which includes two subnetworks (Fig. 2A; Supplementary Table S10), one with a PEP1-induced TF cluster (Supplementary Fig. S5A) and the other with a distinct cell cycle gene cluster (Fig. 2A)."

• L214-216: Figure 2 D-E: While the text says there was no expression "without PEP1 treatment", referring to Figure 2d and e, there seems to be a lack of explanation of ZAP6 GUS. In addition, while they claimed that there is no expression of eGFP without PEP1 treatment, data are not shown. The description should be revised to correctly explain the presented data.

• Figure 2 D-H: Is it a convention not to show (data not shown indicated in the Figure legend) confocal image without PEP treatment? As all these are images from new transgenic lines, it would be nice to show some images before treatment. For pAZF3 transcriptional fusion, the main text in L224 indicates specific expression in cortex and endodermis, but it is not clear if that also applies before the treatment given that the GUS expression is found. The authors shall be taking a careful approach to describe what has been shown and what has not been shown, as the main text description reads as if all the negative control was indeed shown. I note that the supplementary data has all the proper control indeed, and the text should be better pointing where the actual data locate.

Author response: Following two comments on Figure 2, we added a new Supplementary Figure S6 that includes the confocal microscopy of transcriptional and translational fusion lines with and without PEP1 treatment. GFP signals in the region where we detected weak GUS signals are very dim without PEP1 treatment.

• Figure 3A: The OE line number should be consistent. In A, the highest OE line number is indicated as #2-4 and all the rest indicates it as #2-5.

Author response: This was because lines #2-4 and #2-5 are T3 siblings from #2 in T2 generation. Following the comments from you and Reviewer 2, we simplified the naming of OE lines by just adding T2, T3, T4, or T5 generation behind the original line number of independent T2 collections.

• L326-328: Is there any known work or literature supporting the link between xylem differentiation misregulation and dPCD? The link is loose in the current manuscript and this logical jump is not very well appreciable.

Author response: While revising the manuscript, we addressed the expression dynamics of cell cycle genes and dPCD genes sequentially with the intention of finding the anti-correlative expression changes of these two gene groups in lines 411-433. Studies on the functional connection between xylem differentiation and PCD were cited in the middle. We hope you find this revised paragraph logically acceptable.

• STZ-OE: does it represent the situation where STZ and other homologs are systematically overexpressed (mimicking the situation of PEP treatment)?

Author response: Yes, we intended to create the situation mimicking the PEP1-treated root by artificially overexpressing STZ. To avoid the ambiguity of this approach, we added the introductory sentence indicating our intention as follows:

In lines 267-268, "We then investigated whether STZ overexpression alone can recapitulate the PEP1 mediated root growth response. To this end, we developed STZ-overexpressing transgenic lines using a well-established estrogen-inducible XVE system (Zuo et al., 2000; Siligato et al., 2016). We transformed Col-0 plants with the vector carrying p35S::XVE>>pLexA::STZ (STZ-OE) transgene."

• L347: more that > more than or more

Author response: Thank you. The correction was made in line 362 in the revision.

• L343-344 What does it mean that dosages independently modulate? Does the authors mean that there is a threshold dividing the effects or dose-dependent gradual effects?

Author response: Sorry for the ambiguity of the wording. This comment and the next one made us rethink the way the results were presented. We decided to restructure the results, making a separate section about the relationship between STZ doses and its control of root growth and stress resistance. Please visit the section "STZ regulates stress response and root growth with a discrete threshold," which presents the qRT-PCR data of genes involved in cell cycle, PCD, and stress resistance.

• The explanation on L369-377 should have appeared earlier when the different lines were introduced and to explain the rationale of qRT results.

Author response: Thank you for commenting on this. We considered your comment and decided to make more drastic changes in describing qRT-PCR results. In the revised manuscript, we generated a separate result section "STZ regulates stress response and root growth with a discrete threshold". In this section, we compared the expression changes of the cell cycle, dPCD, ePCD, and stress regulators in STZ-OE #2 and #9. Regarding your next comment, we also changed the data presentation from heatmap to box plots. These data are presented in Figure 6. STZ-OE #2 suppresses cell cycle genes by 50% while upregulating dPCD, ePCD, and stress regulators. Contrary to #2, STZ-OE #9 did not show clear changes in majority of cell cycle, dPCD, and ePCD gene expression. Unlike growth-related genes, stress regulators were upregulated by STZ-OE #9. In line with the section title, these data indicate that the upregulation of stress regulators requires low doses of STZ while expression changes in growth-related genes (cell cycle, dPCD, and ePCD genes) require a high dose of STZ.

• Figure 4e: L347-349: It is not clear what the percentage in the main text means; is it in terms of number of genes affected or overall expression differences quantified? The heatmap presentation with the arbitrary values make it impossible for obtaining the generalized the number. This section requires better description.

Author response: As explained in the previous response, we changed the presentation of qRT-PCR data from heatmaps to bar graphs. Now, you can find the percentage of gene expression changes and their statistical significance in Figure 6. Thank you for commenting on this.

• Figure 4D: S18-overrepresentation in dPCD is only pointed out in the Figure legend but not in the main text. Is there any reason why this description is underplayed, while the level of enrichment is quite outstanding and biologically seemingly relevant?

Author response: Thank you for this comment. dPCD genes are mainly associated with the cell death program of xylem vessels; therefore, we focused more on the enrichment of these genes downstream of STZ rather than their expression patterns. However, several TFs important for vascular development are affected in their expression by STZ and PEP1. We did not emphasize this aspect well enough in the first submission. Therefore, we restructured the result sections and included the STZ regulation of multiple regulators of vascular development as described in lines 395-408.

• L475-477: The RNA-seq data has 3-hour time point at its earliest. This description does not go with the data.

Author response: Thank you for pointing this out. A correction was made.

Dear Dr. Ji-Young Lee:

We are pleased to inform you that your paper entitled "The molecular framework underlying the growth-defense tradeoff in Arabidopsis roots in response to danger signals" has been accepted for publication in The Plant Cell, pending a final editorial review by a science editor. At this stage, your manuscript will be evaluated by a science editor with respect to its presentation of scientific content, compliance with journal policies, and presentation for a broad readership. The Plant Cell has contracted with Plant Editors (planteditors.com) to provide this service to our authors, and you will soon receive additional information on this process.

Please note that each author needs to link their ORCID identifier to their account in the system before your manuscript can be published. If any authors do not have an ORCID linked to their account, they will receive a message with a link to complete this task. Please ensure that ALL of your coauthors have completed this task as soon as possible.

LICENSE INFORMATION Shortly after your paper is sent to production, you will receive an email from our publisher's author support team, **SciPris**, with information on article processing charges, as well as the types of Open Access license you would be able to purchase. The accepted version of your manuscript will not appear on our Advance Articles page until the license is selected and signed, so please look out for an email from SciPris. For more information see the Article Publication Fees section of our author guidelines: https://academic.oup.com/plcell/pages/General-Instructions

The Plant Cell and The Arabidopsis Information Resource (TAIR) are collaborating to collect functional annotation data about Arabidopsis genes from authors. This includes information about the gene's molecular function (e.g., kinase activity, ATP synthetase activity), the biological process/es it is involved in (e.g., endosperm development, threonine biosynthesis), its subcellular location (e.g., nucleus, ER), anatomical or developmental expression pattern (e.g., leaf, ovule, flower stage 10, seedling stage), or its partner in a protein-protein interaction (e.g., AT1G01010 interacts with AT1G01020).

If your paper contains results falling into one or more of these categories for Arabidopsis genes, we request that you now submit these data for inclusion in TAIR by filling in the form provided at the following URL: https://www.arabidopsis.org/doc/submit/functional_annotation/123. If you need further clarification on what types of data can be submitted please contact curator@arabidopsis.org.

Finally, we encourage your submission of artwork for the journal cover. If there is an image or illustration related to your paper that you would like to have considered, please email it in .pdf or .tif format to clowe@aspb.org. An image title, brief, 2-3 sentence description of the image, and the name of the person credited for the image are also encouraged. If your image is selected to appear on the online cover of the journal, you will be contacted with the scheduled issue date and further information.

We look forward to seeing your paper published.

On behalf of the editorial board,

Yuling Jiao, Board of Reviewing Editors
Siobhán Brady, Senior Editor
Blake Meyers, Editor-in-Chief

The Plant Cell

=====
IMPORTANT REMINDER: PEER REVIEW REPORTS
=====

If you opted to publish a peer review report along with your article during the original submission process, it will be prepared by the editorial staff and publicly posted with your manuscript, inside the zip file that contains any other supplemental material. As a reminder, the peer review report is a public record of all comments from editors and reviewers, as well as your prior responses, as you received them in the decision letters for each draft of your manuscript. If you agreed to publish this report and have changed your mind, or are not sure if you selected this option, please contact the editorial office as soon as possible before signing the license agreement from our publisher.

=====
REVIEWER COMMENTS
=====

Reviewer #2 (Comments for the Author):

The authors have performed sufficient complementary experiments that further supports their initial claims. At this stage, I would recommend that this paper be published in The Plant Cell.

Reviewer #3 (Comments for the Author):

Souvik Dhar and the colleagues in Ji-Young Lee's group have made a thorough revision of the work, corroborating the notion of STZ-mediated signalling in root development contributing to PEP1-induced growth retardation. Mostly importantly, this signalling pathway is important for plants to deal with stresses, for which the authors utilize different concentration of PEP1. While the role of PEP1 in inducing general defense in both shoot and root is supported by a series of previous literature, this work nicely demonstrate this defense is annexed with STZ-governed developmental pathways, which plants utilize to fine-tune the growth-defense trade-off. The highlight of the revision, in my opinion, was the priming experiments to demonstrate the dose-dependent STZ contribution to stress responses that renders plants more resilient to the imposed stress. This approaches leverage the strength of transgenic studies of Arabidopsis to reinforce the idea of quantitative realm of stress responses, towards which the plant biology field should be heading instead of focusing on strict qualitative assessment of stress responses.
The current version of manuscript has incorporated most reviewers' comments positively to convey the proposed ideas and supporting data effectively.

There are only few minor suggestions detailed below, which the authors might consider before making the final version for publication. Look forward to seeing this work online sooner than later.

1. Regarding the title, would the authors consider specifying the "danger signals" as "PEP1-induced signals"? I also see the two articles "the" in the title, which gives an impression of overstatement in current form. If the authors change the "signal" part, the whole title would sound pertinent to the work.

2. Figure 1A
The letters "M" in red circles shall be representing mock treatments. Please indicate this notion along with the condition of mock treatment in the figure legend to help readers appreciate the experimental conditions.

3. The CRISPR/Cas9 results of stz (as compared with other homologs) can be better discussed in terms of losing the control of trade-off. If STZ-mediated signalling is important in balancing growth and defense and fine-tuning this process depending on the strength of the external/internal stimuli, what happens indeed in the root when this modulation is absolutely gone? Is it a total tissue collapse or self-defense system overtly activated? No need to provide any experimental results, but discussion shall be elaborated to place STZ in a central place of this regulation.

----FOR ASPB OFFICE USE ONLY (DO NOT EDIT)----
MSID: 39282
Corresponding Author: Prof. Lee
